# Trends Analysis of Non-Hodgkin Lymphoma at the National, Regional, and Global Level, 1990–2019: Results From the Global Burden of Disease Study 2019

**DOI:** 10.3389/fmed.2021.738693

**Published:** 2021-09-23

**Authors:** Wenwen Cai, Qingle Zeng, Xingxing Zhang, Weiqing Ruan

**Affiliations:** ^1^Huiqiao Medical Center, Nanfang Hospital, Southern Medical University, Guangzhou, China; ^2^School of Nursing, Southern Medical University, Guangzhou, China; ^3^Department of Interventional Radiology, Nanfang Hospital, Southern Medical University, Guangzhou, China

**Keywords:** non-Hodgkin lymphoma, global burden of disease, age-standardized rate, estimated annual percentage change, epidemiological trend

## Abstract

**Background:** Non-Hodgkin lymphoma is a common hematologic malignancy. This article aimed to estimate the trends of non-Hodgkin lymphoma (NHL) globally from 1990 to 2019.

**Methods:** Data on the NHL burden were explored from the Global Burden of Disease study 2019. The trends of NHL burden were estimated using age-standardized rate (ASR) and estimated annual percentage change (EAPC).

**Results:** The ASR of NHL incidence showed an increasing trend worldwide from 1990 to 2019, with an EAPC of.56 [95% CI: 0.45–0.66]. Meanwhile, increasing trends were observed in both sexes and in most geographic regions, particularly East Asia (EAPC = 3.57, 95% CI: 3.29–3.86). The most pronounced increasing trends were seen in Georgia (EAPC = 4.7, 95% CI: 4.20–5.21), followed by Belarus and Uzbekistan. However, death and disability-adjusted life years (DALYs) caused by NHL showed decreasing trends globally, in which the respective EAPCs were −0.09 (95% CI: −0.17 to −0.02) and −0.28 (95% CI: −0.35 to −0.22). Decreasing trends were mainly seen in high and high-middle sociodemographic index (SDI) areas. At the national level, the largest increasing trends of death and DALYs were observed in Georgia, in which the respective EAPCs were 4.54 (95% CI: 4.01–5.07) and 4.97 (95% CI: 4.42–5.52).

**Conclusions:** Decreasing trends of death and DALYs caused by NHL were observed worldwide from 1990 to 2019, but NHL remains a substantial challenge globally. The findings would inform the strategies for reducing the burden of NHL.

## Introduction

Non-Hodgkin lymphoma is a kind of lymphoma characterized by a wide range of morbid features and difficulties in diagnosis (1) and accounts for 90% of all lymphoma incidences (2).

According to the Global Cancer Statistics 2018, the burden of non-Hodgkin lymphoma (NHL) aggravated in many regions, and reached 509,590 incidences and 248,724 deaths in 2018, accounting for 2.8 and 2.6%, respectively, of the total incidence and mortality of 36 cancers involved (3). In the United States, for example, the incidence of NHL has increased significantly over the past few decades, and now accounts for ~4% of total cancer incidence (4). Conversely, improved survival in NHL was commonly reported in recent years. In the 1970s, just over one-fifth of people with NHL survived more than 10 years, but now the proportion has increased to almost two-thirds (5). Of these, survival in the UK has seen a 3-fold increase in the last 40 years (5). The reasons for the increasing NHL incidence involve many social factors. The major risk factors for the development of NHL are various, including AIDS infection, immunosuppressive drugs, occupational exposures, pesticide contiguity, and smoking (6–9). In terms of the typologies of NHL, it has been studied that most NHL evolved from mature B lymphocytes, with a few from T lymphocytes or natural killer (NK) cells (10). In Western countries, the majority of NHL cases are B-cell lymphomas (~85%), but T-cell lymphoma is less common (~15%), while the reversed proportion occurs in developing regions (5, 11), which probably explains the significant geographic heterogeneity in the distribution of NHL burden.

The Global Burden of Disease study (GBDs) comprehensively assessed the burden caused by cancers globally (12, 13), which facilitated the tracking of the temporal trends of the disease burden. Strategies such as adjusting causes of death to compensate for incomplete data, developing maps to adjust study data, reassigning garbage codes, refining age groupings, and developing criteria to exclude outliers are used to ensure data quality and improve comparability (14). Therefore, this article aimed to estimate the trends of NHL burden from 1990 to 2019 using the data derived from the GBDs 2019 to provide information on health strategies.

## Materials and Methods

### Data Sources

Data on the NHL burden, such as incidence, death, and DALYs, were explored using the Global Health Data Exchange query tool (http://ghdx.healthdata.org/gbd-2019). According to the instruction of GBD online tools, data were extracted by sex, age group, geographic region, and country/territory from 1990 to 2019. The data were available in 21 geographic regions (e.g., East Asia, Australia, and the Caribbean) and 204 countries/territories (e.g., Slovenia, Thailand, and Germany) worldwide. The sociodemographic index (SDI) is a compound measure of social development and health outcomes. According to the SDI, these regions and countries/territories were classified into low, low-middle, middle, high-middle, and high. The human development index (HDI) is a compound measure reflecting the level of economy and human well-being, which was acquired from the United Nations Development Program (http://hdr.undp.org/en/data).

### Statistical Analysis

When comparing the disease distribution of several different age structures or the same population over a period of time with different age structures, age standardization is necessary to adjust for potential age structure confusion. Age-standardized rate (ASR) (/100,000 people) is calculated using the following formula:


$$\text{ASR=}\frac{\sum_{\text{i=1}}^{\text{A}}{\text{a}}_{\text{i}}{\text{w}}_{\text{i}}}{\sum_{\text{i=1}}^{\text{A}}{\text{w}}_{\text{i}}}\times \text{100},\text{000}$$

Age-standardized rate is equal to the sum of the products of *a*_*i*_ (the age-specific rates, where *i* denotes the *i*^th^ age class) and *w*_*i*_(the number of persons or weight) in the same age group *i* of the chosen reference standard population, then divided by the sum of standard population weights.

The estimated annual percentage change is equal to the annual change over the specified range and is calculated on a linear scale. The natural logarithm of the regression-line is fitted to ASR with the following formula: *y* = α + β *x* + ε, where y = ln (ASR), and *x* = calendar year. Estimated annual percentage change (EAPC) and its 95% CI were calculated as 100 × [exp(β)−1] with the linear regression model (15, 16). The trends were determined as follows: when both the EAPC and its lower limit of 95% CI were >0, it meant there was an increasing trend; when both the EAPC and its upper 95% CI were <0, it meant there was a decreasing trend; others meant that ASR was stable over time. In order to explore the influencing factors of EAPC, the relationships between EAPCs and ASR in 1990 and EAPCs and HDI in 2019 were estimated by a Pearson correlation analysis. Data were analyzed using R v3.6.2 (Institute for Statistical Computing, Vienna, Austria), and a value of *p* < 0.05 was considered to be statistically significant.

## Results

### Trends in Incidence of NHL

**Figure 1:**
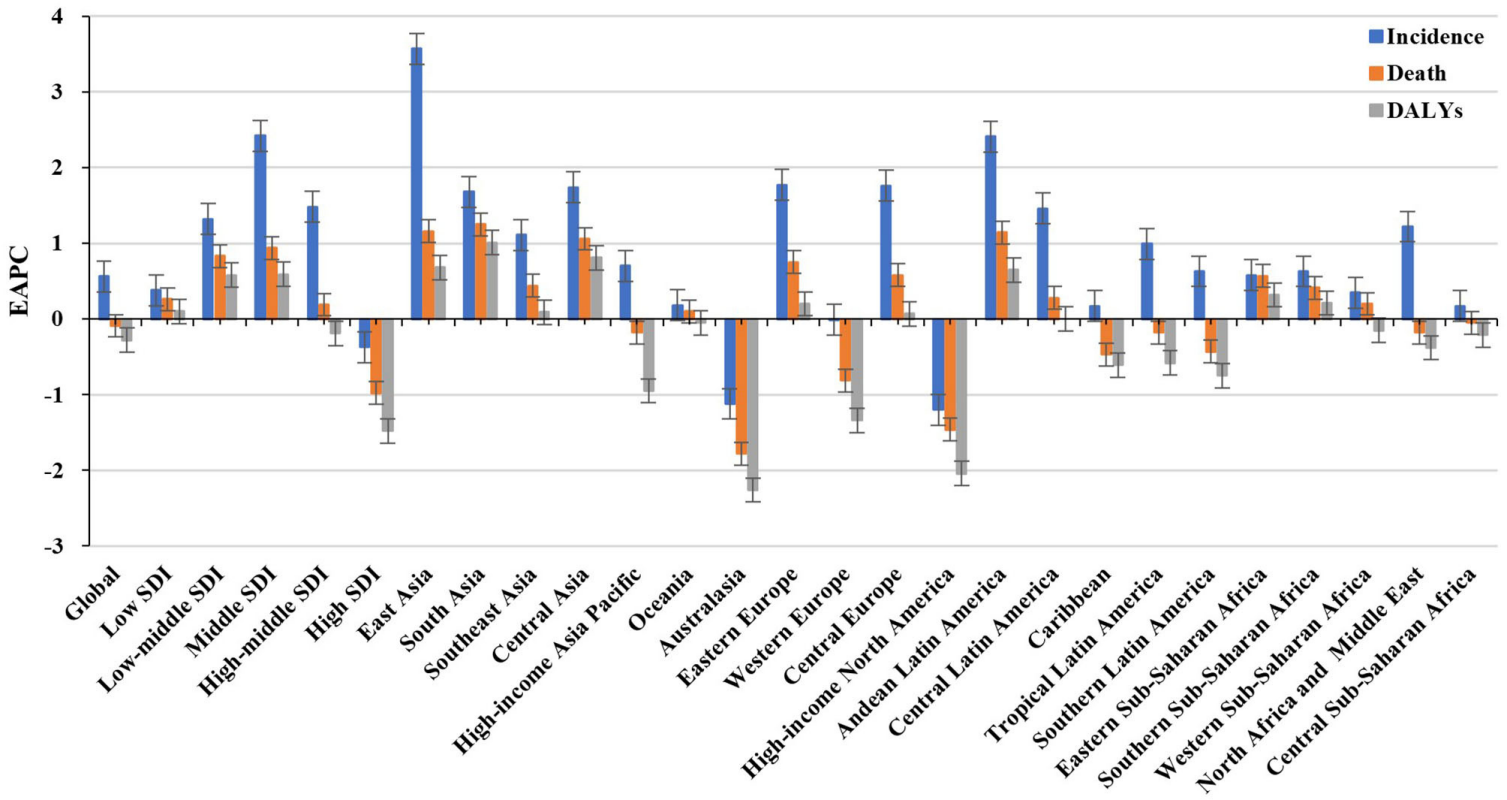
Trends in the incidence, death, and DALYs of non-Hodgkin lymphoma in the globe, SDI areas, and geographic regions from 1990 to 2019. SDI, sociodemographic index; DALYs, disability-adjusted life years.

**Figure 2:**
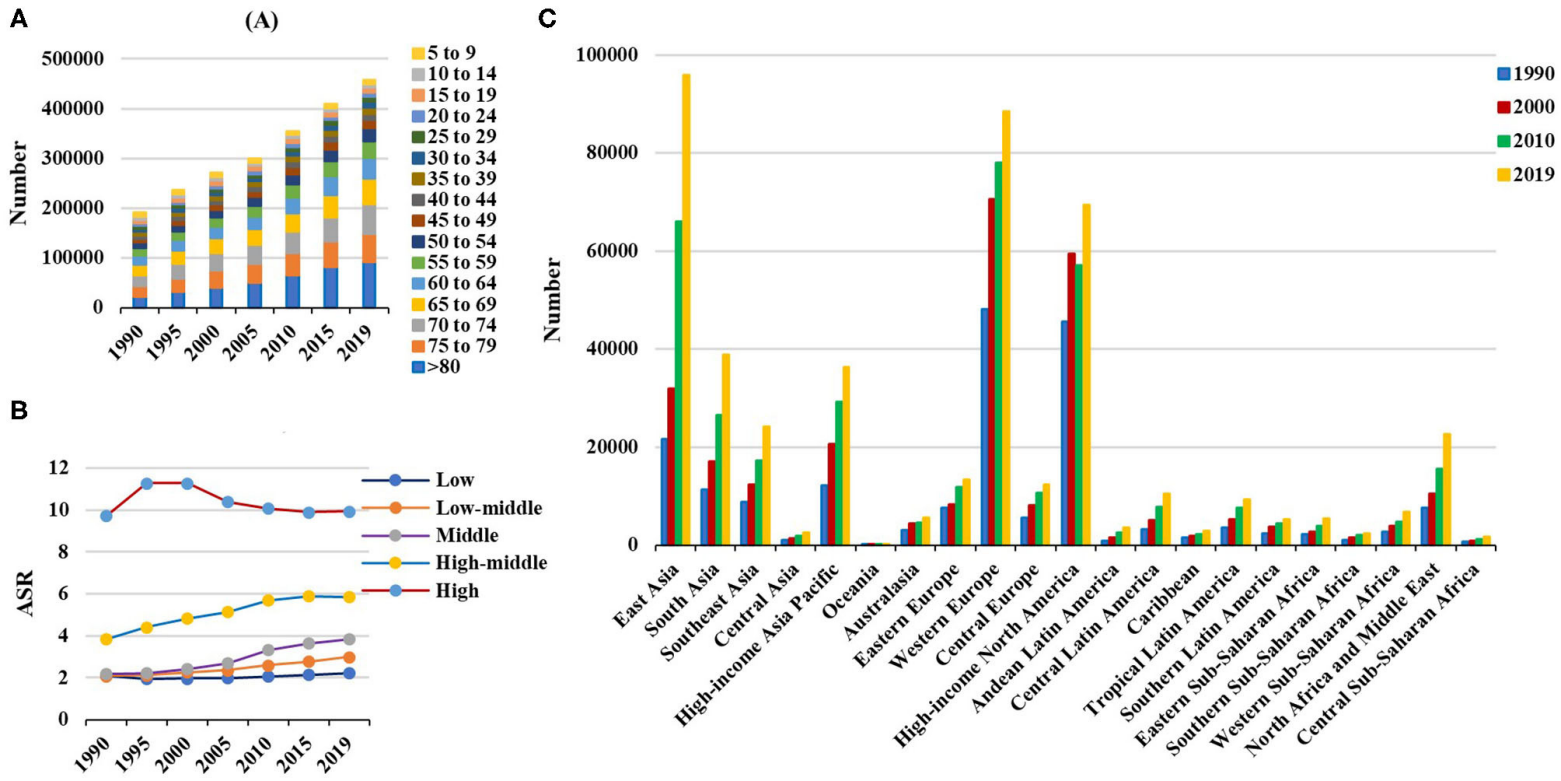
Incident number of non-Hodgkin lymphoma in age groups, SDI areas, and geographic regions from 1990 to 2019. **(A)** Incident number in age groups; **(B)** ASIR in SDI areas; **(C)** incident number in geographical regions. SDI, sociodemographic index; ASIR, age-standardized incidence rate.

**Figure 3:**
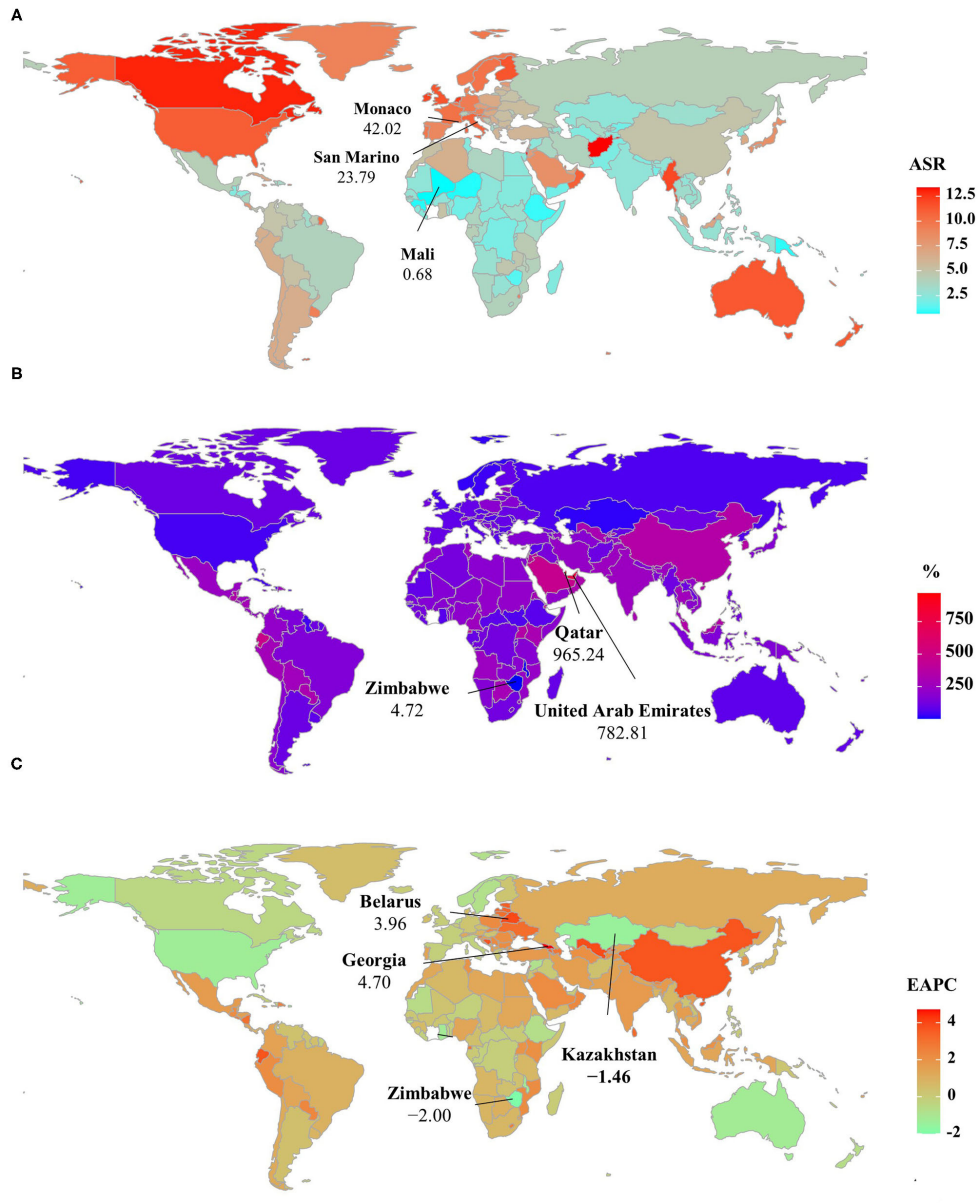
ASIR, percentage changes in the incident number, and EAPCs of non-Hodgkin lymphoma at the national level, 1990–2019. **(A)** ASIR in 2019; **(B)** percentage changes in incident numbers between 2000 and 2019; **(C)** distribution of EAPCs presented in countries/territories. Countries/territories with extreme values were annotated. ASIR, age-standardized incidence rate; EAPC, estimated annual percentage change.

Globally, the incident number of NHL was 457.08 × 10^3^ [95% uncertainty interval (UI): 416.89 × 10^3^ to 498.78 × 10^3^] in 2019, with an increase of 139.65% since 1990. The age-standardized incidence rate (ASIR) showed an increasing trend worldwide from 1990 to 2019 (EAPC = 0.56, 95% CI: 0.45– 0.66) (Table 1, Figure 1). Compared with female patients, an increasing trend in ASIR was more pronounced in male patients (EAPC = 0.78, 95% CI: 0.69– 0.87) (Table 1). The highest incident number was seen in group aged above 80 in 2019 (91.33 × 103), and an increasing percentage in incident number was found in all age groups except for the group aged below 5 years old from 1990 to 2019 (Supplementary Table 1, Figure 2A). Increasing trends in ASIR were observed in four SDI areas, particularly in the middle ones (EAPC = 2.42, 95% CI: 2.26–2.58); whereas a decreasing trend was seen in high SDI areas (EAPC = −0.37, 95% CI: −0.56 to −0.18). At the regional level, East Asia was the region with the highest incident cases (95.93 × 10^3^) in 2019, with an increasing percentage of 343.84% since 1990. Increasing trends in ASIR were observed in 17 regions, and the most pronounced ones were in East Asia and Andean Latin America, in which the respective EAPCs were 3.57 (95% CI: 3.29–3.86) and 2.41 (95%CI: 2.24–2.57). Downward trends in ASIR were only seen in high-income North America and Australasia, whose EAPCs were −1.2 (95% CI: −1.43 to −0.97) and −1.12 (95% CI: −1.39 to −0.85), respectively (Table 1, Figures 1, 2B,C). At the national level, the ASIR in 2019 varied from 0.68/100,000 in Mali to 14.86/100,000 in Andorra. During the period 1990–2019, the largest growing percentages of incident number occurred in Qatar (965.24%) and the United Arab Emirates (782.81%), but the lowest one was in Zimbabwe (4.72%). One hundred fifty countries/territories showed varying degrees of increase in ASIR, particularly Georgia and Belarus, in which the respective EAPCs were 4.7 (95% CI: 4.20–5.21) and 3.96 (95% CI: 3.47–4.44); whereas 28 countries/territories showed decreasing trends, with the lowest EAPCs occurring in Zimbabwe (EAPC = −2, 95% CI: −2.31 to −1.69), followed by Kazakhstan and the United States of America (Supplementary Table 2, Figures 3A–C).

**Table 1:** Number and age-standardized rate of non-Hodgkin lymphoma incidence on a global scale by sex, SDI areas, and geographic regions in 1990 and 2019, and the percentage change of absolute number and the EAPCs from 1990 to 2019.

	**1990**		**2019**		**1990–2019**	
**Characteristics**	**Number ×10^**3**^ (95% UI)**	**ASR/100,000 (95% UI)**	**Number ×10^**3**^ (95% UI)**	**ASR/100,000 (95% UI)**	**Percentage change (%)**	**EAPC (95%CI)**
Overall	190.73 (179.03–203.62)	4.65 (4.37–4.93)	457.08 (416.89–498.78)	5.73 (5.21–6.25)	139.65	0.56 (0.45–0.66)
**Sex**						
Male	108.17 (101.46–116.46)	5.64 (5.31–6)	266.09 (241.38–291.09)	7.20 (6.55–7.88)	146.00	0.78 (0.69–0.87)
Female	82.56 (76.85–88.72)	3.79 (3.52–4.05)	190.98 (169.14–210.59)	4.45 (3.95–4.91)	131.33	0.25 (0.11–0.40)
**SDI**						
Low	6.19 (5.11–7.70)	2.10 (1.82–2.42)	14.55 (11.99–17.7)	2.19 (1.83–2.62)	135.21	0.38 (0.29–0.48)
Low-middle	15.89 (13.37–19.49)	2.09 (1.84–2.43)	44.48 (39.6–50.68)	2.99 (2.68–3.38)	180.02	1.32 (1.26–1.39)
Middle	28.46 (25.4–32.02)	2.18 (1.99–2.40)	94.44 (84.78–105.25)	3.84 (3.46–4.27)	231.89	2.42 (2.26–2.58)
High-middle	41.73 (39.17–44.57)	3.83 (3.59–4.09)	109.88 (98.18–122.32)	5.85 (5.24–6.48)	163.30	1.48 (1.35–1.60)
High	98.35 (92.37–104.10)	9.71 (9.15–10.25)	184.16 (161.68–206.62)	9.93 (8.84–11.11)	87.25	−0.37 (−0.56 to −0.18)
**Regions**						
East Asia	21.61 (18.55–25.57)	2.12 (1.85–2.46)	95.93 (81.21–113.06)	5.04 (4.31–5.89)	343.84	3.57 (3.29–3.86)
South Asia	11.36 (9.17–14.82)	1.56 (1.32–1.88)	38.92 (32.86–47.02)	2.53 (2.15–3.01)	242.60	1.68 (1.61–1.74)
Southeast Asia	8.83 (7.57–10.28)	2.77 (2.44–3.17)	24.13 (20.67–28.16)	3.88 (3.34–4.49)	173.31	1.11 (1.07–1.16)
Central Asia	1.00 (0.87–1.18)	1.73 (1.54–1.99)	2.45 (2.12–2.84)	2.96 (2.58–3.39)	144.32	1.74 (1.55–1.92)
High-income, Asia Pacific	12.23 (11.5–12.94)	6.30 (5.93–6.66)	36.24 (30.09–42.22)	8.06 (6.89–9.34)	196.31	0.70 (0.51–0.89)
Oceania	0.06 (0.05–0.07)	1.60 (1.37–1.84)	0.14 (0.12–0.17)	1.71 (1.46–2.03)	135.87	0.18 (0.11–0.25)
Australasia	3.01 (2.79–3.24)	12.95 (12.03–13.91)	5.54 (4.38–6.87)	11.31 (8.97–14.07)	83.95	−1.12 (−1.39 to −0.85)
Eastern Europe	7.62 (7.10–8.23)	3.05 (2.83–3.31)	13.38 (11.91–14.96)	4.61 (4.12–5.13)	75.60	1.77 (1.48–2.07)
Western Europe	48.09 (45.12–50.89)	8.95 (8.44–9.43)	88.44 (75.34–101.72)	10.11 (8.65–11.61)	83.88	−0.01 (−0.23–0.22)
Central Europe	5.54 (5.18–5.93)	3.99 (3.73–4.27)	12.27 (10.74–14.12)	6.49 (5.71–7.43)	121.54	1.76 (1.55–1.96)
High–income North America	45.61 (42.72–48.3)	13.22 (12.42–14)	69.46 (59.74–79.49)	11.28 (9.74–12.9)	52.29	−1.20 (−1.43 to −0.97)
Andean Latin America	0.86 (0.76–0.97)	3.34 (2.99–3.73)	3.59 (2.91–4.48)	6.21 (5.03–7.76)	318.27	2.41 (2.24–2.57)
Central Latin America	3.23 (3.01–3.5)	2.82 (2.66–3.02)	10.40 (8.91–12.17)	4.32 (3.71–5.05)	222.07	1.46 (1.33–1.59)
Caribbean	1.59 (1.47–1.71)	5.39 (5.02–5.78)	2.82 (2.37–3.32)	5.58 (4.69–6.58)	76.95	0.17 (0.02–0.32)
Tropical Latin America	3.51 (3.28–3.8)	3.04 (2.87–3.25)	9.27 (8.59–9.99)	3.92 (3.63–4.22)	164.46	0.99 (0.80–1.18)
Southern Latin America	2.37 (2.18–2.57)	5.02 (4.61–5.42)	5.30 (4.15–6.73)	6.64 (5.19–8.44)	123.35	0.63 (0.34–0.92)
Eastern Sub- Saharan Africa	2.16 (1.74–2.67)	2.41 (2.08–2.76)	5.41 (4.49–6.55)	2.78 (2.37–3.27)	150.53	0.58 (0.49–0.66)
Southern Sub-Saharan Africa	1.09 (0.97–1.21)	3.03 (2.69–3.35)	2.35 (2.05–2.67)	3.58 (3.16–4.02)	115.50	0.63 (0.48–0.78)
Western Sub- Saharan Africa	2.62 (2.01–3.53)	2.00 (1.63–2.44)	6.76 (5.11–9.00)	2.32 (1.88–2.85)	157.65	0.35 (0.24–0.47)
North Africa and Middle East	7.67 (6.32–9.3)	3.40 (2.87–3.97)	22.61 (19.76–25.81)	4.72 (4.15–5.38)	194.83	1.22 (1.08–1.36)
Central Sub- Saharan Africa	0.66 (0.52–0.81)	2.26 (1.74–2.82)	1.67 (1.18–2.26)	2.34 (1.61–3.32)	154.33	0.17 (−0.01–0.35)

*EAPC, estimated annual percentage change; ASR, age-standardized rate; CI, confidence interval; UI, uncertainty interval; SDI, sociodemographic index*.

### Trends in Death Caused by NHL

Over the past 30 years, deaths due to NHL in the world increased steadily by increased 101.94% and reached up to 254.61 × 10^3^ (95% UI: 237.71 × 10^3^ to 270.35 × 10^3^) in 2019. The overall age-standardized death rate (ASDR) presented a decreasing trend from 1990 to 2019 (EAPC = −0.09, 95% CI: −0.17 to −0.02) (Supplementary Table 3, Figure 1). A slight increasing trend in ASDR was observed in male patients (EAPC = 0.08, 95% CI: 0.01– 0.15), while a downward trend was observed in female patients (EAPC = −0.34, 95% CI: −0.44 to −0.23) (Supplementary Table 3). Among the age groups, the largest number of deaths was seen in the age group with participants over 80 years old in 2019 (58.77 × 10^3^). From 1990 to 2019, the increasing percentages in death were observed in all age groups of above 15 years, with the most notable one showing in the age group of over 80 years (231.1%). However, the percentage declined in patients aged younger than 14 years (Supplementary Table 1, Supplementary Figure 1A). Upward trends were observed in most SDI areas, especially the middle SDI areas, with an EAPC of 0.94 (95% CI: 0.84–1.03). However, a decreasing tendency only occurred in high SDI areas (EACP = −0.98, 95% CI: −1.15 to −0.82). Among 21 regions, the highest death number in 2019 occurred in East Asia (46.45 × 10^3^), while the lowest one was in Oceania (0.13 × 10^3^). During the period 1990–2019, the largest increasing percentage was observed in Andean Latin America (218.58%) and South Asia (210.46%). Increasing trends in ASDR were seen in 12 regions, and the most pronounced one was in South Asia (EAPC = 1.25, 95% CI: 1.18–1.32). On the other hand, decreasing trends were observed in eight regions, particularly in Australasia (EAPC = −1.78, 95%CI: −2.01 to −1.56) (Supplementary Table 3, Figure 1, Supplementary Figures 1B,C). At the national level, the highest ASDR in 2019 occurred in San Marino (9.56/100,000), while the lowest one occurred in Mali (0.7/100,000). During the period 1990–2019, the largest increases in numbers of deaths were in the United Arab Emirates (609.26%) and in Qatar (491.98%), but the pronounced decreasing one was in Niue (−2.86%). Increasing trends in ASDR occurred in 102 countries/territories, with the most significant one occurring in Georgia (EAPC = 4.54, 95% CI: 4.01–5.07), followed by Uzbekistan and Belarus. However, decreasing trends appeared in 75 countries/territories, particularly in the Syrian Arab Republic and Bermuda, in which the EAPCs were −2.46 (95% CI: −3.05 to −1.86) and −2.21 (95% CI: −2.46 to −1.96), respectively (Supplementary Table 4, Supplementary Figures 3A–C).

### Trends in DALYs Caused by NHL

Globally, the DALYs number of NHL has increased by 68.59% since 1990, and it went by up to 6991.3 × 10^3^ (95% UI: 6570.14 × 10^3^ to 7450.47 × 10^3^) in 2019. The overall ASR of DALYs declined from 1990 to 2019, with an EAPC of −0.28 (95% CI: −0.35 to −0.22). Trends in the ASR of DALYs had a minor increase in male patients but had a decrease in female patients (EAPC = −0.34, 95% CI: −0.44 to −0.23) (Supplementary Table 5, Figure 1). Among the age groups, the largest number of DALYs in 2019 was seen in patients aged 65–69 years (733.13 × 10^3^). During the period 1990–2019, the percentage of DALYs number decreased in age groups younger than 14 years, but decreased in the rest, particularly in the age group of above 80 years (226.13%) (Supplementary Table 1; Supplementary Figure 2A). Increasing trends in the ASR of DALYs were observed in all the SDI areas, except the high SDI area (EACP = −1.48, 95% CI: −1.65 to −1.31). Among 21 geographic regions, the highest DALYs number in 2019 was seen in East Asia (1362.18 × 10^3^), and the lowest one was seen in Oceania (5.08 × 10^3^). During the period 1990–2019, the largest increasing percentage was observed in Andean Latin America (138.6%) and South Asia (149.70%). Increasing trends in the ASR of DALYs were found in 11 regions, especially South Asia and Central Asia, with EAPCs of 1.01 (95% CI:0.94 to 1.09) and.81 (95% CI:0.62–1.01), respectively. However, decreasing trends were demonstrated in six regions, particularly in high-income North America (EAPC = −2.04, 95%CI: −2.27 to −1.82) (Supplementary Table 5, Figure 1, Supplementary Figures 2B,C). Among the 204 countries/territories, the ASR of DALYs in 2019 ranged from 23.53/100,000 in Mali to 409.87/100,000 in Monaco. From 1990 to 2019, the largest increases in DALYs were in the United Arab Emirates (561.34%) and in Qatar (471.66%), but the pronounced decreasing one was in Mali (−39.28%). Increasing trends in the ASR of DALYs were observed in 80 countries/territories, and the most pronounced ones were in Georgia (EAPC = 4.97, 95% CI: 4.42 to 5.52), followed by Uzbekistan and Lesotho. On the other hand, decreasing trends were seen in 90 countries/territories, particularly Kazakhstan and Malawi, with EAPCs of −2.92 (95%CI: −3.59 to −2.25) and −2.71 (95%CI: −2.95 to −2.47) (Supplementary Table 6, Supplementary Figures 4A–C), respectively.

### Analysis on the Influential Factors of EAPC

**Figure 4:**
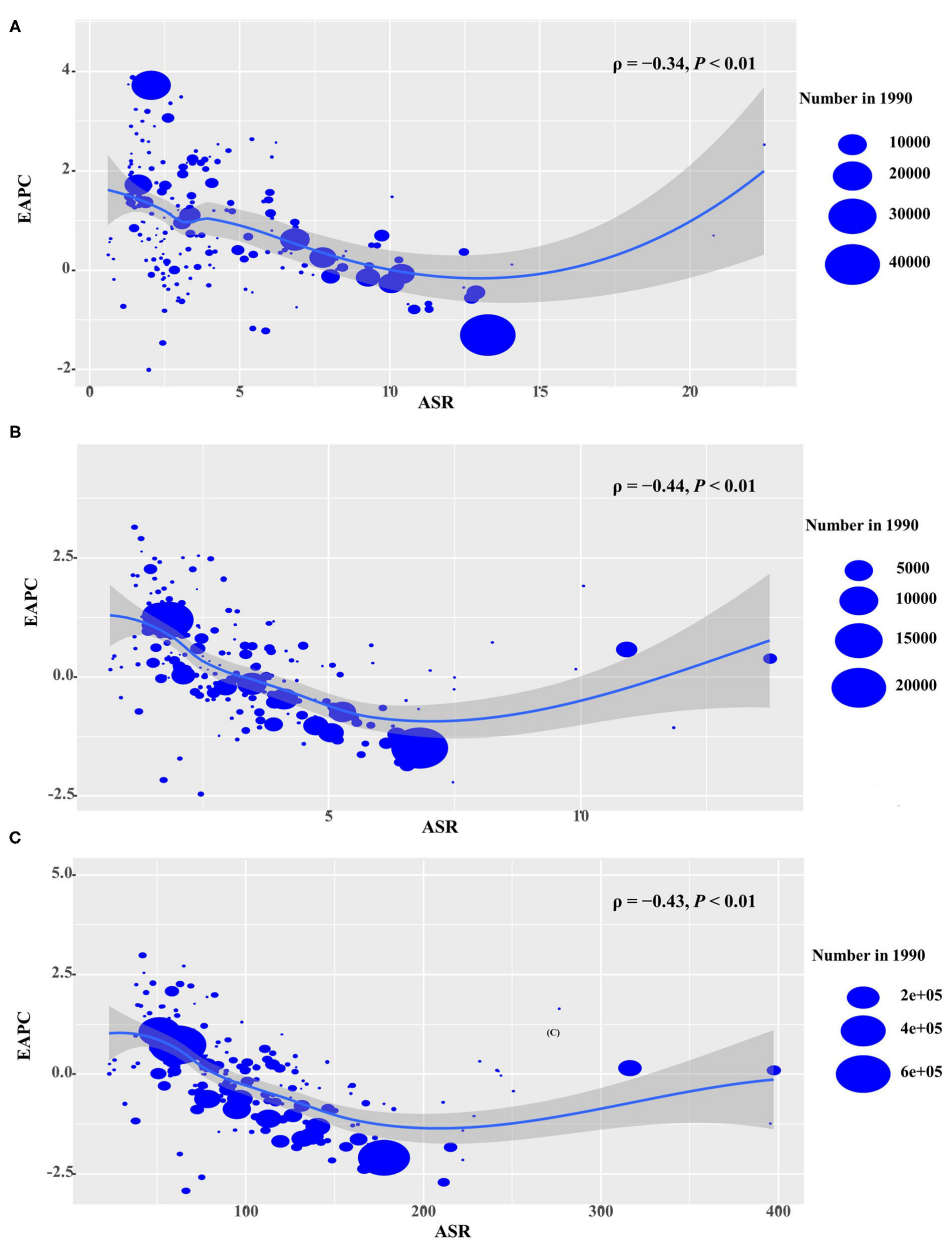
Correlation between EAPCs and ASR in 1990 at the national level. **(A)** EAPCs of incidence, **(B)** death, and **(C)** DALYs had negative associations with ASR in 1990. The association was calculated by a Pearson correlation analysis. The size of the circle is increased with the numbers in 1990. EAPC, estimated annual percentage change; ASR, age-standardized rate. DALYs, disability-adjusted life years.

For the period 1990–2019, EAPCs had negative relationships with the ASR of incidence, death, and DALYs attributable to NHL (ρ = −0.34, *p* < 0.01; ρ = −0.44, *p* < 0.01; ρ = −0.43, *p* < 0.01, respectively) (Figures 4A–C). In addition, EAPC was also negatively correlated with HDI in 2019 in DALYs attributable to NHL (ρ = −0.2, *p* = 0.005) (Supplementary Figure 5).

## Discussion

In this article, increasing trends in the ASIR of NHL were observed worldwide and in most regions and countries from 1990 to 2019, which were due to population growth and aging, diagnostic improvements, the AIDS epidemic, and environmental pollution such as organic matter and heavy metals caused by urban industrialization (17–21). The significantly increasing burden was observed in old patients with NHL, and was probably related with age, which is a strong negative factor in all lymphoma subtypes (22–24). In part, it is also affected by various co-morbidities (17), such as cancer, cardiovascular disease, and stroke (6). The decline in mortality and DALYs for children aged below 14 years compared with adults may be due to biological differences across the age groups. For example, in the case of diffuse large B-cell lymphoma (DLBCL) and anaplastic large cell lymphoma (ALCL), pediatric patients are more likely to have GCB disease (a main subtype of DLBCL) and anaplastic lymphoma kinase (+) ALCL (ALK^+^ ALCL), both of which have superior prognostic features of NHL (25–27). Thus, comparing pathogenesis patterns by NHL subtypes may provide key clues for future etiologic investigations (28). Meanwhile, the lower burden of NHL in female patients was similar to the previous epidemiological studies, in which 5- and 10-year survival was demonstrated to be generally higher in women (3, 29). However, the difference in survival is independent of race, ethnicity, year of diagnosis, or NHL subtypes (24), and this may be caused by different lifestyles between sexes or hormonal influences (22).

In recent years, the improved survival in NHL has been largely attributed to medical advances, such as the development and clinical application of effective treatments like rituximab (a monoclonal antibody)(30). However, the heterogeneity in NHL burden between regions and countries was deeply influenced by local economic development, disease prevalence (11), and local health systems (24). Interestingly, we observed a significant increase in the disease burden in some countries of the former Soviet Union, where Georgia showed the most pronounced trends in its increases in incidence, death, and DALYs, which may reflect the insufficient level of early HIV diagnosis; although medical and economic conditions have improved in recent years, more than half of the cases still do not receive timely and effective treatment (31, 32). The late-stage diagnoses of HIV also occurred in Europe, especially in cases of co-morbid NHL, where HIV detection rates are lower than in other diseases (33). In addition, the increased morbidity in Belarus may also be related to the high prevalence of HIV and the rapid spread of HIV variants in recent years (32). Nevertheless, the explanations still need to be supported by more in-depth studies because of the sparse data in the epidemiological literature on NHL in Eastern Europe and Central Asia. However, the incidence of NHL has significantly declined in developed countries, such as Australia and the United States, because of highly active antiretroviral therapy (HAART) (34, 35). Meanwhile, we also note an interesting point: the relative risk of HIV-associated NHL in whites is twice that of blacks in countries such as the United States, which may be due to differences in immune system regulatory genes caused by racial distinguishments (36). It is also certain that the decline in trend in Zimbabwe is also associated with the widespread availability of antiretroviral therapy or possibly with the loss of public health data (37).

Western diet and lifestyle caused by industrial and economic development might be responsible for the increasing NHL burden in East Asia (38–40). However, in contrast to the pattern of multiple lymph node disorders in Western countries, Asian countries exhibit intermediate-grade to high-grade diffuse aggressive lymphomas, peripheral T-cell NHL, and extra-lymph node disease more often (41). The poor medical source, misdiagnosis and omission by lacking high quality pathological diagnosis of NHL, and the heavy burden of chemotherapy drugs on the masses had brought the worse prognosis of NHL patients in South Asia (18, 42). In Central Asia, the situation of death, DALYs, and the burden of NHL is far more severe in Uzbekistan than in Kazakhstan, probably because of the fact that HIV genotyping studies have been conducted for a long time in Kazakhstan, while they were just beginning in Uzbekistan (32). In contrast, in South Africa, excluding major changes in society and the lifestyles of the people, scholars hypothesized that the development of NHL was delayed because of the receipt of various anti-infective treatments (43–46). Researchers have applied a low-intensity EPOCH (etoposide, prednisolone, vincristine, cyclophosphamide, doxorubicin) infusion regimen in sub-Saharan Africa, which may have contributed to the decline in DALYs in Malawi because the infusion regimen is a treatment that can improve outcomes for adolescents and adults with Burkitt lymphoma (BL) in Malawi and reduce drug toxicity reactions (47). There were several limitations in this study. First, the quality and quantity of data sources determined the accuracy and reliability of GBDs estimates; thus, potential bias probably derived from unreported cases, incomplete testing, miscoding, and misclassification, which were described in detail in the previous GBDs articles (12–14). Second, the diagnosis and classifications of NHL had been revised differently in various countries over time, and this has had a significant impact on data estimation, which was an important limitation. Third, the basis of estimating data for most middle- and low-income countries is largely derived from high-income countries with strong cancer registration systems, which still introduces some uncertainty in the veracity of the data due to the heterogeneity across countries. Last but not least, despite the use of tools to correct data, it is impossible for GBDs to fully control biases in data collection, entry, and veracity (14, 48).

## Conclusions

In the past three decades, slow decreasing trends in the deaths and DALYs of NHL were observed worldwide. Meanwhile, increasing incident trends in NHL highlighted that NHL burden remained a substantial challenge. Furthermore, the impact of NHL varies widely worldwide and is the result of a combination of factors, such as environmental and genetic, so performing more systematic comparative analyses of NHL by geographic region is warranted (36, 49). The findings also indicated that more effective strategies should be established to improve the management and treatment of patients with NHL.

## Data Availability Statement

The original contributions presented in the study are included in the article/Supplementary Material, further inquiries can be directed to the corresponding author/s.

## Author Contributions

WC: project administration and drafting. QZ: data analysis and validation. XZ: data analysis and visualization. WR: supervision, drafting, and editing. All the authors contributed to the article and approved the submitted version.

## Funding

This study was supported by the Science and Technology Planning Project of Guangdong Province of China (Grant Nos: 2015A070707005 and 2020A1414040014).

## Conflict of Interest

The authors declare that the research was conducted in the absence of any commercial or financial relationships that could be construed as a potential conflict of interest.

## Publisher's Note

All claims expressed in this article are solely those of the authors and do not necessarily represent those of their affiliated organizations, or those of the publisher, the editors and the reviewers. Any product that may be evaluated in this article, or claim that may be made by its manufacturer, is not guaranteed or endorsed by the publisher.

## Abbreviations

ASR: age-standardized rate
CI: confidence interval
DALYs: disability-adjusted life years
EAPC: estimated annual percentage change
GBD: Global Burden of Disease
GHDx: Global Health Data Exchange
NHL: non-Hodgkin lymphoma
SDI: sociodemographic index
UI: uncertainty interval.
