# Supplementary material for: Trends Analysis of Non-Hodgkin Lymphoma at the National, Regional, and Global Level, 1990–2019: Results From the Global Burden of Disease Study 2019
Source: Front Med (Lausanne). 2021 Sep 23;8:738693. doi: 10.3389/fmed.2021.738693 (PMC8494781; doi:10.3389/fmed.2021.738693)
Supplement: Supplementary file 1 [file Data_Sheet_1.docx]

**Supplementary figure 1**. The distribution of death caused by non-Hodgkin lymphoma in age groups, SDI areas, and geographic regions from 1990 to 2019. (A) the death number in age groups; (B) the ASDR in SDI areas; (C) the death number in geographical regions. SDI, sociodemographic index; ASDR, age-standardized death rate.

**
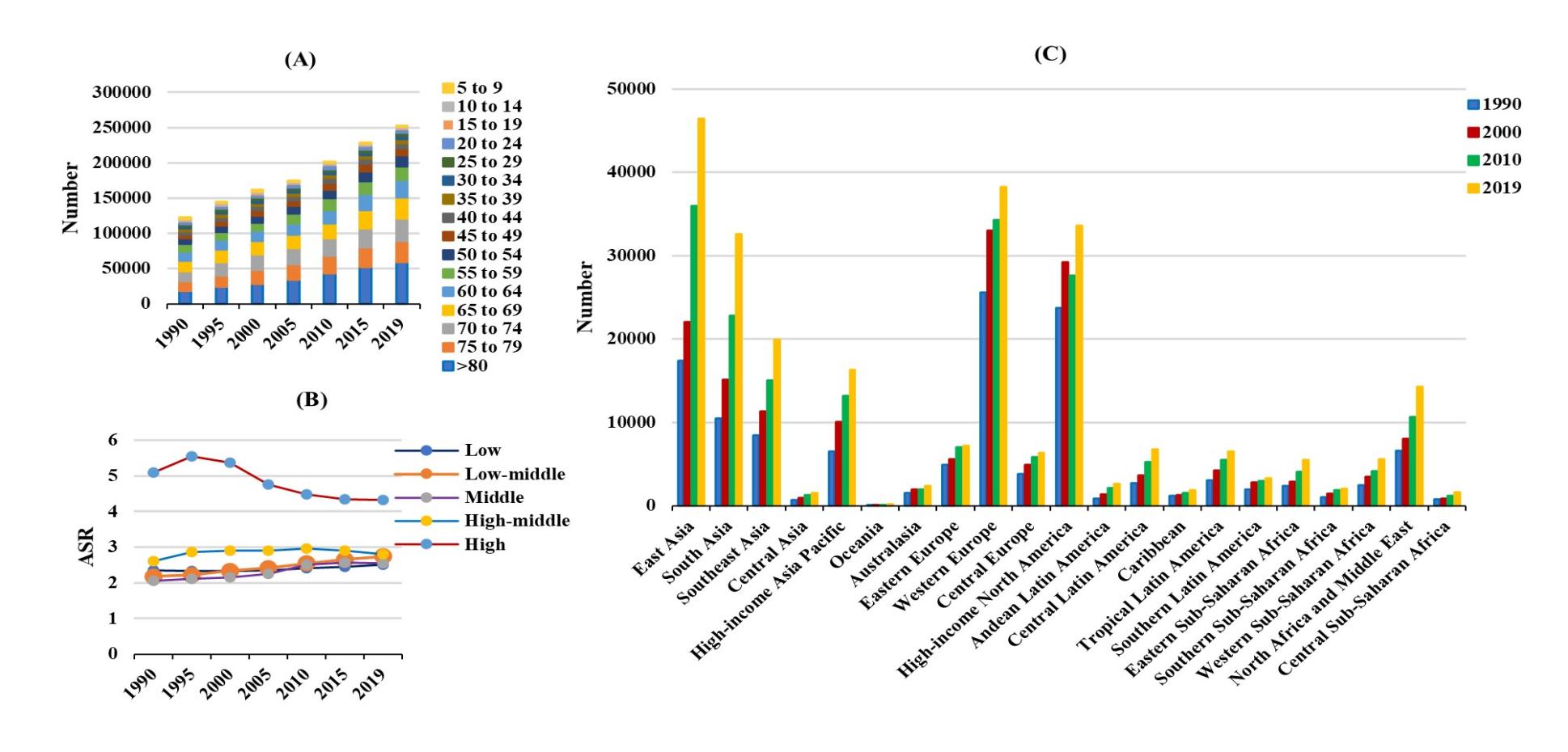
**

**Supplementary figure 2**. The distribution of DALYs caused by non-Hodgkin lymphoma in age groups, SDI areas, and geographic regions from 1990 to 2019. (A) the DALYs number in age groups; (B) the ASR of DALYs in SDI areas; (C) the number of DALYs in geographical regions. SDI, sociodemographic index. DALYs, disability-adjusted life years; ASR, age-standardized rate.

**
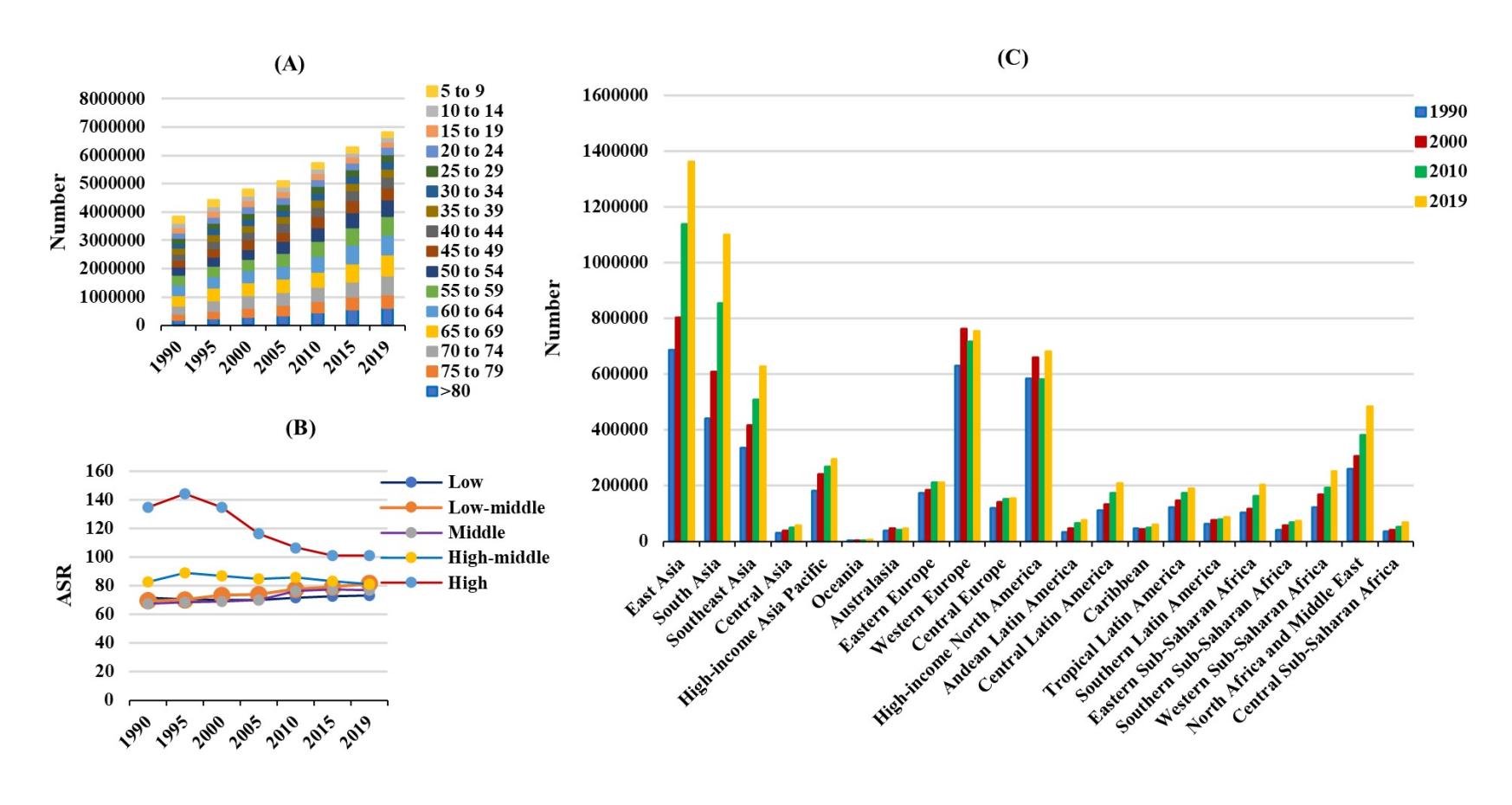
**

**Supplementary figure 3**. The distribution of ASDR, percentage changes in number, and EAPCs of death caused by non-Hodgkin lymphoma at the national level from 1990 to 2019. (A) the ASDR in 2019; (B) the percentage changes in number between 2000 and 2019; (C) the EAPCs in countries/territories from 1990 to 2019, respectively. Countries/territories with an extreme value were annotated. ASR, age-standardized death rate; EAPC, estimated annual percentage change.

**
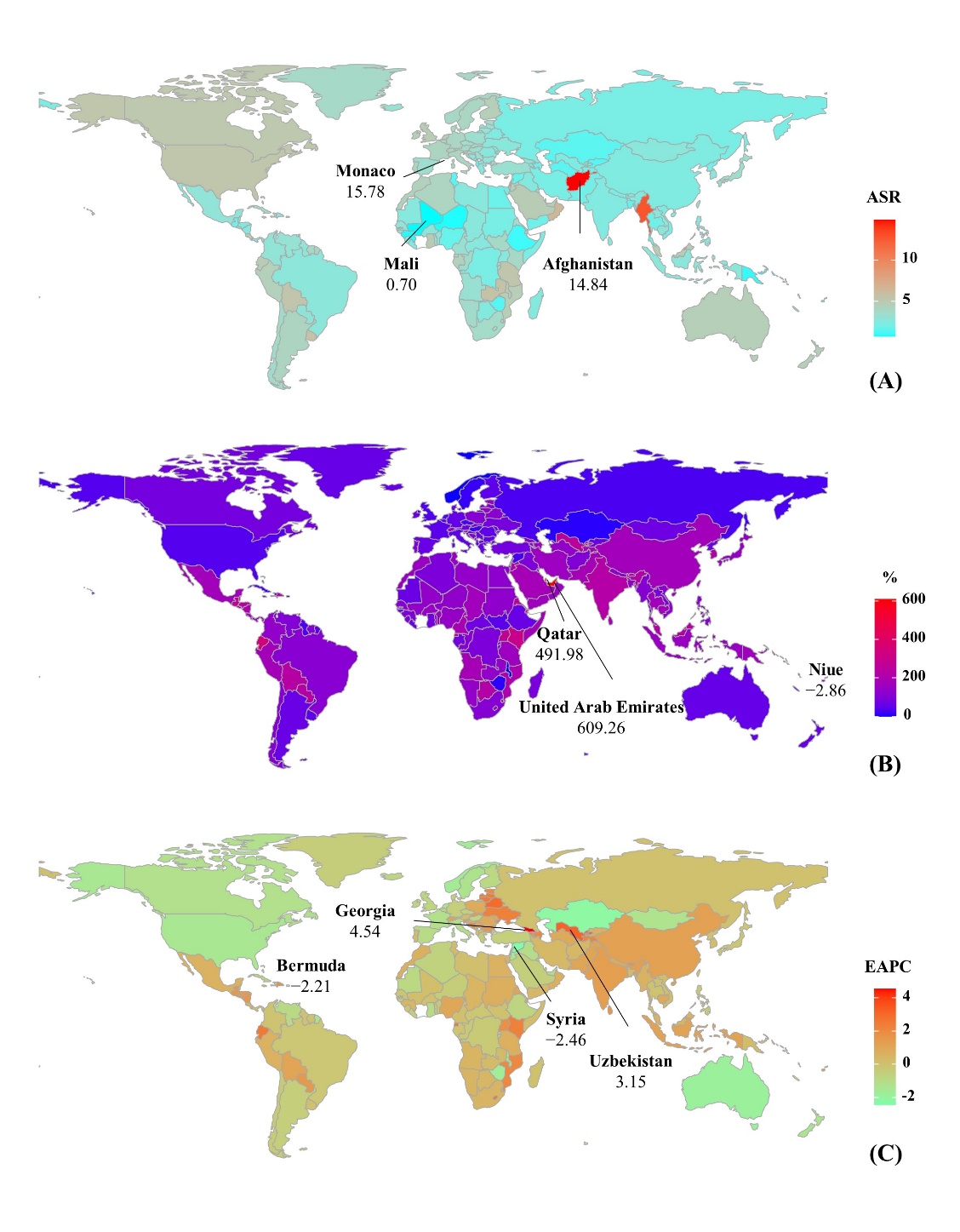
**

**Supplementary figure 4**. The distribution of ASR, percentage changes in number, and EAPCs of DALYs caused by non-Hodgkin lymphoma at the national level from 1990 to 2019. (A) the ASR in 2019; (B) the percentage changes in number between 2000 and 2019; (C) the EAPCs in countries/territories from 1990 to 2019, respectively. Countries/territories with an extreme value were annotated. ASR, age-standardized rate; EAPC, estimated annual percentage change; DALYs, disability-adjusted life years.

**
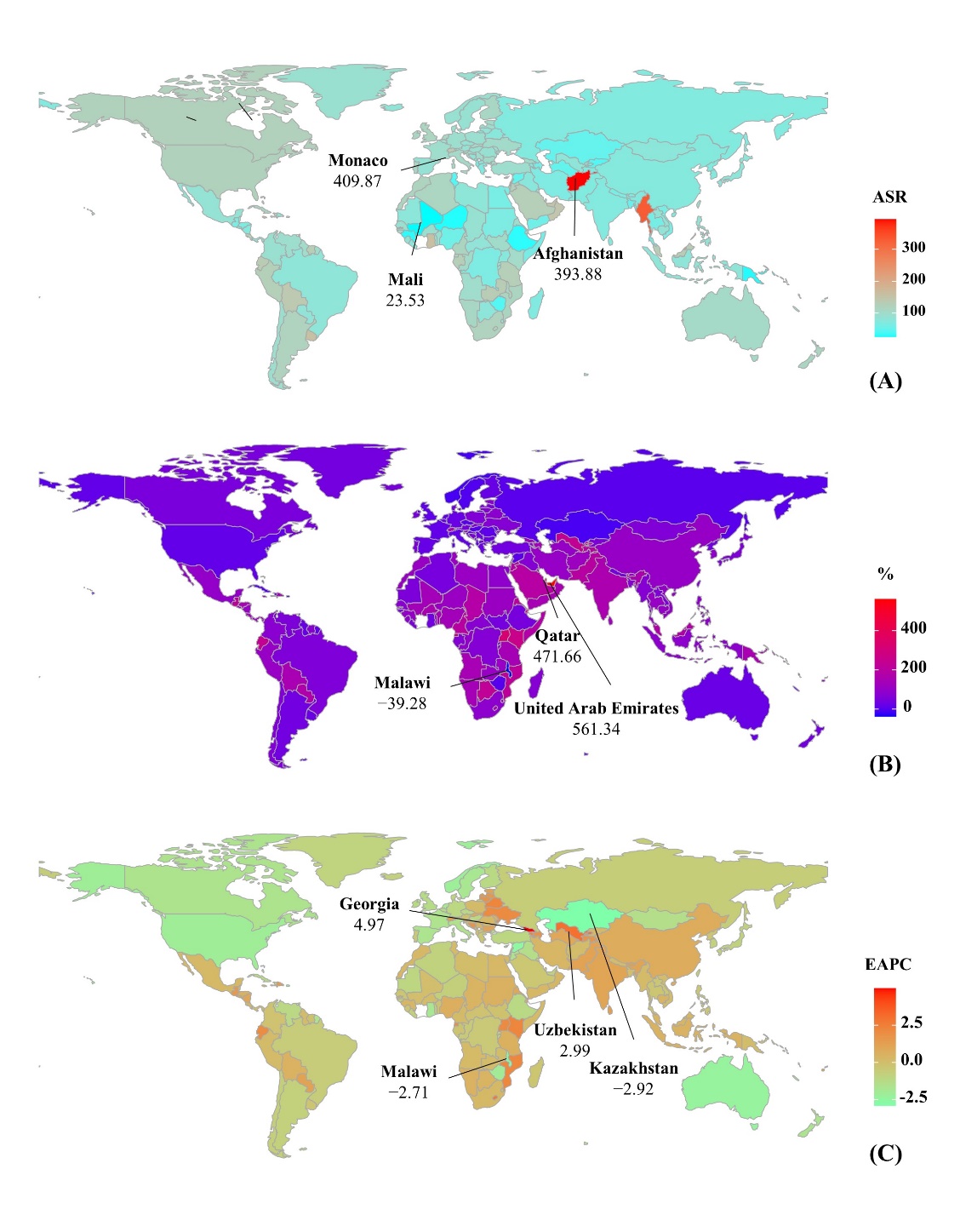
**

**Figure 5.** The correlation between EAPCs of DALYs and HDI in 2019 at the national level. The association was calculated with Pearson correlation analysis. The size of circle increased with the DALYs number in the corresponding countries/territories in 2019. EAPC, estimated annual percentage change; ASR, age-standardized rate. DALYs, disability-adjusted life years.

**
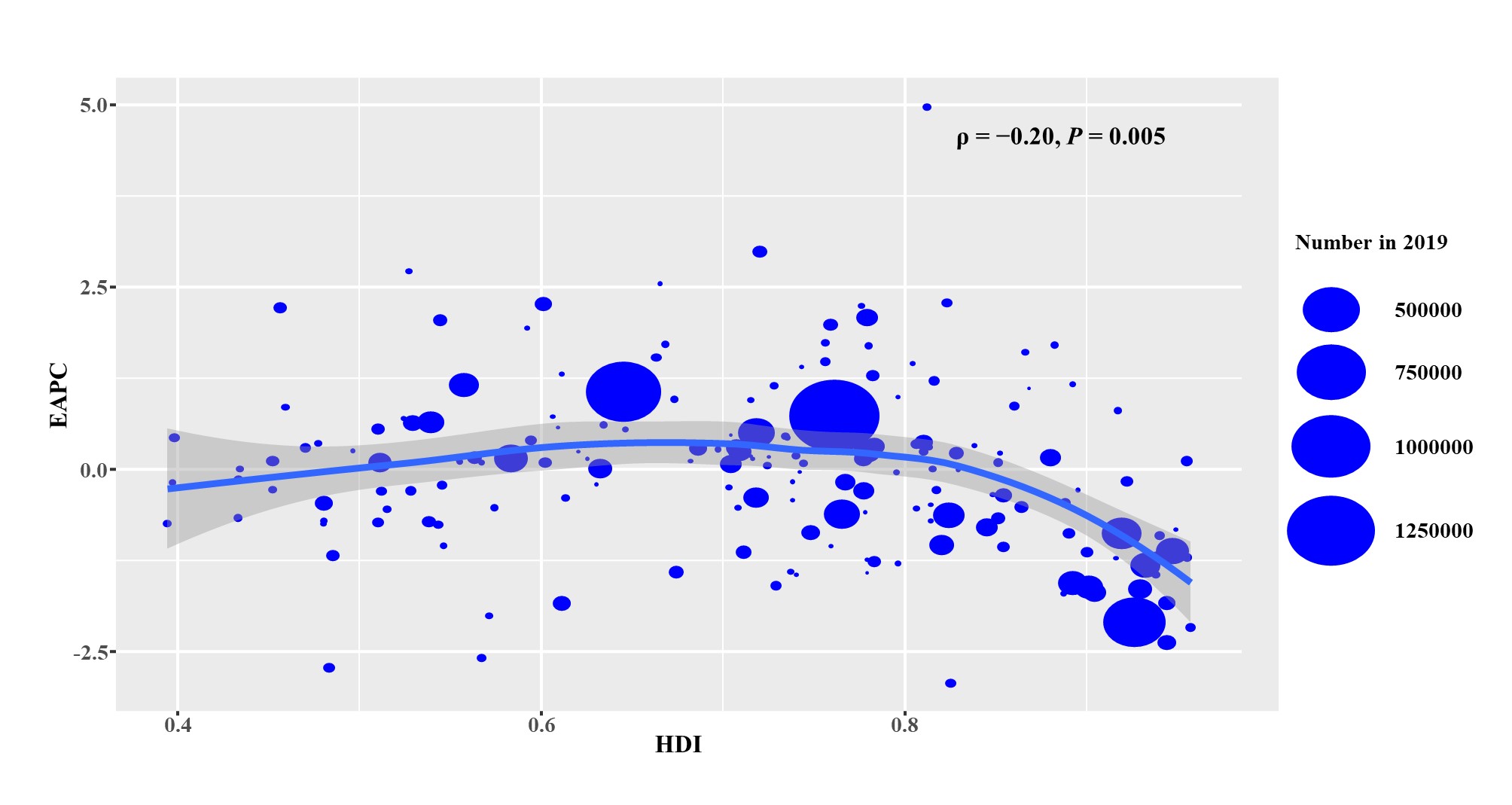
**

**Supplementary table 1**. the incident number of non-Hodgkin lymphoma in 2019, and the percentage changes in number during the period 1990-2019 in age groups

| **Age Groups** | **Incidence** | | **Death** | | **DALYs** | |
| --- | --- | --- | --- | --- | --- | --- |
|  | Number  ×10^3^(95% UI) | Change in number (%) | Number  ×10^3^(95% UI) | Change in number (%) | Number  ×10^3^(95% UI) | Change in number (%) |
| **< 5** | 0(0-0) | 0 | 1.92(1.51-2.37) | −45.87 | 165.13(130.26-203.8) | −45.88 |
| **5 to 9** | 7.88(6.24-10.27) | 1.03 | 2.07(1.77-2.39) | −22.1 | 173.26(148.49-200.51) | −21.61 |
| **10 to 14** | 6.91(5.81-8.47) | 22.35 | 1.84(1.63-2.05) | −3.29 | 144.03(128.08-161.25) | −2.69 |
| **15 to 19** | 10.4(9.06-12.19) | 31.27 | 2.83(2.56-3.11) | 4.04 | 207.75(188.46-227.98) | 4.73 |
| **20 to 24** | 8.34(7.26-9.74) | 62.35 | 3.67(3.36-4.03) | 30.01 | 248.47(227.19-272.69) | 30.57 |
| **25 to 29** | 9.23(8.22-10.41) | 72.94 | 3.97(3.68-4.33) | 39.38 | 249.61(231.07-272.33) | 40.13 |
| **30 to 34** | 11.2(10.07-12.51) | 90.83 | 4.67(4.35-5.07) | 55.09 | 270.68(252-293.26) | 56.01 |
| **35 to 39** | 13.25(11.9-14.76) | 79.81 | 5.65(5.23-6.13) | 49.09 | 299.44(277.6-325.66) | 50.04 |
| **40 to 44** | 12.2(10.98-13.5) | 83.29 | 7.48(6.92-8.11) | 60.94 | 355.52(328.81-385.34) | 61.55 |
| **45 to 49** | 16.96(15.09-18.94) | 117.66 | 10.17(9.35-11.04) | 86.25 | 434.7(400.02-471.29) | 87.22 |
| **50 to 54** | 25.66(22.9-28.7) | 134.28 | 15.14(13.93-16.47) | 93.64 | 576.3(529.97-625.31) | 95.13 |
| **55 to 59** | 34.02(30.35-37.97) | 132.26 | 19.6(18.25-21.09) | 89.54 | 655.79(610.43-704.87) | 91.42 |
| **60 to 64** | 41.45(36.94-46.29) | 125.07 | 24.61(23.14-26.18) | 86.77 | 709.54(666.15-755.4) | 88.74 |
| **65 to 69** | 52.22(46.56-58.47) | 139.69 | 30(28.3-31.65) | 99.87 | 733.13(689.93-774.93) | 102.35 |
| **70 to 74** | 59.24(52.71-66.44) | 177.55 | 32.4(30.52-34.16) | 125.4 | 659(621.85-698.81) | 129.76 |
| **75 to 79** | 56.77(49.95-63.63) | 156.83 | 29.8(27.69-31.38) | 109.93 | 490.96(453.68-521.16) | 115.13 |
| **> 80** | 91.33(74.86-105.05) | 316.36 | 58.77(49.43-63.82) | 231.10 | 618.02(527.07-672.85) | 226.13 |

SDI: socio-demographic index;

**Supplementary table 2**. the number and age-standardized rate of non-Hodgkin lymphoma incidence at national level and both sexes in 1990 and 2019, and percentage changes in number and the EAPCs from 1990 to 2019

|  | **1990** | | **2019** | | **1990-2019** | |
| --- | --- | --- | --- | --- | --- | --- |
| **Characteristics** | Number  ×10^3^ (95% UI) | ASR/100,000  (95% UI) | Number  ×10^3^ (95% UI) | ASR/100,000  (95% UI) | Percentage changes (%) | EAPCs  (95%CI) |
| Afghanistan | 0.93(0.57-1.26) | 12.46(7.41-17.4) | 1.97(1.22-3.1) | 13.36(7.49-22.6) | 112.66 | 0.37(0.22-0.51) |
| Albania | 0.06(0.05-0.07) | 2.22(1.98-2.46) | 0.13(0.09-0.16) | 3.43(2.61-4.41) | 114.08 | 1.76(1.6-1.93) |
| Algeria | 0.92(0.59-1.31) | 5.27(3.61-7.28) | 2.15(1.73-2.62) | 6.31(5.11-7.69) | 134.25 | 0.68(0.56-0.81) |
| American Samoa | 0(0-0) | 2.47(1.92-2.97) | 0(0-0) | 3.46(2.82-4.31) | 132.3 | 1.62(1.36-1.89) |
| Andorra | 0.01(0.01-0.01) | 14.06(10.67-18.76) | 0.02(0.01-0.02) | 14.86(11.39-18.97) | 155.23 | 0.12(0.02-0.21) |
| Angola | 0.13(0.1-0.19) | 2.51(1.82-3.3) | 0.47(0.33-0.63) | 2.97(2.14-3.87) | 252.82 | 0.76(0.61-0.91) |
| Antigua and Barbuda | 0(0-0) | 4.8(4.25-5.44) | 0.01(0-0.01) | 5.46(4.47-6.62) | 98.64 | 0.37(0.2-0.54) |
| Argentina | 1.61(1.47-1.75) | 4.93(4.52-5.35) | 3.33(2.58-4.28) | 6.41(4.95-8.21) | 107.61 | 0.41(0.1-0.71) |
| Armenia | 0.05(0.04-0.05) | 1.53(1.34-1.78) | 0.12(0.09-0.14) | 3.15(2.55-3.82) | 148.92 | 3.24(2.76-3.72) |
| Australia | 2.57(2.38-2.78) | 13.29(12.3-14.34) | 4.7(3.6-6.03) | 11.33(8.68-14.5) | 82.63 | −1.21(−1.47-−0.94) |
| Austria | 0.77(0.71-0.84) | 6.83(6.32-7.35) | 1.58(1.28-1.96) | 9.05(7.33-11.16) | 104.21 | 0.97(0.66-1.29) |
| Azerbaijan | 0.09(0.07-0.11) | 1.37(1.16-1.66) | 0.26(0.21-0.34) | 2.72(2.17-3.51) | 204.66 | 2.9(2.5-3.29) |
| Bahamas | 0.01(0.01-0.01) | 5.54(4.88-6.35) | 0.02(0.02-0.03) | 5.73(4.56-7.16) | 103.79 | 0.29(0.2-0.38) |
| Bahrain | 0.01(0.01-0.01) | 4.26(3.55-5.09) | 0.07(0.05-0.08) | 7.11(5.65-8.71) | 549.05 | 2.54(2.08-3.01) |
| Bangladesh | 1.01(0.7-1.42) | 1.47(1.14-1.89) | 2.67(1.86-3.8) | 1.86(1.32-2.61) | 165.14 | 0.85(0.73-0.98) |
| Barbados | 0.02(0.02-0.03) | 8.45(7.42-9.57) | 0.04(0.03-0.05) | 9.61(7.77-11.88) | 71.87 | 0.29(0.2-0.38) |
| Belarus | 0.25(0.22-0.28) | 2.15(1.91-2.46) | 0.67(0.52-0.88) | 5.06(3.88-6.58) | 175.03 | 3.96(3.47-4.44) |
| Belgium | 1.22(1.13-1.33) | 8.42(7.81-9.1) | 2.16(1.66-2.76) | 9.65(7.39-12.5) | 76.42 | 0.06(−0.18-0.29) |
| Belize | 0(0-0) | 1.81(1.58-2.07) | 0.01(0.01-0.01) | 2.6(2.16-3.09) | 269.12 | 0.97(0.71-1.23) |
| Benin | 0.07(0.05-0.09) | 2.43(2.03-2.9) | 0.2(0.13-0.28) | 2.63(1.91-3.52) | 187.19 | −0.06(−0.27-0.16) |
| Bermuda | 0.01(0.01-0.01) | 10.6(9.43-11.78) | 0.01(0.01-0.01) | 8.66(7.15-10.67) | 40.74 | −0.68(−0.83-−0.53) |
| Bhutan | 0(0-0.01) | 1.38(0.94-1.95) | 0.02(0.01-0.02) | 2.68(1.89-3.57) | 250.93 | 2.35(2.31-2.39) |
| Bolivia | 0.13(0.1-0.16) | 3.41(2.75-4.1) | 0.48(0.37-0.61) | 5.23(4.07-6.56) | 270.49 | 1.37(1.29-1.44) |
| Bosnia and Herzegovina | 0.09(0.08-0.1) | 2.02(1.82-2.25) | 0.24(0.19-0.3) | 4.76(3.77-5.98) | 167.43 | 3.76(3.33-4.2) |
| Botswana | 0.01(0.01-0.02) | 1.74(1.31-2.29) | 0.05(0.03-0.07) | 2.78(2-3.78) | 286.96 | 0.98(0.61-1.35) |
| Brazil | 3.45(3.23-3.73) | 3.07(2.89-3.28) | 9.04(8.38-9.75) | 3.92(3.64-4.23) | 162.06 | 0.96(0.78-1.15) |
| Brunei Darussalam | 0.01(0.01-0.02) | 10.07(7.67-12.8) | 0.04(0.04-0.05) | 13.13(11.37-15.22) | 187.47 | 1.48(1.25-1.72) |
| Bulgaria | 0.36(0.33-0.39) | 3.42(3.11-3.73) | 0.62(0.48-0.79) | 5.61(4.36-7.15) | 71.52 | 2.16(1.91-2.41) |
| Burkina Faso | 0.12(0.09-0.16) | 2.13(1.68-2.65) | 0.35(0.25-0.47) | 2.56(1.97-3.24) | 183.95 | 0.22(0-0.44) |
| Burundi | 0.07(0.05-0.1) | 2.61(2.01-3.41) | 0.14(0.09-0.2) | 2.43(1.64-3.36) | 90.98 | −0.38(−0.47-−0.3) |
| Cabo Verde | 0(0-0) | 1.28(1.13-1.45) | 0.02(0.02-0.03) | 4.45(3.71-5.27) | 523.29 | 3.74(3.25-4.23) |
| Cambodia | 0.13(0.09-0.18) | 2.06(1.63-2.61) | 0.38(0.3-0.46) | 2.84(2.29-3.41) | 193.55 | 1.14(1.01-1.26) |
| Cameroon | 0.23(0.14-0.37) | 3.28(2.24-4.77) | 0.82(0.42-1.57) | 3.8(2.19-6.71) | 258.82 | 0.43(0.39-0.48) |
| Canada | 4.1(3.75-4.49) | 12.86(11.78-14.06) | 8.61(6.4-11.6) | 12.97(9.72-17.46) | 110.08 | −0.44(−0.66-−0.21) |
| Central African Republic | 0.04(0.03-0.05) | 2.47(1.96-3.08) | 0.07(0.04-0.09) | 2.29(1.47-3.27) | 80.36 | −0.15(−0.22-−0.08) |
| Chad | 0.07(0.05-0.09) | 1.96(1.49-2.42) | 0.23(0.16-0.31) | 2.52(1.91-3.22) | 215.68 | 0.63(0.39-0.86) |
| Chile | 0.52(0.47-0.57) | 4.74(4.31-5.19) | 1.52(1.2-1.94) | 6.65(5.27-8.5) | 192.01 | 1.19(0.89-1.49) |
| China | 20.08(17.05-24.08) | 2.04(1.76-2.4) | 91.95(76.98-108.97) | 4.99(4.24-5.87) | 357.93 | 3.72(3.42-4.02) |
| Colombia | 0.79(0.73-0.87) | 3.38(3.12-3.69) | 2.52(1.95-3.27) | 4.94(3.84-6.4) | 217.39 | 1.5(1.39-1.63) |
| Comoros | 0.01(0-0.01) | 2.44(1.38-3.38) | 0.01(0.01-0.02) | 2.77(1.98-3.72) | 135.18 | 0.33(0.16-0.51) |
| Congo | 0.04(0.03-0.06) | 3.35(2.59-4.3) | 0.1(0.08-0.14) | 3.2(2.41-4.13) | 133.56 | −0.16(−0.32-0) |
| Cook Islands | 0(0-0) | 2.48(2.06-3.05) | 0(0-0) | 3.24(2.55-4.01) | 103.04 | 1.06(0.86-1.25) |
| Costa Rica | 0.12(0.11-0.14) | 5.79(5.21-6.46) | 0.4(0.31-0.52) | 7.97(6.15-10.3) | 227.75 | 1.39(1.06-1.72) |
| Croatia | 0.36(0.32-0.4) | 6.00(5.45-6.64) | 0.69(0.53-0.88) | 8.74(6.75-11.01) | 93.90 | 1.57(1.24-1.91) |
| Cuba | 0.75(0.69-0.82) | 7.04(6.47-7.61) | 1.1(0.88-1.38) | 6.76(5.42-8.42) | 46.29 | −0.04(−0.44-0.35) |
| Cyprus | 0.07(0.06-0.09) | 9.30(7.75-11) | 0.19(0.17-0.23) | 10.37(8.92-12.04) | 162.45 | 0.09(−0.22-0.4) |
| Czechia | 0.84(0.77-0.91) | 6.49(5.97-7) | 1.46(1.18-1.85) | 7.42(5.99-9.29) | 74.37 | 0.41(0.21-0.62) |
| Côte d'Ivoire | 0.20(0.13-0.29) | 3.06(2.19-4.12) | 0.48(0.32-0.67) | 2.98(2.17-3.92) | 142.42 | −0.62(−0.89-−0.35) |
| Democratic People's Republic of Korea | 0.47(0.34-0.63) | 2.56(1.95-3.33) | 0.79(0.55-1.07) | 2.58(1.78-3.45) | 68.74 | 0.18(−0.06-0.43) |
| Democratic Republic of the Congo | 0.41(0.31-0.53) | 2.05(1.52-2.74) | 0.94(0.57-1.41) | 2(1.13-3.14) | 130.66 | −0.09(−0.32-0.14) |
| Denmark | 0.50(0.45-0.55) | 6.58(5.92-7.36) | 1.01(0.76-1.34) | 9.2(6.89-12.1) | 103.48 | 0.53(0.07-0.99) |
| Djibouti | 0(0-0.01) | 2.72(1.74-3.94) | 0.02(0.02-0.03) | 3.46(2.48-4.78) | 370.21 | 0.88(0.81-0.96) |
| Dominica | 0.01(0.01-0.01) | 8.53(7.4-9.83) | 0.01(0.01-0.01) | 9.11(7.23-11.47) | 20.43 | 0.39(0.3-0.48) |
| Dominican Republic | 0.09(0.08-0.11) | 1.75(1.5-2.04) | 0.34(0.23-0.46) | 3.38(2.36-4.58) | 264.98 | 2.61(2.43-2.79) |
| Ecuador | 0.17(0.16-0.19) | 2.53(2.37-2.7) | 1.00(0.79-1.27) | 6.34(5.02-8.06) | 478.70 | 3.7(3.35-4.04) |
| Egypt | 0.70(0.61-0.8) | 1.64(1.48-1.8) | 2.01(1.53-2.61) | 2.49(1.89-3.31) | 188.03 | 1.45(1.31-1.6) |
| El Salvador | 0.06(0.05-0.06) | 1.55(1.44-1.67) | 0.22(0.17-0.29) | 3.64(2.77-4.72) | 277.12 | 2.75(2.36-3.13) |
| Equatorial Guinea | 0.01(0-0.01) | 2.15(1.58-2.8) | 0.03(0.02-0.05) | 3.96(2.53-6.03) | 446.61 | 2.9(2.63-3.18) |
| Eritrea | 0.03(0.02-0.03) | 2.09(1.59-2.72) | 0.1(0.07-0.14) | 3.07(2.26-4.02) | 286.1 | 1.13(0.87-1.4) |
| Estonia | 0.05(0.05-0.06) | 3.04(2.7-3.39) | 0.18(0.14-0.23) | 7.75(6.01-9.68) | 229.41 | 3.49(3.04-3.93) |
| Eswatini | 0.01(0.01-0.01) | 2.93(2.32-3.6) | 0.03(0.02-0.05) | 4.12(2.76-5.83) | 172.5 | 1.29(0.83-1.74) |
| Ethiopia | 0.27(0.21-0.38) | 1.11(0.87-1.51) | 0.48(0.35-0.69) | 0.95(0.68-1.37) | 76.9 | −0.72(−0.94-−0.5) |
| Fiji | 0.01(0.01-0.01) | 1.81(1.46-2.2) | 0.02(0.02-0.03) | 2.55(2-3.18) | 127.14 | 1.47(1.27-1.68) |
| Finland | 0.7(0.64-0.76) | 10.29(9.42-11.26) | 1.34(1-1.81) | 11.41(8.52-15.23) | 92.21 | 0.21(0.03-0.39) |
| France | 7.25(6.67-7.82) | 9.27(8.55-9.95) | 13.54(10.1-17.78) | 10.04(7.57-13.09) | 86.68 | −0.14(−0.34-0.06) |
| Gabon | 0.03(0.02-0.04) | 4(2.52-6.17) | 0.05(0.04-0.07) | 4.24(3.16-5.57) | 100.69 | 0.11(0.04-0.18) |
| Gambia | 0(0-0) | 0.66(0.47-0.88) | 0.01(0.01-0.02) | 0.83(0.6-1.12) | 227.29 | 0.57(0.39-0.76) |
| Georgia | 0.09(0.08-0.11) | 1.57(1.34-1.83) | 0.22(0.18-0.27) | 4.42(3.58-5.51) | 134.41 | 4.70(4.20-5.21) |
| Germany | 9.07(8.46-9.72) | 7.76(7.26-8.29) | 17.64(13.63-22.99) | 9.51(7.29-12.3) | 94.45 | 0.26(0.04-0.48) |
| Ghana | 0.67(0.47-0.96) | 5.86(4.5-7.62) | 1.14(0.81-1.51) | 5.08(3.82-6.47) | 70.24 | −1.22(−1.61-−0.83) |
| Greece | 0.71(0.66-0.78) | 5.14(4.74-5.61) | 1.23(0.95-1.55) | 5.96(4.62-7.57) | 72.43 | 0.23(0-0.46) |
| Greenland | 0(0-0) | 6.89(5.94-8.03) | 0(0-0.01) | 6.42(5.19-7.69) | 56.85 | −0.74(−0.99-−0.48) |
| Grenada | 0.01(0.01-0.01) | 12.46(10.87-14.27) | 0.01(0.01-0.01) | 11.21(9.52-13.13) | 33.08 | −0.34(−0.51-−0.17) |
| Guam | 0.01(0-0.01) | 5.41(4.62-6.36) | 0.01(0.01-0.01) | 5.06(4.17-6.13) | 78.38 | −0.44(−0.97-0.09) |
| Guatemala | 0.07(0.06-0.08) | 1.37(1.18-1.6) | 0.3(0.23-0.38) | 2.23(1.72-2.83) | 338.81 | 2.2(1.88-2.52) |
| Guinea | 0.03(0.02-0.04) | 0.69(0.48-0.96) | 0.07(0.04-0.11) | 0.81(0.53-1.25) | 133.95 | 0.64(0.61-0.67) |
| Guinea-Bissau | 0.02(0.01-0.03) | 3.27(2.1-4.39) | 0.03(0.02-0.04) | 3.11(2.38-4.14) | 78.53 | −0.47(−0.65-−0.29) |
| Guyana | 0.02(0.01-0.02) | 3.01(2.54-3.5) | 0.02(0.02-0.03) | 3.23(2.39-4.28) | 42.07 | 0.33(0.21-0.45) |
| Haiti | 0.17(0.12-0.23) | 3.96(2.98-4.88) | 0.31(0.22-0.42) | 3.44(2.31-4.61) | 81.11 | −0.3(−0.49-−0.11) |
| Honduras | 0.05(0.04-0.05) | 1.58(1.32-1.86) | 0.16(0.12-0.22) | 2.4(1.86-3.37) | 254.28 | 1.58(1.5-1.66) |
| Hungary | 0.73(0.67-0.79) | 5.44(5.04-5.89) | 1.18(0.96-1.46) | 6.76(5.5-8.31) | 61.14 | 0.32(0.08-0.56) |
| Iceland | 0.02(0.02-0.03) | 8.12(6.98-9.43) | 0.04(0.04-0.06) | 8.53(6.79-10.66) | 94.29 | −0.01(−0.16-0.14) |
| India | 9.23(7.44-12.36) | 1.61(1.37-1.97) | 32.46(26.85-40.4) | 2.66(2.21-3.26) | 251.77 | 1.72(1.65-1.8) |
| Indonesia | 2.49(1.96-3.02) | 1.86(1.55-2.19) | 6.48(5.04-8.27) | 2.85(2.23-3.58) | 160.62 | 1.37(1.32-1.43) |
| Iran  (Islamic Republic of) | 0.93(0.75-1.15) | 2.4(2-2.79) | 3.03(2.78-3.27) | 3.96(3.64-4.28) | 225.69 | 1.59(1.44-1.74) |
| Iraq | 0.43(0.3-0.61) | 3.59(2.59-4.85) | 1.06(0.81-1.33) | 3.57(2.79-4.39) | 143.64 | 0.07(−0.13-0.27) |
| Ireland | 0.38(0.35-0.41) | 9.38(8.63-10.21) | 0.87(0.66-1.11) | 12(9.19-15.32) | 128.8 | 0.51(0.19-0.83) |
| Israel | 0.46(0.42-0.51) | 9.57(8.7-10.54) | 1.51(1.16-1.92) | 13.2(10.12-16.91) | 225.65 | 0.5(0.12-0.89) |
| Italy | 8.14(7.63-8.63) | 10.05(9.48-10.62) | 14.29(11.58-16.94) | 10.8(8.87-12.81) | 75.44 | −0.26(−0.6-0.07) |
| Jamaica | 0.09(0.08-0.1) | 4.56(4.02-5.21) | 0.23(0.17-0.29) | 7.68(5.85-9.85) | 152.32 | 1.21(0.54-1.89) |
| Japan | 10.91(10.23-11.57) | 6.83(6.43-7.23) | 30.25(24.59-35.92) | 8.32(6.99-9.78) | 177.24 | 0.62(0.45-0.78) |
| Jordan | 0.12(0.09-0.15) | 6.35(4.98-8.04) | 0.57(0.48-0.69) | 7.46(6.26-8.91) | 388.05 | 0.36(0.21-0.51) |
| Kazakhstan | 0.37(0.31-0.44) | 2.43(2.1-2.88) | 0.46(0.38-0.54) | 2.58(2.18-3.04) | 24.09 | −1.46(−2.03-−0.89) |
| Kenya | 0.22(0.17-0.27) | 2.06(1.53-2.51) | 0.91(0.7-1.21) | 3.34(2.62-4.4) | 310.18 | 2.05(1.84-2.27) |
| Kiribati | 0(0-0) | 2.62(2.11-3.17) | 0(0-0) | 2.58(1.99-3.31) | 77.48 | −0.11(−0.15-−0.07) |
| Kuwait | 0.05(0.05-0.06) | 6.07(5.24-7.12) | 0.18(0.15-0.23) | 6.82(5.47-8.43) | 245.73 | 1.05(0.68-1.42) |
| Kyrgyzstan | 0.05(0.04-0.06) | 1.38(1.18-1.62) | 0.1(0.08-0.11) | 1.85(1.56-2.19) | 90.41 | 1.3(1.16-1.45) |
| Lao People's Democratic Republic | 0.07(0.04-0.1) | 2.37(1.57-3.35) | 0.14(0.1-0.19) | 2.63(1.93-3.5) | 113.16 | 0.25(0.16-0.33) |
| Latvia | 0.08(0.07-0.09) | 2.68(2.37-3.02) | 0.2(0.16-0.24) | 6.1(4.99-7.39) | 143.71 | 3.36(2.78-3.96) |
| Lebanon | 0.09(0.07-0.11) | 3.53(2.73-4.46) | 0.32(0.26-0.44) | 6.3(4.97-8.53) | 266.17 | 2.4(2.24-2.55) |
| Lesotho | 0.02(0.02-0.03) | 1.96(1.38-2.68) | 0.05(0.04-0.07) | 3.54(2.52-4.76) | 140 | 2.59(2.37-2.81) |
| Liberia | 0.03(0.03-0.05) | 2.43(1.9-3.1) | 0.07(0.04-0.11) | 2.31(1.29-3.39) | 105.36 | −0.18(−0.37-0) |
| Libya | 0.07(0.05-0.1) | 2.93(2.11-3.96) | 0.22(0.16-0.31) | 3.76(2.66-5.41) | 191.69 | 1.3(1.03-1.58) |
| Lithuania | 0.1(0.09-0.12) | 2.56(2.28-2.85) | 0.27(0.22-0.33) | 5.46(4.4-6.7) | 156.62 | 3.09(2.56-3.62) |
| Luxembourg | 0.04(0.04-0.05) | 8.23(7.42-9.11) | 0.09(0.07-0.11) | 9.09(7.27-11.3) | 103.92 | 0.02(−0.35-0.4) |
| Madagascar | 0.15(0.11-0.2) | 2.24(1.79-2.74) | 0.3(0.23-0.39) | 2.24(1.69-2.94) | 97.41 | 0.03(−0.03-0.08) |
| Malawi | 0.35(0.21-0.58) | 5.43(3.85-7.85) | 0.38(0.28-0.53) | 4.24(3.39-5.33) | 9.11 | −1.17(−1.37-−0.97) |
| Malaysia | 0.44(0.4-0.49) | 3.86(3.5-4.28) | 1.97(1.53-2.45) | 7.04(5.5-8.72) | 348.41 | 2.04(1.9-2.17) |
| Maldives | 0(0-0) | 2.68(1.76-3.52) | 0.01(0.01-0.02) | 3.73(3.09-4.45) | 307.91 | 1.14(0.89-1.39) |
| Mali | 0.03(0.03-0.04) | 0.61(0.49-0.74) | 0.09(0.06-0.13) | 0.68(0.48-0.99) | 173.09 | 0.31(0.24-0.38) |
| Malta | 0.03(0.03-0.03) | 7.23(6.23-8.31) | 0.08(0.06-0.1) | 9.35(7.52-11.58) | 155.23 | 0.72(0.51-0.93) |
| Marshall Islands | 0(0-0) | 2.35(1.91-2.8) | 0(0-0) | 2.74(2.04-3.51) | 110.58 | 0.58(0.52-0.65) |
| Mauritania | 0.04(0.03-0.05) | 2.92(2.28-3.69) | 0.07(0.05-0.11) | 2.6(1.78-3.62) | 92.13 | −0.61(−0.74-−0.47) |
| Mauritius | 0.01(0.01-0.02) | 1.65(1.46-1.86) | 0.05(0.04-0.06) | 3.09(2.44-3.87) | 219.4 | 2.08(1.87-2.28) |
| Mexico | 1.5(1.39-1.63) | 2.51(2.37-2.69) | 5.07(4.36-5.86) | 4.24(3.66-4.9) | 238.63 | 1.71(1.43-1.98) |
| Micronesia  (Federated States of) | 0(0-0) | 2.48(1.93-3.14) | 0(0-0) | 3(1.88-4.29) | 59.39 | 0.63(0.57-0.68) |
| Monaco | 0.01(0.01-0.02) | 22.46(18.08-27.12) | 0.04(0.03-0.04) | 42.02(33.49-51.8) | 160.12 | 2.53(2.06-3) |
| Mongolia | 0.04(0.03-0.05) | 2.95(2.45-3.47) | 0.08(0.06-0.1) | 3.02(2.36-3.95) | 111.15 | −0.55(−0.82-−0.27) |
| Montenegro | 0.03(0.02-0.03) | 4.13(3.47-4.77) | 0.05(0.04-0.05) | 5.22(4.37-6.2) | 77.01 | 1.2(1.04-1.36) |
| Morocco | 0.55(0.43-0.67) | 3.33(2.54-3.97) | 1.63(1.24-2.05) | 5.15(3.92-6.4) | 196.56 | 1.26(1.06-1.46) |
| Mozambique | 0.14(0.12-0.17) | 2.44(2.04-2.86) | 0.48(0.36-0.62) | 4.03(3.08-5.21) | 232.01 | 2.18(2.02-2.35) |
| Myanmar | 2.53(1.84-3.44) | 9.73(6.98-12.89) | 5.53(4.55-6.8) | 11.92(9.97-14.63) | 118.75 | 0.7(0.63-0.76) |
| Namibia | 0.02(0.02-0.03) | 2.74(1.98-3.44) | 0.06(0.04-0.09) | 3.66(2.55-5.02) | 173.3 | 0.84(0.64-1.04) |
| Nauru | 0(0-0) | 4.13(2.7-6.14) | 0(0-0) | 4.56(3.19-5.94) | 22.61 | 0.05(−0.22-0.33) |
| Nepal | 0.19(0.12-0.27) | 1.49(1.05-1.98) | 0.52(0.39-0.67) | 2.1(1.62-2.67) | 172.58 | 1.27(1.02-1.52) |
| Netherlands | 2.45(2.24-2.65) | 12.72(11.67-13.74) | 4.08(3.13-5.26) | 12.38(9.44-15.88) | 66.61 | −0.55(−0.75-−0.35) |
| New Zealand | 0.44(0.4-0.49) | 11.28(10.27-12.43) | 0.84(0.68-1.05) | 11.22(9.09-14.02) | 91.64 | −0.67(−1.01-−0.33) |
| Nicaragua | 0.05(0.04-0.06) | 1.75(1.51-2.04) | 0.18(0.14-0.22) | 3.6(2.92-4.37) | 287.06 | 3(2.77-3.23) |
| Niger | 0.04(0.03-0.06) | 0.8(0.59-1.05) | 0.11(0.06-0.18) | 0.79(0.5-1.24) | 186.06 | −0.18(−0.3-−0.06) |
| Nigeria | 0.84(0.56-1.27) | 1.36(1-1.87) | 2.52(1.76-3.61) | 1.84(1.37-2.41) | 198.91 | 1.36(1.18-1.55) |
| Niue | 0(0-0) | 2.94(2.32-3.74) | 0(0-0) | 4.14(3.14-5.57) | 26.44 | 1.21(1.14-1.28) |
| North Macedonia | 0.05(0.05-0.06) | 2.74(2.44-3.07) | 0.12(0.09-0.15) | 4.22(3.34-5.27) | 123.66 | 1.45(1.29-1.62) |
| Northern Mariana Islands | 0(0-0) | 3.23(2.5-4.23) | 0(0-0) | 5.46(4.52-6.61) | 175.2 | 2.16(1.95-2.37) |
| Norway | 0.72(0.67-0.77) | 11.3(10.58-12.01) | 0.92(0.76-1.07) | 9.78(8.08-11.43) | 27.36 | −0.78(−1.05-−0.52) |
| Oman | 0.05(0.03-0.06) | 6.06(3.9-8.03) | 0.21(0.16-0.26) | 10.95(8.77-13.25) | 324.59 | 2.28(1.95-2.61) |
| Pakistan | 0.93(0.66-1.22) | 1.31(0.96-1.68) | 3.26(2.18-4.63) | 2.05(1.41-2.87) | 249.75 | 1.48(1.3-1.67) |
| Palau | 0(0-0) | 2.21(1.64-2.9) | 0(0-0) | 2.8(2.2-3.53) | 116.61 | 0.71(0.64-0.77) |
| Palestine | 0.04(0.03-0.05) | 3.55(2.64-4.75) | 0.14(0.11-0.16) | 4.67(3.95-5.57) | 235.04 | 0.69(0.38-0.99) |
| Panama | 0.06(0.05-0.07) | 3.42(3.01-3.85) | 0.2(0.15-0.26) | 4.78(3.65-6.3) | 230.07 | 0.99(0.9-1.08) |
| Papua New Guinea | 0.02(0.01-0.02) | 0.79(0.63-0.98) | 0.05(0.04-0.06) | 0.86(0.67-1.1) | 172.93 | 0.23(0.19-0.28) |
| Paraguay | 0.06(0.05-0.06) | 1.97(1.74-2.22) | 0.23(0.18-0.3) | 3.89(2.95-5.01) | 312.12 | 2.37(2.24-2.49) |
| Peru | 0.55(0.48-0.65) | 3.7(3.19-4.3) | 2.1(1.57-2.79) | 6.45(4.83-8.55) | 279.73 | 2.16(1.97-2.35) |
| Philippines | 1.2(1.08-1.32) | 2.82(2.53-3.11) | 2.72(2.25-3.31) | 2.98(2.48-3.63) | 127.66 | 0.01(−0.23-0.24) |
| Poland | 1.44(1.33-1.55) | 3.42(3.17-3.67) | 4.21(3.54-4.96) | 6.71(5.66-7.9) | 192.87 | 2.24(1.8-2.68) |
| Portugal | 0.75(0.69-0.81) | 5.95(5.51-6.39) | 2.1(1.62-2.67) | 9.78(7.54-12.61) | 180.47 | 1.42(1.05-1.79) |
| Puerto Rico | 0.26(0.24-0.28) | 7.16(6.55-7.81) | 0.46(0.35-0.6) | 7.45(5.74-9.66) | 76.17 | 0.1(−0.08-0.28) |
| Qatar | 0.01(0.01-0.01) | 6.2(4.89-7.67) | 0.1(0.07-0.14) | 11.04(8.17-14.61) | 965.24 | 2.57(2.22-2.92) |
| Republic of Korea | 1.16(1.06-1.27) | 3.09(2.85-3.36) | 5.46(4.49-6.54) | 6.7(5.54-8) | 369.48 | 1.94(1.49-2.39) |
| Republic of Moldova | 0.12(0.1-0.13) | 2.65(2.33-3.03) | 0.2(0.17-0.24) | 4.2(3.54-4.94) | 68.34 | 1.71(1.46-1.96) |
| Romania | 0.78(0.71-0.87) | 3.11(2.82-3.46) | 1.53(1.23-1.89) | 5.31(4.31-6.55) | 95.8 | 2.08(1.9-2.25) |
| Russian Federation | 5.49(5.1-5.94) | 3.33(3.09-3.6) | 8.72(7.58-10.02) | 4.26(3.71-4.89) | 58.82 | 1.11(0.79-1.43) |
| Rwanda | 0.1(0.07-0.14) | 2.9(2.16-3.75) | 0.21(0.15-0.29) | 2.99(2.33-3.97) | 106 | −0.28(−0.53-−0.03) |
| Saint Kitts and Nevis | 0(0-0) | 8.26(7.12-9.47) | 0(0-0.01) | 7.35(5.58-9.32) | 50.04 | −0.18(−0.4-0.03) |
| Saint Lucia | 0.01(0.01-0.01) | 7.43(6.68-8.25) | 0.02(0.01-0.02) | 7.66(6.16-9.32) | 100.97 | −0.09(−0.29-0.11) |
| Saint Vincent and the Grenadines | 0.01(0.01-0.01) | 8.24(7.08-9.49) | 0.01(0.01-0.01) | 8.82(7.25-10.52) | 54.61 | 0.22(0.13-0.32) |
| Samoa | 0(0-0) | 2.41(1.67-3.25) | 0(0-0.01) | 2.91(1.95-3.99) | 82.82 | 0.59(0.5-0.67) |
| San Marino | 0.01(0.01-0.01) | 20.78(17.78-24.2) | 0.01(0.01-0.02) | 23.79(18.08-32.23) | 121.16 | 0.7(0.61-0.78) |
| Sao Tome and Principe | 0(0-0) | 2.24(1.71-2.77) | 0(0-0.01) | 3.1(2.23-4.15) | 118.45 | 0.84(0.63-1.04) |
| Saudi Arabia | 0.31(0.16-0.43) | 4.25(2.22-5.65) | 1.77(1.36-2.27) | 8.33(6.62-10.33) | 462.17 | 2.19(2.07-2.31) |
| Senegal | 0.12(0.09-0.15) | 2.56(1.98-3.1) | 0.28(0.19-0.39) | 2.79(2.03-3.72) | 142.41 | 0.13(−0.01-0.26) |
| Serbia | 0.41(0.34-0.48) | 3.83(3.19-4.53) | 0.9(0.71-1.13) | 6.34(5.02-7.93) | 121.78 | 2.23(2.01-2.45) |
| Seychelles | 0(0-0) | 3.99(3.53-4.49) | 0.01(0.01-0.01) | 8.33(7.19-9.58) | 270.78 | 2.29(2.03-2.54) |
| Sierra Leone | 0.05(0.04-0.07) | 2.21(1.67-2.83) | 0.13(0.09-0.18) | 2.38(1.66-3.21) | 133.7 | 0.06(−0.06-0.18) |
| Singapore | 0.14(0.13-0.17) | 5.92(5.17-6.86) | 0.5(0.38-0.65) | 6.9(5.33-8.92) | 244.07 | 0.37(0.13-0.61) |
| Slovakia | 0.27(0.24-0.31) | 4.62(4.07-5.29) | 0.7(0.55-0.9) | 8.19(6.46-10.39) | 160. | 2.41(2.24-2.59) |
| Slovenia | 0.13(0.1-0.16) | 5.4(4.1-6.96) | 0.44(0.33-0.58) | 10.58(8.07-13.75) | 249.03 | 2.64(2.42-2.87) |
| Solomon Islands | 0.01(0.01-0.02) | 6.7(5.06-8.49) | 0.03(0.03-0.04) | 7.7(6.19-9.55) | 157.76 | 0.34(0.21-0.46) |
| Somalia | 0.06(0.04-0.08) | 1.87(1.31-2.57) | 0.16(0.09-0.25) | 1.89(0.98-2.96) | 194.79 | 0.2(0.13-0.28) |
| South Africa | 0.91(0.81-1.01) | 3.35(2.97-3.7) | 2.04(1.77-2.33) | 4.02(3.52-4.55) | 123.21 | 0.74(0.55-0.92) |
| South Sudan | 0.09(0.04-0.16) | 3.21(1.65-5.64) | 0.14(0.08-0.23) | 3.17(1.86-4.98) | 62.03 | 0.07(−0.03-0.17) |
| Spain | 3.96(3.67-4.23) | 8.02(7.48-8.54) | 7.89(6.05-10.12) | 8.87(6.81-11.28) | 99.14 | −0.12(−0.47-0.22) |
| Sri Lanka | 0.25(0.22-0.29) | 1.92(1.69-2.18) | 1.01(0.74-1.33) | 4.14(3.08-5.41) | 304.5 | 3.2(2.95-3.45) |
| Sudan | 0.2(0.14-0.28) | 1.64(1.19-2.27) | 0.62(0.37-0.93) | 2.32(1.43-3.46) | 216.29 | 1.3(1.09-1.5) |
| Suriname | 0.01(0.01-0.01) | 3.98(3.31-4.44) | 0.03(0.02-0.03) | 4.59(3.67-5.59) | 116.62 | 0.48(0.18-0.79) |
| Sweden | 1.55(1.42-1.69) | 10.8(9.89-11.71) | 2.13(1.75-2.54) | 9.96(8.18-11.84) | 37.62 | −0.78(−1.01-−0.55) |
| Switzerland | 0.45(0.4-0.5) | 4.69(4.21-5.18) | 1.59(1.21-2.09) | 9.27(7.05-12.19) | 250.78 | 1.36(0.39-2.34) |
| Syrian Arab Republic | 0.18(0.14-0.24) | 2.49(1.93-3.14) | 0.32(0.23-0.43) | 2.48(1.8-3.32) | 76.12 | −0.81(−1.36-−0.26) |
| Taiwan  (Province of China) | 1.06(0.97-1.17) | 6.02(5.48-6.62) | 3.19(2.41-4.2) | 8.85(6.71-11.65) | 199.54 | 1.15(0.92-1.38) |
| Tajikistan | 0.05(0.04-0.07) | 1.38(1.16-1.68) | 0.17(0.13-0.22) | 2.44(1.96-3.07) | 217.06 | 2.14(1.8-2.47) |
| Thailand | 0.79(0.69-0.92) | 1.78(1.55-2.05) | 2.93(2.19-3.85) | 3.2(2.43-4.19) | 270.84 | 1.64(1.47-1.8) |
| Timor-Leste | 0.01(0-0.01) | 1.6(1.21-2.04) | 0.02(0.02-0.03) | 2.31(1.67-2.93) | 234.3 | 1.51(1.23-1.8) |
| Togo | 0.05(0.04-0.07) | 2.48(2.05-3.01) | 0.14(0.09-0.19) | 2.58(1.79-3.49) | 169.9 | −0.31(−0.52-−0.11) |
| Tokelau | 0(0-0) | 1.98(1.51-2.58) | 0(0-0) | 2.86(2.04-3.92) | 35.53 | 1.33(1.25-1.42) |
| Tonga | 0(0-0) | 4.25(3.5-5.11) | 0(0-0.01) | 5.36(4.14-6.98) | 66.13 | 0.65(0.54-0.77) |
| Trinidad and Tobago | 0.05(0.05-0.06) | 5.19(4.61-5.78) | 0.08(0.06-0.11) | 4.81(3.38-6.55) | 47.26 | −0.38(−0.59-−0.17) |
| Tunisia | 0.08(0.07-0.1) | 1.34(1.11-1.58) | 0.29(0.22-0.39) | 2.34(1.74-3.12) | 259.41 | 1.92(1.85-1.99) |
| Turkey | 1.82(1.38-2.32) | 4.06(3.13-5.1) | 5.09(4.09-6.22) | 5.92(4.79-7.21) | 178.77 | 1.76(1.45-2.06) |
| Turkmenistan | 0.03(0.03-0.05) | 1.31(1.09-1.62) | 0.1(0.07-0.13) | 2.23(1.71-2.88) | 183.13 | 2.08(1.78-2.38) |
| Tuvalu | 0(0-0) | 2.2(1.65-2.8) | 0(0-0) | 2.61(1.77-3.59) | 69.61 | 0.46(0.43-0.5) |
| Uganda | 0.11(0.08-0.13) | 1.39(1.12-1.69) | 0.49(0.36-0.65) | 2.32(1.83-2.86) | 358.36 | 1.97(1.86-2.09) |
| Ukraine | 1.52(1.38-1.69) | 2.6(2.33-2.91) | 3.14(2.57-3.75) | 5.47(4.5-6.54) | 105.92 | 3.07(2.68-3.47) |
| United Arab Emirates | 0.06(0.03-0.1) | 6.42(2.67-11.08) | 0.51(0.27-0.78) | 6.91(3.5-10.55) | 782.81 | 0.05(−0.32-0.42) |
| United Kingdom | 8.76(8.22-9.29) | 10.38(9.8-10.94) | 14.01(11.67-16.67) | 11.44(9.53-13.63) | 60.01 | −0.07(−0.26-0.12) |
| United Republic of Tanzania | 0.43(0.34-0.54) | 3.73(3.04-4.5) | 1.21(0.99-1.51) | 4.49(3.76-5.32) | 179.85 | 0.71(0.59-0.82) |
| United States of America | 41.5(38.87-43.95) | 13.26(12.46-14) | 60.84(52.26-70.34) | 11.08(9.51-12.81) | 46.58 | −1.3(−1.54-−1.06) |
| United States Virgin Islands | 0.01(0-0.01) | 6.06(4.98-7.28) | 0.01(0.01-0.02) | 7.59(6.11-9.39) | 117.6 | 0.8(0.59-1.01) |
| Uruguay | 0.25(0.23-0.27) | 6.85(6.24-7.5) | 0.45(0.35-0.58) | 9.3(7.21-11.93) | 81.71 | 0.87(0.61-1.12) |
| Uzbekistan | 0.23(0.2-0.29) | 1.43(1.26-1.71) | 0.96(0.77-1.18) | 3.49(2.85-4.24) | 308.64 | 3.88(3.54-4.22) |
| Vanuatu | 0(0-0) | 1.74(1.28-2.29) | 0(0-0.01) | 2.18(1.52-2.93) | 209.9 | 0.59(0.46-0.72) |
| Venezuela | 0.54(0.48-0.6) | 3.97(3.59-4.42) | 1.35(0.99-1.81) | 4.66(3.46-6.22) | 151.25 | 0.36(0.23-0.48) |
| Viet Nam | 0.9(0.71-1.16) | 1.92(1.52-2.43) | 2.84(2.23-3.55) | 3.05(2.41-3.78) | 214.4 | 1.71(1.54-1.87) |
| Yemen | 0.11(0.07-0.16) | 1.67(1.2-2.28) | 0.34(0.23-0.47) | 1.85(1.25-2.52) | 211.64 | 0.72(0.6-0.85) |
| Zambia | 0.13(0.1-0.16) | 4.17(3.36-5.04) | 0.37(0.29-0.47) | 4.78(3.89-5.86) | 185.2 | 0.38(0.35-0.41) |
| Zimbabwe | 0.11(0.09-0.13) | 1.96(1.66-2.26) | 0.11(0.08-0.15) | 1.31(1.01-1.67) | 4.72 | −2(−2.31-−1.69) |

EAPC: estimated annual percentage change; ASR, age-standardized rate; CI, confidence interval; UI: uncertainty interval.

**Supplementary table 3.** The number and age-standardized rate of death caused by non-Hodgkin lymphoma in global, sexes, SDI areas and geographic regions in 1990 and 2019, and percentage change of absolute number and the EAPCs from 1990 to 2019

|  | **1990** | | **2019** | | **1990-2019** | |
| --- | --- | --- | --- | --- | --- | --- |
| **Characteristics** | Number  ×10^3^ (95% UI) | ASR/100,000  (95% UI) | Number  ×10^3^ (95% UI) | ASR/100,000  (95% UI) | Percentage change (%) | EAPC  (95%CI) |
| **Overall** | 126.08  (119.77-131.98) | 3.15  (3.00-3.29) | 254.61  (237.71-270.35) | 3.19  (2.98-3.39) | 101.94 | −0.09  (−0.17-−0.02) |
| **Sex** |  |  |  |  |  |  |
| Male | 70.77  (67.01-75.21) | 3.84  (3.65-4.04) | 145.62  (135.74-155.43) | 3.97  (3.71-4.23) | 105.77 | 0.08  (0.01-0.15) |
| Female | 55.32  (51.76-58.68) | 2.58(  2.41-2.73) | 109.00  (98.91-117.14) | 2.52  (2.29-2.71) | 97.05 | −0.34  (−0.44-−0.23) |
| **SDI** |  |  |  |  |  |  |
| Low | 6.63  (5.41-7.85) | 2.34  (2.04-2.65) | 14.80  (12.72-17.09) | 2.51  (2.12-2.93) | 123.21 | 0.26  (0.21-0.30) |
| Low-middle | 15.09  (13.12-17.46) | 2.18  (1.95-2.46) | 38.24  (34.67-42.42) | 2.74  (2.49-3.03) | 153.31 | 0.83  (0.81-0.86) |
| Middle | 23.99  (22.13-25.7) | 2.05  (1.91-2.18) | 62.23  (56.93-68.34) | 2.55  (2.33-2.81) | 159.39 | 0.94  (0.84-1.03) |
| High-middle | 27.84  (26.64-28.92) | 2.61  (2.49-2.71) | 54.72  (50.52-58.45) | 2.81  (2.60-3.00) | 96.53 | 0.19  (0.08-0.29) |
| High | 52.44  (50.26-53.64) | 5.09  (4.88-5.20) | 84.47  (76.27-89.26) | 4.33  (3.97-4.55) | 61.09 | −0.98  (−1.15-−0.82) |
| **Regions** |  |  |  |  |  |  |
| East Asia | 17.42  (15.44-19.55) | 1.86  (1.66-2.08) | 46.45  (39.66-54) | 2.35  (2.02-2.71) | 166.58 | 1.16  (0.95-1.36) |
| South Asia | 10.51  (8.93-12.27) | 1.60  (1.41-1.84) | 32.62  (28.05-37.95) | 2.28  (1.97-2.66) | 210.46 | 1.25  (1.18-1.32) |
| Southeast Asia | 8.45  (7.32-9.78) | 2.89  (2.58-3.28) | 19.89  (17.32-22.88) | 3.31  (2.91-3.79) | 135.36 | 0.44  (0.40-0.47) |
| Central Asia | 0.71  (0.68-0.74) | 1.34  (1.28-1.40) | 1.51  (1.35-1.71) | 1.92  (1.71-2.16) | 113.06 | 1.06  (0.89-1.23) |
| High-income  Asia Pacific | 6.49  (6.23-6.65) | 3.34  (3.19-3.43) | 16.28  (13.79-17.64) | 3.34  (2.94-3.57) | 150.72 | −0.18  (−0.33-−0.04) |
| Oceania | 0.06  (0.05-0.07) | 1.65  (1.42-1.88) | 0.13  (0.11-0.15) | 1.70  (1.45-2.00) | 126.61 | 0.10  (0.03-0.16) |
| Australasia | 1.49  (1.41-1.57) | 6.41  (6.03-6.73) | 2.33  (2.04-2.58) | 4.57  (4.04-5.05) | 56.14 | −1.78  (−2.01-−1.56) |
| Eastern Europe | 4.93  (4.76-5.12) | 1.89  (1.82-1.96) | 7.18  (6.45-7.93) | 2.29  (2.06-2.53) | 45.59 | 0.75  (0.52-0.97) |
| Western Europe | 25.58  (24.59-26.21) | 4.57  (4.40-4.68) | 38.26  (34.53-40.64) | 4.07  (3.74-4.29) | 49.62 | −0.81  (−0.99-−0.63) |
| Central Europe | 3.77  (3.65-3.91) | 2.67  (2.58-2.76) | 6.32  (5.57-7.14) | 3.13  (2.77-3.54) | 67.40 | 0.58  (0.42-0.75) |
| High-income North America | 23.74  (22.64-24.35) | 6.75  (6.46-6.92) | 33.58  (31-35.37) | 5.27  (4.90-5.53) | 41.48 | −1.46  (−1.67-−1.26) |
| Andean Latin America | 0.83  (0.74-0.92) | 3.51  (3.16-3.90) | 2.64  (2.16-3.21) | 4.68  (3.84-5.69) | 218.58 | 1.14  (0.96-1.32) |
| Central Latin America | 2.67  (2.58-2.75) | 2.64  (2.54-2.73) | 6.79  (5.9-7.86) | 2.86  (2.49-3.32) | 154.65 | 0.28  (0.22-0.34) |
| Caribbean | 1.21  (1.12-1.29) | 4.26  (3.98-4.51) | 1.86  (1.58-2.16) | 3.65  (3.11-4.25) | 53.66 | −0.47  (−0.66-−0.28) |
| Tropical Latin America | 3.04  (2.93-3.14) | 2.93  (2.80-3.03) | 6.50  (6.07-6.86) | 2.73  (2.55-2.89) | 113.60 | −0.18  (−0.31-−0.05) |
| Southern Latin America | 1.95  (1.88-2.03) | 4.21  (4.03-4.36) | 3.33  (3.07-3.61) | 4.06  (3.76-4.4) | 70.21 | −0.43  (−0.69-−0.17) |
| Eastern Sub-  Saharan Africa | 2.40  (1.89-2.91) | 2.79  (2.41-3.20) | 5.50  (4.66-6.53) | 3.19  (2.76-3.69) | 129.03 | 0.57  (0.47-0.66) |
| Southern Sub-  Saharan Africa | 1.01  (0.91-1.09) | 3.07  (2.73-3.38) | 2.04  (1.83-2.28) | 3.34  (3.01-3.71) | 102.22 | 0.41  (0.20-0.62) |
| Western Sub-  Saharan Africa | 2.47  (1.95-3.09) | 2.04  (1.71-2.43) | 5.55  (4.54-6.6) | 2.24  (1.88-2.62) | 124.56 | 0.20  (0.10-0.30) |
| North Africa and Middle East | 6.59  (5.53-7.64) | 3.34  (2.83-3.79) | 14.26  (12.44-16.24) | 3.17  (2.75-3.6) | 116.26 | −0.18  (−0.31-−0.05) |
| Central Sub-  Saharan Africa | 0.76  (0.60-0.96) | 2.58  (2.05-3.10) | 1.61  (1.15-2.17) | 2.51  (1.73-3.58) | 111.59 | −0.05  (−0.22-0.12) |

EAPC: estimated annual percentage change; ASR, age-standardized rate; CI, confidence interval; UI: uncertainty interval; SDI: socio-demographic index.

**Supplementary table 4**. the number and age-standardized rate of death caused by non-Hodgkin lymphoma at national level and both sexes in 1990 and 2019, and percentage changes in number and the EAPCs from 1990 to 2019

|  | **1990** | | **2019** | | **1990-2019** | |
| --- | --- | --- | --- | --- | --- | --- |
| **Characteristics** | Number  ×10^3^(95% UI) | ASR/100,000  (95% UI) | Number  ×10^3^(95% UI) | ASR/100,000  (95% UI) | Percentage changes (%) | EAPC  (95%CI) |
| Afghanistan | 1(0.62-1.35) | 13.75(8.09-19.36) | 2(1.23-3.12) | 14.84(8.2-25.28) | 100.54 | 0.39(0.25-0.53) |
| Albania | 0.04(0.04-0.04) | 1.74(1.6-1.89) | 0.06(0.05-0.08) | 1.63(1.23-2.09) | 55.59 | 0.01(−0.22-0.24) |
| Algeria | 0.69(0.47-0.96) | 5.08(3.63-6.91) | 1.32(1.07-1.59) | 4.14(3.35-4.96) | 91.26 | −0.63(−0.74-−0.53) |
| American Samoa | 0(0-0) | 2.43(1.94-2.9) | 0(0-0) | 2.92(2.4-3.55) | 119.17 | 1.04(0.73-1.35) |
| Andorra | 0(0-0) | 6.78(5.14-9.15) | 0.01(0.01-0.01) | 5.66(4.37-7.13) | 118.9 | −0.67(−0.72-−0.62) |
| Angola | 0.16(0.11-0.24) | 2.88(2.12-3.76) | 0.45(0.33-0.59) | 3.18(2.32-4.11) | 180.57 | 0.51(0.4-0.63) |
| Antigua and Barbuda | 0(0-0) | 3.98(3.57-4.45) | 0(0-0) | 3.55(2.89-4.25) | 59.93 | −0.43(−0.66-−0.2) |
| Argentina | 1.36(1.3-1.42) | 4.22(4.02-4.41) | 2.2(1.98-2.46) | 4.15(3.75-4.62) | 61.93 | −0.46(−0.73-−0.18) |
| Armenia | 0.03(0.03-0.04) | 1.17(1.09-1.27) | 0.07(0.06-0.08) | 1.74(1.44-2.09) | 101.83 | 1.93(1.54-2.33) |
| Australia | 1.27(1.19-1.34) | 6.55(6.14-6.92) | 1.97(1.7-2.21) | 4.57(3.98-5.09) | 55.53 | −1.86(−2.08-−1.63) |
| Austria | 0.42(0.39-0.45) | 3.56(3.36-3.77) | 0.69(0.61-0.76) | 3.71(3.32-4.09) | 63.83 | 0.23(0-0.47) |
| Azerbaijan | 0.06(0.06-0.07) | 1.11(1.01-1.23) | 0.17(0.13-0.21) | 1.8(1.42-2.25) | 164.57 | 2.14(1.83-2.45) |
| Bahamas | 0.01(0.01-0.01) | 4.72(4.21-5.32) | 0.02(0.01-0.02) | 3.95(3.15-4.96) | 81.65 | −0.48(−0.61-−0.36) |
| Bahrain | 0.01(0.01-0.01) | 4.15(3.5-4.89) | 0.03(0.03-0.04) | 4.1(3.28-5.04) | 319.75 | 0.55(0.12-0.98) |
| Bangladesh | 0.93(0.72-1.18) | 1.51(1.24-1.83) | 2.14(1.57-2.9) | 1.61(1.19-2.15) | 129.69 | 0.3(0.15-0.45) |
| Barbados | 0.02(0.02-0.02) | 6.53(5.84-7.26) | 0.03(0.02-0.03) | 5.87(4.76-7.2) | 43.5 | −0.49(−0.57-−0.41) |
| Belarus | 0.15(0.14-0.16) | 1.27(1.17-1.36) | 0.33(0.26-0.43) | 2.3(1.79-2.97) | 119.61 | 2.91(2.38-3.44) |
| Belgium | 0.67(0.63-0.72) | 4.47(4.2-4.74) | 0.95(0.83-1.07) | 3.98(3.52-4.48) | 41.42 | −0.8(−1.01-−0.59) |
| Belize | 0(0-0) | 1.71(1.54-1.92) | 0.01(0.01-0.01) | 1.98(1.67-2.35) | 207.73 | 0.28(0.01-0.56) |
| Benin | 0.07(0.06-0.09) | 2.62(2.22-3.09) | 0.18(0.13-0.25) | 2.7(2.01-3.54) | 149.08 | −0.21(−0.43-0) |
| Bermuda | 0(0-0.01) | 7.46(6.72-8.18) | 0(0-0.01) | 3.89(3.22-4.75) | −1.09 | −2.21(−2.46-−1.96) |
| Bhutan | 0(0-0.01) | 1.47(1.01-2.05) | 0.01(0.01-0.02) | 2.39(1.72-3.15) | 190.16 | 1.76(1.7-1.82) |
| Bolivia | 0.14(0.11-0.17) | 3.81(3.08-4.55) | 0.47(0.37-0.58) | 5.34(4.28-6.54) | 233.52 | 1.13(1.06-1.2) |
| Bosnia and Herzegovina | 0.07(0.06-0.07) | 1.54(1.43-1.66) | 0.13(0.11-0.17) | 2.46(1.95-3.07) | 104.76 | 2.07(1.74-2.39) |
| Botswana | 0.01(0.01-0.02) | 1.82(1.39-2.39) | 0.04(0.03-0.05) | 2.42(1.78-3.22) | 221.84 | 0.43(0.03-0.83) |
| Brazil | 2.99(2.89-3.09) | 2.95(2.82-3.06) | 6.33(5.92-6.69) | 2.73(2.55-2.89) | 111.63 | −0.21(−0.35-−0.08) |
| Brunei Darussalam | 0.01(0.01-0.01) | 8.24(6.38-10.27) | 0.02(0.02-0.03) | 8.41(7.36-9.6) | 147.18 | 0.73(0.46-1.01) |
| Bulgaria | 0.23(0.22-0.25) | 2.08(1.93-2.24) | 0.35(0.28-0.45) | 2.89(2.25-3.68) | 51.7 | 1.56(1.33-1.79) |
| Burkina Faso | 0.13(0.1-0.18) | 2.36(1.88-2.91) | 0.33(0.25-0.43) | 2.67(2.13-3.29) | 149.98 | 0.04(−0.19-0.26) |
| Burundi | 0.08(0.06-0.11) | 3(2.34-3.92) | 0.14(0.1-0.2) | 2.73(1.86-3.8) | 66.86 | −0.47(−0.56-−0.38) |
| Cabo Verde | 0(0-0) | 1.28(1.14-1.42) | 0.02(0.01-0.02) | 3.3(2.83-3.85) | 367.44 | 2.64(2.12-3.15) |
| Cambodia | 0.13(0.09-0.17) | 2.19(1.76-2.76) | 0.35(0.28-0.42) | 2.78(2.25-3.28) | 166.02 | 0.88(0.77-0.99) |
| Cameroon | 0.19(0.14-0.24) | 3.16(2.24-4.19) | 0.54(0.34-0.86) | 3.3(2.07-5.26) | 191.36 | 0.08(0.02-0.13) |
| Canada | 2.03(1.89-2.17) | 6.34(5.88-6.79) | 3.59(3-4.28) | 5.16(4.34-6.1) | 76.57 | −1.2(−1.41-−0.98) |
| Central African Republic | 0.04(0.03-0.05) | 2.76(2.21-3.43) | 0.07(0.05-0.1) | 2.53(1.63-3.65) | 71.56 | −0.2(−0.28-−0.13) |
| Chad | 0.08(0.06-0.1) | 2.14(1.68-2.56) | 0.21(0.16-0.28) | 2.64(2.05-3.32) | 169.35 | 0.47(0.24-0.71) |
| Chile | 0.4(0.38-0.42) | 3.86(3.63-4.09) | 0.83(0.75-0.91) | 3.56(3.24-3.89) | 108.65 | −0.36(−0.6-−0.13) |
| China | 16.44(14.4-18.59) | 1.82(1.61-2.05) | 44.31(37.46-51.97) | 2.32(1.97-2.7) | 169.6 | 1.2(0.99-1.42) |
| Colombia | 0.65(0.61-0.68) | 3.11(2.93-3.32) | 1.51(1.17-1.94) | 2.91(2.25-3.73) | 134.23 | −0.04(−0.2-0.13) |
| Comoros | 0.01(0-0.01) | 2.83(1.69-3.8) | 0.02(0.01-0.02) | 3.1(2.24-4.11) | 123.19 | 0.25(0.11-0.38) |
| Congo | 0.05(0.04-0.06) | 3.72(2.89-4.71) | 0.1(0.07-0.13) | 3.43(2.61-4.4) | 110.9 | −0.31(−0.46-−0.15) |
| Cook Islands | 0(0-0) | 1.93(1.63-2.27) | 0(0-0) | 1.93(1.58-2.36) | 73.49 | 0.16(−0.04-0.35) |
| Costa Rica | 0.08(0.07-0.09) | 4.26(3.87-4.69) | 0.23(0.17-0.29) | 4.46(3.43-5.7) | 175.84 | 0.35(0.04-0.67) |
| Croatia | 0.2(0.19-0.22) | 3.33(3.05-3.64) | 0.32(0.25-0.4) | 3.76(2.9-4.7) | 57.85 | 0.66(0.33-0.99) |
| Cuba | 0.5(0.47-0.53) | 4.73(4.47-4.98) | 0.63(0.5-0.77) | 3.58(2.88-4.42) | 25.46 | −0.84(−1.23-−0.45) |
| Cyprus | 0.05(0.04-0.06) | 6.39(5.31-7.57) | 0.08(0.07-0.09) | 4.36(3.78-4.97) | 68.08 | −1.79(−1.98-−1.6) |
| Czechia | 0.53(0.49-0.56) | 3.95(3.67-4.21) | 0.69(0.55-0.84) | 3.31(2.65-4.06) | 30.14 | −0.55(−0.68-−0.42) |
| Côte d'Ivoire | 0.19(0.13-0.27) | 3.23(2.36-4.32) | 0.43(0.3-0.58) | 3.04(2.27-3.97) | 127.46 | −0.73(−1.01-−0.46) |
| Democratic People's Republic of Korea | 0.38(0.29-0.49) | 2.12(1.68-2.65) | 0.64(0.44-0.84) | 2.02(1.41-2.64) | 69.6 | −0.02(−0.1-0.07) |
| Democratic Republic of the Congo | 0.48(0.38-0.62) | 2.34(1.79-3.05) | 0.92(0.55-1.37) | 2.16(1.21-3.4) | 91.55 | −0.31(−0.54-−0.08) |
| Denmark | 0.28(0.25-0.3) | 3.48(3.17-3.81) | 0.44(0.36-0.52) | 3.77(3.12-4.48) | 58.25 | −0.44(−0.87-0) |
| Djibouti | 0.01(0-0.01) | 3.13(2.04-4.48) | 0.02(0.02-0.03) | 3.88(2.84-5.23) | 341.08 | 0.79(0.71-0.86) |
| Dominica | 0.01(0-0.01) | 7.49(6.62-8.38) | 0.01(0.01-0.01) | 7.25(5.89-8.91) | 15.23 | −0.01(−0.13-0.1) |
| Dominican Republic | 0.08(0.07-0.09) | 1.73(1.49-1.98) | 0.25(0.18-0.34) | 2.6(1.86-3.49) | 205.71 | 1.86(1.62-2.1) |
| Ecuador | 0.16(0.15-0.17) | 2.65(2.48-2.82) | 0.72(0.58-0.93) | 4.79(3.82-6.11) | 340.92 | 2.49(2.2-2.78) |
| Egypt | 0.6(0.54-0.66) | 1.57(1.44-1.7) | 1.32(1.01-1.74) | 1.81(1.36-2.4) | 120.77 | 0.62(0.55-0.68) |
| El Salvador | 0.05(0.05-0.06) | 1.57(1.46-1.67) | 0.16(0.12-0.2) | 2.56(1.96-3.3) | 184.51 | 1.33(0.97-1.68) |
| Equatorial Guinea | 0.01(0-0.01) | 2.41(1.8-3.1) | 0.02(0.01-0.04) | 4.07(2.62-6.07) | 299.65 | 2.55(2.29-2.81) |
| Eritrea | 0.03(0.02-0.04) | 2.4(1.84-3.17) | 0.1(0.07-0.14) | 3.47(2.56-4.54) | 244.19 | 1.08(0.82-1.33) |
| Estonia | 0.04(0.03-0.04) | 1.89(1.72-2.07) | 0.09(0.07-0.11) | 3.54(2.77-4.43) | 150.44 | 2(1.5-2.5) |
| Eswatini | 0.01(0.01-0.01) | 3.14(2.48-3.85) | 0.03(0.02-0.04) | 4.17(2.84-5.81) | 152.87 | 1.1(0.64-1.57) |
| Ethiopia | 0.3(0.22-0.42) | 1.23(0.97-1.68) | 0.49(0.35-0.69) | 1.05(0.75-1.5) | 62 | −0.72(−0.93-−0.5) |
| Fiji | 0.01(0.01-0.01) | 1.84(1.48-2.23) | 0.02(0.01-0.02) | 2.44(1.93-3.04) | 130.7 | 1.31(1.1-1.51) |
| Finland | 0.39(0.36-0.41) | 5.51(5.13-5.87) | 0.57(0.48-0.68) | 4.55(3.8-5.46) | 47.84 | −0.86(−0.98-−0.74) |
| France | 4.11(3.85-4.37) | 5.04(4.73-5.35) | 5.97(4.8-7.24) | 4.09(3.38-4.86) | 45.34 | −1.17(−1.34-−1) |
| Gabon | 0.03(0.02-0.04) | 4.36(2.74-6.67) | 0.05(0.04-0.06) | 4.35(3.33-5.56) | 78.69 | −0.07(−0.15-0) |
| Gambia | 0(0-0) | 0.71(0.51-0.94) | 0.01(0.01-0.01) | 0.84(0.62-1.13) | 188.4 | 0.39(0.22-0.56) |
| Georgia | 0.07(0.06-0.08) | 1.11(0.98-1.25) | 0.15(0.12-0.18) | 2.82(2.3-3.39) | 125.69 | 4.54(4.01-5.07) |
| Germany | 5.05(4.79-5.28) | 4.11(3.92-4.29) | 7.89(7-8.83) | 3.95(3.55-4.36) | 56.29 | −0.46(−0.66-−0.27) |
| Ghana | 0.54(0.43-0.69) | 5.55(4.42-6.77) | 0.95(0.72-1.2) | 4.86(3.79-6.04) | 73.89 | −0.96(−1.25-−0.67) |
| Greece | 0.35(0.33-0.38) | 2.43(2.27-2.6) | 0.55(0.48-0.61) | 2.38(2.13-2.62) | 55.54 | −0.38(−0.69-−0.07) |
| Greenland | 0(0-0) | 6.18(5.48-6.98) | 0(0-0) | 4.7(3.83-5.61) | 39.68 | −1.42(−1.65-−1.2) |
| Grenada | 0.01(0.01-0.01) | 11.84(10.4-13.43) | 0.01(0.01-0.01) | 8.37(7.23-9.58) | 6.63 | −1.06(−1.22-−0.91) |
| Guam | 0(0-0) | 4.19(3.61-4.89) | 0.01(0.01-0.01) | 3.4(2.82-4.07) | 81.14 | −0.77(−1.49-−0.04) |
| Guatemala | 0.06(0.06-0.07) | 1.43(1.25-1.62) | 0.24(0.19-0.31) | 1.99(1.58-2.5) | 277.78 | 1.55(1.26-1.83) |
| Guinea | 0.03(0.02-0.04) | 0.74(0.53-1) | 0.06(0.04-0.09) | 0.82(0.55-1.24) | 94.96 | 0.44(0.41-0.47) |
| Guinea-Bissau | 0.02(0.01-0.03) | 3.48(2.25-4.64) | 0.03(0.02-0.04) | 3.18(2.41-4.21) | 56.65 | −0.62(−0.8-−0.44) |
| Guyana | 0.01(0.01-0.02) | 2.99(2.55-3.46) | 0.02(0.01-0.03) | 2.85(2.16-3.74) | 35.13 | −0.02(−0.12-0.09) |
| Haiti | 0.2(0.13-0.27) | 4.4(3.2-5.41) | 0.31(0.21-0.41) | 3.59(2.44-4.91) | 56.23 | −0.5(−0.69-−0.31) |
| Honduras | 0.04(0.04-0.05) | 1.65(1.37-1.95) | 0.15(0.11-0.2) | 2.34(1.87-3.21) | 238.57 | 1.45(1.33-1.57) |
| Hungary | 0.5(0.47-0.53) | 3.62(3.42-3.83) | 0.61(0.5-0.74) | 3.27(2.66-3.97) | 20.59 | −0.74(−0.91-−0.57) |
| Iceland | 0.01(0.01-0.01) | 3.66(3.24-4.17) | 0.02(0.01-0.02) | 3.08(2.47-3.74) | 61.23 | −0.77(−0.92-−0.61) |
| India | 8.38(7.24-9.75) | 1.65(1.46-1.89) | 27.26(22.9-32.47) | 2.38(2.01-2.83) | 225.43 | 1.29(1.21-1.37) |
| Indonesia | 2.35(1.86-2.83) | 1.93(1.61-2.27) | 5.84(4.55-7.22) | 2.72(2.14-3.32) | 148.45 | 1.16(1.1-1.22) |
| Iran  (Islamic Republic of) | 0.61(0.52-0.7) | 1.93(1.63-2.16) | 1.58(1.47-1.69) | 2.13(1.98-2.29) | 158.26 | 0.35(0.18-0.52) |
| Iraq | 0.33(0.24-0.45) | 3.35(2.46-4.42) | 0.64(0.5-0.8) | 2.46(1.94-3.01) | 92.1 | −1.12(−1.33-−0.91) |
| Ireland | 0.21(0.19-0.22) | 5.07(4.74-5.43) | 0.34(0.3-0.38) | 4.57(4.05-5.14) | 65.44 | −0.79(−1.05-−0.54) |
| Israel | 0.29(0.27-0.31) | 6.07(5.55-6.6) | 0.7(0.62-0.79) | 5.98(5.29-6.71) | 143.35 | −0.65(−0.98-−0.33) |
| Italy | 4.01(3.87-4.11) | 4.74(4.58-4.85) | 5.9(5.28-6.3) | 4.07(3.72-4.32) | 47.11 | −1.02(−1.28-−0.76) |
| Jamaica | 0.07(0.06-0.08) | 3.77(3.36-4.2) | 0.16(0.12-0.2) | 5.27(4.06-6.75) | 123.78 | 0.59(−0.02-1.2) |
| Japan | 5.67(5.43-5.81) | 3.47(3.32-3.56) | 13.72(11.46-15.02) | 3.44(3.02-3.69) | 141.9 | −0.14(−0.26-−0.02) |
| Jordan | 0.1(0.08-0.12) | 6.35(5.05-7.95) | 0.31(0.26-0.37) | 4.52(3.85-5.35) | 220.48 | −1.57(−1.77-−1.36) |
| Kazakhstan | 0.24(0.22-0.26) | 1.72(1.6-1.84) | 0.26(0.22-0.31) | 1.49(1.27-1.73) | 7.64 | −2.16(−2.73-−1.58) |
| Kenya | 0.23(0.18-0.26) | 2.3(1.71-2.79) | 0.9(0.7-1.19) | 3.82(2.97-5.02) | 297.37 | 2.27(2.01-2.53) |
| Kiribati | 0(0-0) | 2.81(2.27-3.41) | 0(0-0) | 2.68(2.12-3.38) | 67.23 | −0.19(−0.23-−0.16) |
| Kuwait | 0.03(0.03-0.03) | 4.11(3.58-4.82) | 0.08(0.07-0.1) | 3.38(2.73-4.17) | 179.43 | −0.08(−0.48-0.31) |
| Kyrgyzstan | 0.04(0.04-0.04) | 1.16(1.07-1.27) | 0.06(0.05-0.07) | 1.31(1.12-1.54) | 62.59 | 0.6(0.38-0.81) |
| Lao People's Democratic Republic | 0.07(0.04-0.1) | 2.54(1.69-3.54) | 0.13(0.09-0.17) | 2.67(1.98-3.52) | 90.39 | 0.05(−0.03-0.12) |
| Latvia | 0.05(0.05-0.06) | 1.64(1.49-1.82) | 0.11(0.09-0.13) | 3.07(2.54-3.76) | 107.17 | 2.42(1.8-3.03) |
| Lebanon | 0.07(0.06-0.09) | 3.15(2.53-3.86) | 0.16(0.12-0.21) | 3.02(2.39-4.06) | 113.4 | 0.08(−0.06-0.21) |
| Lesotho | 0.02(0.02-0.03) | 2.1(1.49-2.84) | 0.05(0.04-0.07) | 3.72(2.68-4.97) | 134.03 | 2.51(2.29-2.73) |
| Liberia | 0.04(0.03-0.05) | 2.71(2.13-3.46) | 0.06(0.03-0.09) | 2.34(1.3-3.41) | 61.71 | −0.47(−0.66-−0.28) |
| Libya | 0.06(0.04-0.08) | 2.74(2.01-3.63) | 0.14(0.1-0.2) | 2.58(1.83-3.69) | 133.89 | 0.07(−0.08-0.22) |
| Lithuania | 0.07(0.06-0.07) | 1.54(1.4-1.68) | 0.16(0.13-0.19) | 2.91(2.37-3.56) | 140.08 | 2.49(1.95-3.03) |
| Luxembourg | 0.02(0.02-0.03) | 4.4(4.03-4.83) | 0.04(0.03-0.04) | 3.61(3.02-4.34) | 54.12 | −0.96(−1.2-−0.72) |
| Madagascar | 0.16(0.13-0.2) | 2.53(2.03-3.04) | 0.3(0.23-0.39) | 2.52(1.89-3.34) | 85.86 | 0.01(−0.04-0.06) |
| Malawi | 0.35(0.21-0.52) | 5.83(4.18-7.68) | 0.35(0.28-0.42) | 4.71(3.92-5.55) | −1.45 | −1.01(−1.15-−0.87) |
| Malaysia | 0.39(0.36-0.43) | 3.85(3.49-4.23) | 1.26(0.99-1.56) | 4.71(3.74-5.78) | 222.33 | 0.54(0.4-0.67) |
| Maldives | 0(0-0) | 2.82(1.88-3.71) | 0.01(0.01-0.01) | 2.21(1.84-2.62) | 133.48 | −1.23(−1.43-−1.04) |
| Mali | 0.04(0.03-0.04) | 0.66(0.55-0.79) | 0.08(0.06-0.11) | 0.7(0.51-0.98) | 131.91 | 0.16(0.09-0.24) |
| Malta | 0.02(0.02-0.02) | 4.1(3.6-4.63) | 0.03(0.03-0.04) | 3.7(3.07-4.39) | 91.67 | −0.55(−0.72-−0.39) |
| Marshall Islands | 0(0-0) | 2.5(2.04-2.98) | 0(0-0) | 2.79(2.1-3.55) | 102.25 | 0.44(0.37-0.52) |
| Mauritania | 0.04(0.03-0.05) | 3.07(2.42-3.81) | 0.06(0.04-0.08) | 2.54(1.82-3.39) | 63.65 | −0.86(−0.99-−0.74) |
| Mauritius | 0.01(0.01-0.01) | 1.29(1.17-1.42) | 0.03(0.02-0.04) | 1.83(1.46-2.28) | 180.56 | 1.27(1.11-1.43) |
| Mexico | 1.26(1.22-1.31) | 2.4(2.31-2.48) | 3.4(2.94-3.88) | 2.9(2.51-3.3) | 169.78 | 0.6(0.45-0.75) |
| Micronesia  (Federated States of) | 0(0-0) | 2.63(2.05-3.31) | 0(0-0) | 2.93(1.92-4.13) | 45.72 | 0.34(0.29-0.39) |
| Monaco | 0.01(0.01-0.01) | 10.05(8.03-12.05) | 0.01(0.01-0.02) | 15.78(12.64-19.19) | 117.18 | 1.92(1.49-2.36) |
| Mongolia | 0.04(0.03-0.04) | 3.18(2.63-3.72) | 0.06(0.05-0.08) | 2.64(2.1-3.39) | 69.61 | −1.31(−1.57-−1.04) |
| Montenegro | 0.01(0.01-0.02) | 2.29(1.93-2.61) | 0.02(0.02-0.03) | 2.45(2.04-2.9) | 60.31 | 0.53(0.43-0.62) |
| Morocco | 0.52(0.39-0.63) | 3.48(2.57-4.11) | 1.32(1-1.63) | 4.32(3.31-5.28) | 152.78 | 0.65(0.53-0.78) |
| Mozambique | 0.17(0.14-0.2) | 2.91(2.44-3.41) | 0.5(0.38-0.65) | 4.68(3.62-6.01) | 205.12 | 2.06(1.9-2.21) |
| Myanmar | 2.7(1.95-3.61) | 10.91(7.89-14.36) | 5.64(4.74-6.82) | 12.73(10.85-15.28) | 109.28 | 0.58(0.53-0.63) |
| Namibia | 0.02(0.02-0.03) | 3.01(2.25-3.78) | 0.06(0.04-0.08) | 3.61(2.59-4.77) | 137.98 | 0.5(0.28-0.71) |
| Nauru | 0(0-0) | 4.04(2.69-5.95) | 0(0-0) | 3.85(2.81-4.91) | 0.31 | −0.32(−0.44-−0.2) |
| Nepal | 0.21(0.13-0.31) | 1.65(1.15-2.21) | 0.46(0.36-0.56) | 2.05(1.6-2.53) | 114.5 | 0.87(0.62-1.12) |
| Netherlands | 1.21(1.13-1.28) | 6.14(5.75-6.49) | 1.64(1.41-1.85) | 4.74(4.13-5.35) | 35.29 | −1.39(−1.61-−1.18) |
| New Zealand | 0.22(0.21-0.24) | 5.72(5.26-6.18) | 0.36(0.31-0.41) | 4.57(3.99-5.22) | 59.6 | −1.4(−1.69-−1.11) |
| Nicaragua | 0.04(0.03-0.04) | 1.69(1.48-1.94) | 0.11(0.09-0.14) | 2.51(2.05-2.98) | 204.35 | 1.58(1.38-1.78) |
| Niger | 0.04(0.03-0.06) | 0.87(0.68-1.06) | 0.09(0.06-0.14) | 0.8(0.54-1.2) | 129.31 | −0.43(−0.54-−0.31) |
| Nigeria | 0.83(0.55-1.19) | 1.41(1.04-1.89) | 2(1.46-2.63) | 1.72(1.32-2.16) | 141.73 | 0.97(0.83-1.12) |
| Niue | 0(0-0) | 2.6(2.11-3.17) | 0(0-0) | 2.75(2.11-3.58) | −2.86 | 0.16(0.13-0.19) |
| North Macedonia | 0.04(0.04-0.04) | 2.03(1.85-2.22) | 0.06(0.05-0.08) | 2.17(1.75-2.72) | 66.44 | 0.15(0.01-0.29) |
| Northern Mariana Islands | 0(0-0) | 2.38(1.86-3.07) | 0(0-0) | 3.41(2.85-4.07) | 175.44 | 1.85(1.57-2.13) |
| Norway | 0.38(0.36-0.39) | 5.64(5.4-5.85) | 0.38(0.34-0.4) | 3.84(3.52-4.12) | 0.17 | −1.63(−1.8-−1.46) |
| Oman | 0.04(0.03-0.05) | 5.84(3.72-7.65) | 0.1(0.08-0.13) | 6.35(5.15-7.68) | 153.77 | 0.67(0.45-0.89) |
| Pakistan | 0.98(0.67-1.29) | 1.42(1.04-1.84) | 2.76(1.85-3.99) | 1.99(1.4-2.85) | 180.21 | 1.09(0.88-1.3) |
| Palau | 0(0-0) | 1.82(1.36-2.35) | 0(0-0) | 1.83(1.44-2.27) | 86.53 | −0.01(−0.03-0.02) |
| Palestine | 0.03(0.03-0.04) | 3.41(2.56-4.44) | 0.08(0.07-0.1) | 3.3(2.78-3.91) | 145.79 | −0.32(−0.51-−0.13) |
| Panama | 0.05(0.04-0.05) | 3.02(2.73-3.33) | 0.12(0.1-0.16) | 3(2.29-3.93) | 153.99 | 0(−0.08-0.08) |
| Papua New Guinea | 0.02(0.01-0.02) | 0.88(0.69-1.08) | 0.05(0.04-0.06) | 0.95(0.74-1.2) | 162.76 | 0.21(0.17-0.25) |
| Paraguay | 0.05(0.04-0.05) | 1.93(1.71-2.16) | 0.16(0.13-0.21) | 2.87(2.2-3.64) | 233.49 | 1.48(1.31-1.65) |
| Peru | 0.52(0.45-0.6) | 3.82(3.3-4.39) | 1.44(1.08-1.91) | 4.46(3.34-5.9) | 176.15 | 0.61(0.38-0.84) |
| Philippines | 1.01(0.93-1.09) | 2.71(2.44-2.97) | 2.18(1.81-2.61) | 2.58(2.15-3.08) | 116.13 | −0.33(−0.54-−0.11) |
| Poland | 1.05(1.02-1.09) | 2.47(2.39-2.55) | 2.21(1.87-2.59) | 3.31(2.8-3.89) | 109.36 | 0.81(0.43-1.19) |
| Portugal | 0.46(0.43-0.49) | 3.54(3.33-3.75) | 0.95(0.84-1.07) | 4.04(3.59-4.53) | 105.54 | 0.21(−0.06-0.47) |
| Puerto Rico | 0.18(0.17-0.2) | 5.02(4.66-5.4) | 0.25(0.19-0.32) | 3.74(2.89-4.85) | 37.77 | −1.15(−1.27-−1.02) |
| Qatar | 0.01(0.01-0.01) | 5.87(4.65-7.21) | 0.04(0.03-0.06) | 5.97(4.46-7.9) | 491.98 | 0.3(0.08-0.53) |
| Republic of Korea | 0.73(0.69-0.77) | 2.16(2.05-2.27) | 2.33(2.09-2.59) | 2.74(2.46-3.04) | 218.88 | 0.16(−0.19-0.51) |
| Republic of Moldova | 0.08(0.08-0.09) | 1.86(1.72-2.02) | 0.12(0.1-0.14) | 2.35(2.01-2.74) | 46.27 | 0.83(0.52-1.14) |
| Romania | 0.54(0.51-0.58) | 2.09(1.96-2.21) | 0.82(0.66-1.02) | 2.57(2.08-3.15) | 51.16 | 0.89(0.75-1.04) |
| Russian Federation | 3.62(3.49-3.78) | 2.11(2.04-2.2) | 4.7(4.1-5.35) | 2.15(1.88-2.45) | 29.91 | 0.04(−0.19-0.28) |
| Rwanda | 0.11(0.08-0.15) | 3.33(2.49-4.28) | 0.21(0.16-0.28) | 3.36(2.63-4.4) | 83.43 | −0.37(−0.62-−0.11) |
| Saint Kitts and Nevis | 0(0-0) | 6.82(6-7.7) | 0(0-0) | 4.58(3.58-5.72) | 16.58 | −1.28(−1.54-−1.01) |
| Saint Lucia | 0.01(0.01-0.01) | 7.07(6.41-7.77) | 0.01(0.01-0.01) | 5.31(4.33-6.44) | 62.03 | −1.2(−1.51-−0.88) |
| Saint Vincent and the Grenadines | 0.01(0.01-0.01) | 7.48(6.52-8.52) | 0.01(0.01-0.01) | 6.95(5.87-8.19) | 50.45 | −0.26(−0.45-−0.07) |
| Samoa | 0(0-0) | 2.45(1.71-3.23) | 0(0-0.01) | 2.56(1.78-3.45) | 63.83 | 0.2(0.14-0.25) |
| San Marino | 0(0-0) | 9.9(8.3-11.65) | 0.01(0-0.01) | 9.56(6.47-14) | 96.56 | 0.17(0.06-0.27) |
| Sao Tome and Principe | 0(0-0) | 2.3(1.8-2.73) | 0(0-0) | 2.97(2.18-3.89) | 93.67 | 0.59(0.38-0.79) |
| Saudi Arabia | 0.31(0.16-0.42) | 4.95(2.53-6.65) | 0.88(0.69-1.11) | 4.78(3.84-5.87) | 179.12 | −0.51(−0.67-−0.34) |
| Senegal | 0.12(0.09-0.16) | 2.76(2.19-3.31) | 0.25(0.18-0.33) | 2.82(2.11-3.69) | 106.92 | −0.13(−0.26-0.01) |
| Serbia | 0.3(0.25-0.34) | 2.73(2.3-3.15) | 0.48(0.38-0.6) | 3.23(2.57-4.01) | 63.11 | 0.98(0.8-1.17) |
| Seychelles | 0(0-0) | 3.9(3.47-4.32) | 0.01(0.01-0.01) | 5.96(5.18-6.81) | 180.28 | 1.17(0.94-1.4) |
| Sierra Leone | 0.06(0.04-0.09) | 2.45(1.89-3.17) | 0.12(0.08-0.16) | 2.44(1.72-3.28) | 91.57 | −0.21(−0.33-−0.09) |
| Singapore | 0.08(0.07-0.09) | 3.62(3.15-4.2) | 0.21(0.17-0.25) | 2.83(2.35-3.38) | 155.28 | −1.06(−1.25-−0.87) |
| Slovakia | 0.18(0.16-0.2) | 3.01(2.68-3.42) | 0.35(0.28-0.44) | 3.94(3.1-4.95) | 98.57 | 1.4(1.23-1.57) |
| Slovenia | 0.08(0.06-0.1) | 3.18(2.42-4.03) | 0.2(0.15-0.26) | 4.48(3.44-5.7) | 164.93 | 1.38(1.17-1.6) |
| Solomon Islands | 0.01(0.01-0.02) | 7.01(5.39-8.8) | 0.03(0.02-0.04) | 7.57(6.15-9.12) | 129.86 | 0.14(0.04-0.25) |
| Somalia | 0.06(0.05-0.09) | 2.18(1.53-3) | 0.17(0.09-0.27) | 2.16(1.13-3.38) | 172.28 | 0.17(0.08-0.25) |
| South Africa | 0.84(0.76-0.91) | 3.35(2.97-3.69) | 1.76(1.57-1.97) | 3.66(3.28-4.09) | 108.79 | 0.48(0.23-0.73) |
| South Sudan | 0.11(0.06-0.19) | 3.85(2.01-6.78) | 0.16(0.09-0.25) | 3.73(2.2-5.8) | 45.23 | 0.03(−0.07-0.14) |
| Spain | 2.01(1.9-2.12) | 3.9(3.7-4.1) | 3.28(2.83-3.7) | 3.36(2.95-3.78) | 62.91 | −0.99(−1.26-−0.71) |
| Sri Lanka | 0.21(0.19-0.24) | 1.83(1.65-2.03) | 0.61(0.46-0.8) | 2.5(1.89-3.24) | 186.07 | 1.64(1.32-1.95) |
| Sudan | 0.21(0.14-0.3) | 1.79(1.29-2.44) | 0.47(0.3-0.71) | 2.11(1.33-3.1) | 125.92 | 0.72(0.54-0.89) |
| Suriname | 0.01(0.01-0.01) | 3.83(3.34-4.2) | 0.02(0.02-0.03) | 3.8(3.1-4.58) | 101.11 | −0.03(−0.3-0.24) |
| Sweden | 0.77(0.72-0.83) | 5.17(4.84-5.49) | 0.9(0.79-1.01) | 3.98(3.53-4.46) | 15.97 | −1.32(−1.49-−1.15) |
| Switzerland | 0.22(0.2-0.24) | 2.14(1.95-2.34) | 0.66(0.57-0.76) | 3.62(3.19-4.14) | 205.38 | 0.89(−0.02-1.8) |
| Syrian Arab Republic | 0.15(0.12-0.19) | 2.46(1.92-3.06) | 0.2(0.14-0.26) | 1.63(1.18-2.16) | 28.66 | −2.46(−3.05-−1.86) |
| Taiwan  (Province of China) | 0.61(0.56-0.66) | 3.75(3.44-4.08) | 1.5(1.15-1.96) | 3.95(3.02-5.16) | 145.21 | 0.02(−0.15-0.19) |
| Tajikistan | 0.04(0.04-0.04) | 1.2(1.08-1.35) | 0.12(0.1-0.15) | 2.04(1.67-2.52) | 194.85 | 2.13(1.88-2.38) |
| Thailand | 0.67(0.59-0.76) | 1.67(1.46-1.9) | 1.81(1.35-2.37) | 1.88(1.41-2.44) | 168.33 | −0.03(−0.2-0.14) |
| Timor-Leste | 0.01(0-0.01) | 1.76(1.33-2.23) | 0.02(0.02-0.03) | 2.36(1.74-2.99) | 187 | 1.26(1.01-1.52) |
| Togo | 0.05(0.04-0.06) | 2.59(2.17-3.09) | 0.12(0.08-0.16) | 2.58(1.85-3.44) | 151.64 | −0.48(−0.7-−0.26) |
| Tokelau | 0(0-0) | 2.04(1.58-2.59) | 0(0-0) | 2.31(1.7-3.07) | 5.85 | 0.47(0.43-0.5) |
| Tonga | 0(0-0) | 4.37(3.61-5.18) | 0(0-0.01) | 4.93(3.91-6.35) | 56.82 | 0.33(0.23-0.42) |
| Trinidad and Tobago | 0.04(0.04-0.05) | 4.5(4.06-4.99) | 0.06(0.04-0.08) | 3.25(2.33-4.38) | 30.7 | −1.4(−1.61-−1.2) |
| Tunisia | 0.07(0.06-0.08) | 1.21(1-1.4) | 0.16(0.12-0.22) | 1.32(0.97-1.78) | 146.95 | 0.27(0.22-0.32) |
| Turkey | 1.59(1.21-1.97) | 3.92(3.03-4.83) | 2.8(2.25-3.4) | 3.24(2.61-3.93) | 76.11 | −0.53(−0.79-−0.27) |
| Turkmenistan | 0.03(0.02-0.03) | 1.14(1-1.31) | 0.06(0.05-0.08) | 1.48(1.16-1.89) | 131.62 | 0.85(0.64-1.05) |
| Tuvalu | 0(0-0) | 2.31(1.74-2.91) | 0(0-0) | 2.44(1.67-3.32) | 49.62 | 0.11(0.08-0.13) |
| Uganda | 0.12(0.1-0.15) | 1.61(1.31-1.92) | 0.45(0.35-0.58) | 2.53(2.02-3.04) | 274.05 | 1.79(1.67-1.91) |
| Ukraine | 0.92(0.86-0.99) | 1.46(1.37-1.57) | 1.66(1.38-1.99) | 2.6(2.15-3.1) | 80.42 | 2.27(1.97-2.57) |
| United Arab Emirates | 0.04(0.02-0.07) | 6.27(2.43-10.99) | 0.31(0.16-0.48) | 4.81(2.3-7.38) | 609.26 | −1.03(−1.38-−0.69) |
| United Kingdom | 4.61(4.44-4.72) | 5.27(5.09-5.38) | 6.22(5.72-6.54) | 4.82(4.49-5.04) | 34.89 | −0.73(−0.9-−0.56) |
| United Republic of Tanzania | 0.51(0.39-0.64) | 4.49(3.67-5.43) | 1.32(1.1-1.57) | 5.3(4.49-6.22) | 160.71 | 0.66(0.54-0.78) |
| United States of America | 21.7(20.7-22.27) | 6.8(6.5-6.96) | 29.99(27.74-31.57) | 5.28(4.92-5.55) | 38.19 | −1.49(−1.7-−1.28) |
| United States Virgin Islands | 0(0-0.01) | 4.87(4.04-5.72) | 0.01(0.01-0.01) | 5.07(4.22-6.12) | 98.45 | 0.23(0.1-0.37) |
| Uruguay | 0.19(0.18-0.21) | 5.22(4.88-5.58) | 0.29(0.26-0.32) | 5.6(5.06-6.17) | 49.21 | 0.05(−0.16-0.26) |
| Uzbekistan | 0.16(0.15-0.18) | 1.14(1.04-1.24) | 0.56(0.45-0.68) | 2.3(1.88-2.76) | 247.04 | 3.15(2.77-3.53) |
| Vanuatu | 0(0-0) | 1.87(1.39-2.42) | 0(0-0.01) | 2.26(1.59-3.02) | 201.04 | 0.51(0.4-0.62) |
| Venezuela | 0.43(0.4-0.47) | 3.64(3.32-3.97) | 0.87(0.65-1.16) | 3.02(2.26-4) | 102.3 | −0.91(−1.06-−0.76) |
| Viet Nam | 0.89(0.71-1.11) | 2.03(1.61-2.52) | 1.98(1.57-2.45) | 2.17(1.73-2.64) | 124.07 | 0.23(0.06-0.4) |
| Yemen | 0.11(0.07-0.15) | 1.79(1.3-2.41) | 0.29(0.2-0.4) | 1.83(1.26-2.49) | 164.07 | 0.38(0.27-0.49) |
| Zambia | 0.16(0.11-0.21) | 5.07(4.11-6.12) | 0.37(0.3-0.46) | 5.53(4.53-6.72) | 134.24 | 0.25(0.22-0.27) |
| Zimbabwe | 0.1(0.08-0.11) | 2.04(1.72-2.34) | 0.11(0.08-0.14) | 1.41(1.09-1.78) | 9.65 | −1.71(−2-−1.43) |

EAPC: estimated annual percentage change; ASR, age-standardized rate; CI, confidence interval; UI: uncertainty interval.

**Supplementary table 5.** The number and age-standardized rate of DALYs caused by non-Hodgkin lymphoma in global, sexes, SDI areas and geographic regions in 1990 and 2019, and percentage change of absolute number and the EAPCs from 1990 to 2019

|  | **1990** | | **2019** | | **1990-2019** | |
| --- | --- | --- | --- | --- | --- | --- |
| **Characteristics** | Number  ×10^3^ (95% UI) | ASR/100,000  (95% UI) | Number  ×10^3^ (95% UI) | ASR/100,000  (95% UI) | Change in  number (%) | EAPC  (95%CI) |
| **Overall** | 4146.98  (3814.13-4455.09) | 90.43  (84.41-96.22) | 6991.33  (6570.14-7450.47) | 86.50  (81.26-92.26) | 68.59 | −0.28  (−0.35-−0.22) |
| **Sex** |  |  |  |  |  |  |
| Male | 2478.61  (2270.66-2699.4) | 111.01  (103.29-119.33) | 4218.18  (3925.16-4529.04) | 108.24  (100.94-116.24) | 70.18 | −0.12  (−0.18-−0.06) |
| Female | 1668.38  (1525.96-1825.52) | 71.11  (65.64-77.12) | 2773.15  (2564.29-2984.1) | 66.13  (61.15-71.24) | 66.22 | −0.52  (−0.63-−0.42) |
| **SDI** |  |  |  |  |  |  |
| Low | 287.20  (201.33-369.45) | 71.79  (58.89-85.12) | 582.30  (502.18-668.43) | 73.26  (62.87-84.64) | 102.75 | 0.10  (0.05-0.16) |
| Low-middle | 626.81  (509.63-749.03) | 69.40  (59.61-80.81) | 1283.12  (1151.5-1421.59) | 81.36  (73.13-90.06) | 104.70 | 0.58  (0.54-0.61) |
| Middle | 982.20  (887.32-1066.42) | 67.61  (61.76-72.78) | 1950.85  (1789-2135.22) | 76.78  (70.42-84.06) | 98.62 | 0.59  (0.49-0.69) |
| High-middle | 931.07  (886.82-974.78) | 82.82  (78.89-86.65) | 1475.49  (1372.45-1583.05) | 80.98  (75.44-86.81) | 58.47 | −0.19  (−0.26-−0.11) |
| High | 1316.81  (1279.58-1345.69) | 134.67  (131.02-137.45) | 1695.19  (1589.45-1779.53) | 101.18  (96.09-105.78) | 28.73 | −1.48  (−1.65-−1.31) |
| **Regions** |  |  |  |  |  |  |
| East Asia | 686.92  (607.67-772.5) | 62.65  (55.71-70.27) | 1362.18  (1165.31-1574.93) | 71.57  (61.57-82.02) | 98.30 | 0.68  (0.47-0.89) |
| South Asia | 440.45  (351.37-533.22) | 50.79  (42.85-59.68) | 1099.82  (946.83-1271.75) | 67.47  (58.14-78.11) | 149.70 | 1.01  (0.94-1.09) |
| Southeast Asia | 334.77  (268.19-403.04) | 90.99  (76.99-106.62) | 628.00  (539.36-725.99) | 95.41  (82.26-110.1) | 87.59 | 0.09  (0.06-0.13) |
| Central Asia | 28.96  (27.54-30.4) | 47.50  (45.33-49.71) | 57.48  (50.65-65.27) | 64.78  (57.5-73.36) | 98.47 | 0.81  (0.62-1.01) |
| High-income  Asia Pacific | 181.13  (176.37-185.27) | 92.87  (90.35-95.02) | 293.94  (264.82-315.78) | 76.60  (70.64-81.43) | 62.28 | −0.95  (−1.09-−0.81) |
| Oceania | 2.40  (2.00-2.90) | 50.57  (43.19-58.86) | 5.08  (4.23-6.09) | 50.06  (42.12-59.92) | 112.02 | −0.05  (−0.1-−0.01) |
| Australasia | 37.31  (35.52-39.08) | 163.16  (155.39-170.67) | 46.99  (42.04-52.11) | 103.62  (93.65-114.64) | 25.94 | −2.26  (−2.51-−2.01) |
| Eastern Europe | 173.97  (167.95-181) | 71.07  (68.56-73.96) | 210.92  (189.23-233.13) | 76.29  (68.59-84.06) | 21.24 | 0.20  (−0.02-0.42) |
| Western Europe | 630.49  (614.35-644.72) | 124.46  (121.57-127.15) | 753.51  (696.52-801.3) | 96.95  (91.08-102.72) | 19.51 | −1.34  (−1.52-−1.15) |
| Central Europe | 118.66  (114.57-123.23) | 87.12  (83.96-90.66) | 155.32  (136.21-175.89) | 89.38  (78.58-101.26) | 30.90 | 0.07  (−0.07-0.20) |
| High-income North America | 582.84  (565.38-597.35) | 176.29  (171.54-180.45) | 681.86  (645.1-715.33) | 118.34  (112.89-123.59) | 16.99 | −2.04  (−2.27-−1.82) |
| Andean Latin America | 31.97  (27.99-35.93) | 108.69  (96.31-121.50) | 76.28  (61.49-94.72) | 128.35  (103.32-158.9) | 138.60 | 0.65  (0.50-0.80) |
| Central Latin America | 110.93  (107.05-115.25) | 85.45  (82.76-88.21) | 207.64  (179.47-243.03) | 84.39  (73.09-98.66) | 87.19 | 0  (−0.05-0.05) |
| Caribbean | 45.38  (39.96-50.91) | 143.68  (129.19-158.31) | 58.92  (49.22-69.52) | 118.97  (99.14-141) | 29.84 | −0.61  (−0.83-−0.39) |
| Tropical Latin America | 121.46  (116.06-126.93) | 95.40  (91.75-99.01) | 189.78  (180.56-199.38) | 79.37  (75.41-83.59) | 56.25 | −0.58  (−0.69-−0.47) |
| Southern Latin America | 61.51  (59.28-63.54) | 128.50  (123.86-132.66) | 86.47  (79.95-93.75) | 111.81  (103.48-121.26) | 40.58 | −0.75  (−0.93-−0.58) |
| Eastern Sub-  Saharan Africa | 101.86  (69.15-134.51) | 76.62  (61.39-92.05) | 203.44  (166.9-247.95) | 80.81  (68.27-95.99) | 99.73 | 0.32  (0.21-0.44) |
| Southern Sub-  Saharan Africa | 41.09  (37.63-44.65) | 102.42  (93.03-110.59) | 73.89  (65.38-83.63) | 104.35  (93.21-117.29) | 79.84 | 0.21  (−0.05-0.47) |
| Western Sub-  Saharan Africa | 120.63  (84.94-161.36) | 72.62  (58-90.32) | 250.04  (198.19-308.4) | 73.08  (59.09-87.26) | 107.28 | −0.15  (−0.28-−0.02) |
| North Africa and Middle East | 258.69  (207.4-311.67) | 102.14  (85.16-119.54) | 483.36  (420.8-553.93) | 92.10  (80.26-105.42) | 86.85 | −0.38  (−0.5-−0.25) |
| Central Sub-  Saharan Africa | 35.57  (22.26-50.98) | 81.98  (66.59-100.15) | 66.4  (49.30-84.44) | 75.40  (53.55-102.72) | 86.66 | −0.21  (−0.36-−0.05) |

EAPC: estimated annual percentage change; ASR, age-standardized rate; CI, confidence interval; UI: uncertainty interval; SDI: socio-demographic index.

**Supplementary table 6**. the number and age-standardized rate of DALYs caused by non-Hodgkin lymphoma at national level and both sexes in 1990 and 2019, and percentage changes in number and the EAPCs from 1990 to 2019

|  | **1990** | | **2019** | | **1990-2019** | |
| --- | --- | --- | --- | --- | --- | --- |
| **Characteristics** | Number  ×10^3^ (95% UI) | ASR/100,000  (95% UI) | Number  ×10^3^ (95% UI) | ASR/100,000  (95% UI) | Percentage changes (%) | EAPC  (95%CI) |
| Afghanistan | 32.31(22.32-41.46) | 397.39(261.05-515.8) | 70.05(46.18-102.86) | 393.88(235.93-632.37) | 116.8 | 0.1(−0.03-0.24) |
| Albania | 1.63(1.49-1.76) | 57.26(52.92-61.89) | 1.81(1.35-2.36) | 53.05(40.23-68.31) | 11.43 | −0.03(−0.29-0.23) |
| Algeria | 29.1(18.64-41.55) | 146.02(99.14-203.14) | 41.62(33.82-50.66) | 111.76(91.22-135.31) | 43.02 | −0.86(−0.96-−0.77) |
| American Samoa | 0.02(0.02-0.03) | 70.66(55.52-84.98) | 0.04(0.04-0.06) | 87.33(69.67-110.87) | 84.78 | 1.12(0.81-1.44) |
| Andorra | 0.1(0.07-0.14) | 177.65(132.53-240.07) | 0.18(0.14-0.24) | 146.17(111.14-187.38) | 82.86 | −0.74(−0.77-−0.7) |
| Angola | 8.03(4.16-13.67) | 96.68(67.47-139.93) | 19.7(14-26.62) | 96.89(70.66-126.35) | 145.37 | 0.2(0.08-0.31) |
| Antigua and Barbuda | 0.07(0.06-0.08) | 125.1(112.8-138.42) | 0.11(0.09-0.13) | 107.5(87.92-128.95) | 53.18 | −0.58(−0.76-−0.4) |
| Argentina | 42.9(41.14-44.65) | 131.34(125.93-136.77) | 58.63(53.08-64.93) | 116.04(105.34-128.69) | 36.65 | −0.79(−0.97-−0.61) |
| Armenia | 1.2(1.1-1.29) | 38.01(35.18-41.01) | 2.11(1.74-2.52) | 59.5(49.4-70.94) | 76.76 | 2.25(1.77-2.74) |
| Australia | 31.77(30.07-33.48) | 166.59(157.72-175.33) | 39.37(34.64-44.37) | 102.53(90.75-114.97) | 23.94 | −2.37(−2.62-−2.12) |
| Austria | 9.56(9.07-10.12) | 89.62(85.14-94.52) | 13.56(12.18-15.01) | 85.59(77.4-94.2) | 41.83 | −0.16(−0.39-0.07) |
| Azerbaijan | 2.54(2.28-2.87) | 39.01(35.11-43.58) | 5.94(4.83-7.38) | 57.1(46.38-70.82) | 133.75 | 1.74(1.36-2.12) |
| Bahamas | 0.34(0.3-0.38) | 159.03(141.45-178.83) | 0.55(0.44-0.7) | 134.14(106.77-170.17) | 61.46 | −0.48(−0.61-−0.34) |
| Bahrain | 0.31(0.26-0.36) | 105.54(89.79-124.96) | 1.16(0.89-1.47) | 98.88(77.96-121.43) | 276.58 | 0.23(−0.11-0.57) |
| Bangladesh | 42.52(29.62-56.94) | 50.51(38.65-63.63) | 73.09(53.46-100.59) | 49.17(36.16-67.13) | 71.87 | 0.02(−0.09-0.12) |
| Barbados | 0.56(0.5-0.61) | 211.04(190.79-232.16) | 0.72(0.57-0.88) | 179.98(145.11-223.64) | 28.56 | −0.7(−0.79-−0.62) |
| Belarus | 5.32(4.91-5.75) | 47.44(43.59-51.48) | 9.5(7.24-12.38) | 74.72(57.26-98.13) | 78.49 | 2.29(1.79-2.8) |
| Belgium | 15.65(14.8-16.52) | 114.08(108.13-120.74) | 18.37(16.02-20.92) | 91.86(80.89-104.32) | 17.39 | −1.22(−1.43-−1.01) |
| Belize | 0.09(0.08-0.1) | 58.65(52.29-65.34) | 0.25(0.21-0.29) | 67.35(56.95-78.99) | 174.53 | 0.15(−0.08-0.38) |
| Benin | 3.49(2.37-4.8) | 88.23(71.33-108.66) | 8.43(5.66-11.99) | 89.66(64.75-121.61) | 141.84 | −0.21(−0.41-−0.02) |
| Bermuda | 0.14(0.13-0.15) | 222.05(201.09-243.52) | 0.11(0.09-0.14) | 117.45(96.99-143.1) | −19 | −2.15(−2.43-−1.88) |
| Bhutan | 0.21(0.11-0.33) | 47.54(29.68-69.9) | 0.44(0.3-0.61) | 67.07(46.83-90.79) | 109.48 | 1.18(1.11-1.25) |
| Bolivia | 5.52(4.15-6.89) | 115.01(90.39-139.57) | 13.68(10.66-17.21) | 137.64(107.94-172.14) | 147.96 | 0.53(0.46-0.6) |
| Bosnia and Herzegovina | 2.37(2.2-2.56) | 52.22(48.42-56.47) | 3.6(2.83-4.54) | 77.17(60.95-96.7) | 51.69 | 1.7(1.42-1.97) |
| Botswana | 0.48(0.36-0.65) | 57.31(42.26-76.52) | 1.55(1.05-2.18) | 77.35(54.32-106.22) | 223.18 | 0.44(0.02-0.86) |
| Brazil | 119.6(114.22-124.98) | 96.37(92.65-99.96) | 184.74(175.93-194.06) | 79.37(75.57-83.44) | 54.46 | −0.61(−0.72-−0.5) |
| Brunei Darussalam | 0.39(0.29-0.49) | 231.65(177.99-287.68) | 0.81(0.71-0.93) | 218.74(191.69-250.5) | 106.54 | 0.33(0.04-0.62) |
| Bulgaria | 7.74(7.16-8.38) | 76.31(70.24-83.03) | 9.77(7.63-12.51) | 98.01(76.22-125.72) | 26.14 | 1.22(1.01-1.43) |
| Burkina Faso | 6.15(3.83-8.98) | 77.61(58.88-101.34) | 15.7(10.26-22.12) | 89.83(66.31-114.48) | 155.45 | 0.12(−0.11-0.35) |
| Burundi | 3.44(2.1-5.04) | 86.89(61.01-118.93) | 5.69(3.83-8.19) | 74.32(50.57-104.61) | 65.26 | −0.66(−0.75-−0.56) |
| Cabo Verde | 0.12(0.1-0.14) | 42.31(37.53-47.62) | 0.53(0.44-0.65) | 105.23(88.48-126) | 340.8 | 2.55(1.92-3.17) |
| Cambodia | 5.97(3.7-8.62) | 74.39(53.87-99.37) | 11.85(9.38-14.55) | 82.54(65.98-99.99) | 98.71 | 0.4(0.29-0.5) |
| Cameroon | 8.58(6.58-11.35) | 105.78(78.35-134.31) | 25.53(15.5-40.6) | 112.38(69.19-180.61) | 197.41 | 0.17(0.1-0.24) |
| Canada | 51.19(47.98-54.51) | 163.31(153.22-174.07) | 74.11(61.85-88) | 120.36(101.68-140.77) | 44.76 | −1.63(−1.85-−1.41) |
| Central African Republic | 1.79(1.19-2.4) | 88.68(72.19-108.95) | 3.07(2.08-4.45) | 81.02(55.93-113.94) | 71.34 | −0.17(−0.27-−0.08) |
| Chad | 3.65(2.56-4.97) | 72.39(57.17-88.56) | 10.32(7.21-14.12) | 87.38(65.85-112.42) | 183.06 | 0.44(0.22-0.66) |
| Chile | 13.01(12.27-13.87) | 112.46(106.18-119.48) | 20.85(19.07-22.97) | 93.6(85.49-103.4) | 60.29 | −0.66(−0.86-−0.46) |
| China | 650.08(572.42-734.67) | 61.41(54.34-69.17) | 1306.25(1103.33-1521.35) | 71(60.64-81.81) | 100.94 | 0.74(0.52-0.96) |
| Colombia | 26(24.61-27.59) | 100.61(95.43-106.54) | 45.25(35.11-58.2) | 89.42(69.49-114.94) | 73.99 | −0.17(−0.35-0.01) |
| Comoros | 0.24(0.09-0.36) | 74.61(35.5-107.38) | 0.47(0.3-0.66) | 81.04(55.03-111.29) | 97.14 | 0.13(−0.1-0.37) |
| Congo | 1.96(1.25-2.9) | 114.96(83.51-156.72) | 3.79(2.8-5.03) | 99.49(74.66-129.42) | 92.98 | −0.52(−0.66-−0.37) |
| Cook Islands | 0.01(0.01-0.01) | 57.75(47.93-70.58) | 0.01(0.01-0.02) | 57.31(43.9-72.58) | 38.45 | 0.18(−0.04-0.39) |
| Costa Rica | 2.82(2.59-3.08) | 123.28(112.88-134.73) | 6.42(4.91-8.29) | 126.14(96.62-162.7) | 127.37 | 0.25(−0.08-0.59) |
| Croatia | 5.87(5.35-6.42) | 99.37(91.11-108.94) | 7.16(5.51-9.05) | 99.96(77.85-126.87) | 22.03 | 0.1(−0.2-0.41) |
| Cuba | 17.48(16.47-18.57) | 162.96(153.63-172.84) | 17.17(13.79-21.22) | 111.71(90.01-138.41) | −1.78 | −1.26(−1.68-−0.83) |
| Cyprus | 1.16(0.98-1.33) | 142.09(119.47-163.84) | 1.83(1.58-2.09) | 99.24(85.86-113.57) | 57.83 | −1.7(−1.93-−1.47) |
| Czechia | 14.17(13.2-15.09) | 113.57(105.87-120.93) | 15.01(11.96-18.65) | 82.75(65.82-102.09) | 5.91 | −1.13(−1.22-−1.04) |
| Côte d'Ivoire | 9.18(5.75-13.57) | 105.57(73.88-146.18) | 19.14(12.88-26.23) | 98.85(69.7-133.27) | 108.58 | −0.71(−0.97-−0.44) |
| Democratic People's Republic of Korea | 15.08(10.97-20.21) | 75.27(56.31-99.04) | 20.44(14.05-27.33) | 65.9(45.37-88.31) | 35.53 | −0.27(−0.38-−0.16) |
| Democratic Republic of the Congo | 22.5(14.24-33.92) | 73.73(59.46-91.87) | 37.18(23.85-52.64) | 64.29(38.16-96.69) | 65.27 | −0.46(−0.69-−0.22) |
| Denmark | 6.5(5.91-7.15) | 92.51(83.84-101.83) | 9.05(7.49-10.81) | 90.03(75.65-106.25) | 39.2 | −0.9(−1.35-−0.44) |
| Djibouti | 0.23(0.15-0.36) | 83.25(53.43-123.43) | 0.85(0.56-1.27) | 101.28(70.16-144.87) | 269.32 | 0.7(0.62-0.78) |
| Dominica | 0.17(0.15-0.19) | 243.53(212.55-276.05) | 0.18(0.15-0.23) | 236.34(186.72-297.56) | 8.72 | −0.03(−0.1-0.03) |
| Dominican Republic | 3.76(3.26-4.3) | 61.2(53.47-69.5) | 8.79(6.05-11.93) | 84.88(58.94-114.18) | 133.96 | 1.48(1.22-1.75) |
| Ecuador | 6.41(5.95-6.89) | 82.23(77.08-87.49) | 21.54(17.09-27.74) | 132.05(104.95-169.88) | 235.94 | 1.99(1.69-2.28) |
| Egypt | 27.85(24.57-31.93) | 58.1(52.33-64.45) | 52.35(39.86-68.48) | 60.61(46.31-79.64) | 87.93 | 0.3(0.2-0.39) |
| El Salvador | 2.2(2.03-2.38) | 51.75(48.59-55.08) | 4.52(3.42-5.92) | 74.84(56.42-98.26) | 105.54 | 0.97(0.66-1.28) |
| Equatorial Guinea | 0.26(0.16-0.37) | 77.88(58.19-102.96) | 0.95(0.56-1.59) | 112.52(69.72-178.15) | 266.11 | 1.94(1.69-2.19) |
| Eritrea | 1.32(0.92-1.76) | 69.83(52.95-90.31) | 4.02(2.77-5.76) | 94.06(67.82-127.73) | 205.45 | 0.86(0.6-1.12) |
| Estonia | 1.12(1.02-1.22) | 63.88(58.08-69.98) | 2.01(1.57-2.51) | 97.56(76.26-122.96) | 79.36 | 1.17(0.69-1.65) |
| Eswatini | 0.48(0.37-0.6) | 97.59(76.4-120.73) | 1.18(0.75-1.72) | 136.17(88.42-195.93) | 148.06 | 1.31(0.81-1.81) |
| Ethiopia | 12.06(7.82-17.61) | 37.68(28.19-52.32) | 17.28(12.67-23.62) | 28.32(20.43-40.04) | 43.26 | −1.17(−1.39-−0.95) |
| Fiji | 0.32(0.26-0.39) | 56.58(45.93-68.52) | 0.65(0.51-0.84) | 76.54(59.85-96.9) | 103.65 | 1.41(1.2-1.62) |
| Finland | 9.23(8.59-9.89) | 141.53(131.85-151.96) | 11.18(9.23-13.5) | 106.53(88.25-128.45) | 21.13 | −1.18(−1.31-−1.05) |
| France | 96.63(91.68-101.92) | 132.03(125.43-139.12) | 111.71(92.36-133.63) | 95.29(80.35-112.07) | 15.6 | −1.61(−1.78-−1.44) |
| Gabon | 1.03(0.62-1.72) | 134.12(83.52-215.08) | 1.71(1.21-2.39) | 125.49(90.47-170.22) | 65.71 | −0.24(−0.34-−0.15) |
| Gambia | 0.16(0.1-0.24) | 22.99(16.17-31.26) | 0.41(0.29-0.56) | 26.41(18.84-35.67) | 154.66 | 0.26(0.05-0.48) |
| Georgia | 2.05(1.8-2.31) | 33.89(30-38.32) | 4.48(3.64-5.39) | 94.59(76.99-113.9) | 118.88 | 4.97(4.42-5.52) |
| Germany | 124.29(119.19-129.48) | 112.49(108.06-117) | 151.62(136.31-168.55) | 91.92(83.19-101.71) | 21.99 | −1.11(−1.3-−0.92) |
| Ghana | 28.85(21.92-37.62) | 215.13(170-269.05) | 38.66(27.5-50.38) | 158(118.28-201.72) | 33.99 | −1.83(−2.28-−1.37) |
| Greece | 9.24(8.67-9.86) | 69.58(65.32-74.51) | 11.55(10.33-12.78) | 65.1(59.09-72.34) | 25.04 | −0.45(−0.77-−0.14) |
| Greenland | 0.07(0.06-0.09) | 168.69(146.69-193.62) | 0.08(0.06-0.1) | 113.41(90.75-138.55) | 6.45 | −1.75(−1.95-−1.55) |
| Grenada | 0.29(0.26-0.33) | 395.41(346.66-448.87) | 0.29(0.25-0.34) | 259.99(224.39-301.27) | −0.13 | −1.23(−1.41-−1.05) |
| Guam | 0.13(0.11-0.15) | 119.66(103.19-138.45) | 0.2(0.16-0.24) | 111.48(92.29-134.66) | 53.02 | −0.28(−0.92-0.37) |
| Guatemala | 2.76(2.42-3.13) | 45.54(39.89-51.47) | 8.68(6.69-11.09) | 60.18(46.64-77.14) | 214.2 | 1.54(1.21-1.88) |
| Guinea | 1.43(1.11-1.85) | 25.82(19.53-32.06) | 2.81(1.9-4.12) | 28.09(18.85-41.99) | 96.25 | 0.36(0.32-0.4) |
| Guinea-Bissau | 0.94(0.48-1.44) | 120.38(70.73-168.61) | 1.35(1-1.83) | 104.7(77.93-140.23) | 44.23 | −0.74(−0.92-−0.56) |
| Guyana | 0.61(0.5-0.71) | 101.74(85.28-118.39) | 0.74(0.55-0.98) | 98.99(73.49-132.48) | 20.98 | 0.12(0-0.23) |
| Haiti | 9.78(5.24-14.89) | 167.35(107.76-230.61) | 13.37(9.38-18.63) | 125.56(88.4-170.76) | 36.68 | −0.72(−0.97-−0.48) |
| Honduras | 1.85(1.56-2.19) | 54.49(45.96-63.57) | 4.53(3.26-6.39) | 63.12(47.28-87.97) | 145.11 | 0.62(0.57-0.68) |
| Hungary | 13.6(12.86-14.36) | 105.38(99.49-111.68) | 13.99(11.36-17.16) | 87.73(71.38-108.16) | 2.83 | −1.06(−1.23-−0.89) |
| Iceland | 0.26(0.23-0.3) | 95.4(84.27-109.5) | 0.38(0.31-0.47) | 78.69(64.25-95.6) | 46.58 | −0.82(−0.98-−0.67) |
| India | 345.18(284.7-414.56) | 51.19(43.99-59.87) | 888.7(751.83-1054.72) | 69.11(58.49-82.14) | 157.46 | 1.07(0.98-1.15) |
| Indonesia | 104.7(75.27-131.5) | 66.94(51.7-81.33) | 193.18(150.36-245.63) | 78.75(61.66-98.97) | 84.51 | 0.51(0.46-0.57) |
| Iran  (Islamic Republic of) | 26.09(21.18-31.26) | 60.53(51.3-69.64) | 53.06(49.25-56.84) | 65.4(60.55-70.09) | 103.42 | 0.32(0.2-0.45) |
| Iraq | 14.55(9.98-20.17) | 110.94(79.67-150.02) | 24.24(18.73-30.98) | 74.34(57.88-93.73) | 66.54 | −1.4(−1.65-−1.15) |
| Ireland | 5.23(4.91-5.58) | 133.72(125.52-142.47) | 7.57(6.69-8.61) | 109.51(96.73-124.18) | 44.87 | −1.2(−1.5-−0.91) |
| Israel | 7.21(6.65-7.84) | 149.91(138.17-163.44) | 15.02(13.33-16.99) | 137.98(122.87-155.85) | 108.38 | −0.91(−1.24-−0.58) |
| Italy | 104.27(101.25-106.94) | 136.91(133.14-140.47) | 117.72(108.35-125.52) | 102.37(95.95-108.92) | 12.9 | −1.55(−1.79-−1.3) |
| Jamaica | 2.54(2.28-2.81) | 127.67(114.4-141.41) | 5.25(4.02-6.81) | 176.71(135.18-228.76) | 107.08 | 0.46(−0.18-1.09) |
| Japan | 149.04(144.37-152.69) | 94.68(91.85-96.99) | 232.64(206.3-252.12) | 77.41(71.38-82.61) | 56.09 | −0.87(−0.99-−0.76) |
| Jordan | 3.65(2.81-4.69) | 172.63(136.53-219.02) | 10.65(9.02-12.69) | 122.8(104.31-146.46) | 192.01 | −1.59(−1.82-−1.36) |
| Kazakhstan | 10.29(9.41-11.18) | 66.01(60.9-71.43) | 9.2(7.81-10.83) | 49.72(42.34-58.35) | −10.63 | −2.92(−3.59-−2.25) |
| Kenya | 9.2(7.5-11.06) | 62.79(47.96-73.61) | 32.94(25.36-43.15) | 101.18(77.99-133.5) | 258.01 | 2.27(1.98-2.57) |
| Kiribati | 0.05(0.04-0.07) | 95.25(76.82-116.27) | 0.09(0.07-0.12) | 90.66(68.78-119.76) | 65.94 | −0.2(−0.25-−0.15) |
| Kuwait | 1.24(1.09-1.43) | 115.07(101.9-132.12) | 2.7(2.2-3.32) | 84.34(68.55-103.8) | 116.84 | −0.53(−0.91-−0.16) |
| Kyrgyzstan | 1.47(1.34-1.62) | 38.86(35.42-42.83) | 2.23(1.91-2.6) | 39.95(34.14-46.42) | 51.28 | 0.28(0.11-0.45) |
| Lao People's Democratic Republic | 3.08(1.56-4.79) | 89.75(52.44-133.73) | 4.82(3.3-6.66) | 82.03(58.03-110.92) | 56.82 | −0.39(−0.46-−0.33) |
| Latvia | 1.78(1.62-1.96) | 60.43(55.07-66.25) | 2.69(2.2-3.31) | 93.85(76.59-114.63) | 51.37 | 1.61(1.03-2.19) |
| Lebanon | 2.4(1.84-3.01) | 88.83(69.09-110.37) | 4.4(3.48-5.91) | 85.44(67.74-114.5) | 83.21 | 0.09(−0.08-0.26) |
| Lesotho | 0.83(0.57-1.15) | 64.8(44.52-88.81) | 2.02(1.4-2.81) | 119.07(83.21-161.69) | 143.14 | 2.72(2.47-2.97) |
| Liberia | 1.73(1.03-2.63) | 95.03(67.34-129.99) | 2.59(1.43-3.84) | 74.22(41.08-110.04) | 49.87 | −0.7(−0.99-−0.42) |
| Libya | 2.38(1.67-3.28) | 83.26(59.78-112.74) | 4.92(3.53-7.09) | 77.91(55.98-111.65) | 106.8 | 0.06(−0.12-0.24) |
| Lithuania | 2.12(1.93-2.31) | 53.02(48.27-57.77) | 3.6(2.92-4.45) | 82.42(67.03-101.31) | 69.72 | 1.71(1.21-2.21) |
| Luxembourg | 0.57(0.53-0.63) | 116.42(107.02-126.47) | 0.81(0.68-0.96) | 90.65(76.59-107.52) | 40.43 | −1.21(−1.43-−0.99) |
| Madagascar | 6.99(4.68-9.87) | 76.2(59.7-97.78) | 11.66(8.99-15.03) | 67.71(51.55-88.72) | 66.81 | −0.29(−0.4-−0.18) |
| Malawi | 20.53(10.22-33.37) | 211.31(131.16-305.2) | 12.47(9.74-15.68) | 112.38(91.17-137.03) | −39.28 | −2.71(−2.95-−2.47) |
| Malaysia | 14.02(12.58-15.55) | 111.8(102.24-122.8) | 38.42(30.15-47.88) | 129.7(101.98-161.09) | 173.93 | 0.38(0.28-0.49) |
| Maldives | 0.13(0.07-0.18) | 84.68(52.96-114.22) | 0.25(0.21-0.29) | 61.16(51.06-72.76) | 88.56 | −1.44(−1.69-−1.19) |
| Mali | 1.7(1.19-2.34) | 22.9(19.07-27.83) | 3.98(2.88-5.4) | 23.53(16.95-32.5) | 134.47 | 0.01(−0.1-0.13) |
| Malta | 0.43(0.38-0.48) | 102.18(90.68-115.86) | 0.72(0.6-0.86) | 97.88(81.13-115.46) | 70.05 | −0.28(−0.43-−0.13) |
| Marshall Islands | 0.02(0.02-0.03) | 77.83(64.91-90.72) | 0.04(0.03-0.05) | 87.99(64.7-112.61) | 93.08 | 0.48(0.41-0.56) |
| Mauritania | 1.5(1.09-1.95) | 98.52(74.82-123.65) | 2.27(1.41-3.39) | 77.04(50.35-110.25) | 51.68 | −1.04(−1.17-−0.91) |
| Mauritius | 0.41(0.37-0.44) | 42.7(38.79-46.53) | 0.95(0.75-1.19) | 63.35(50.17-78.49) | 133.7 | 1.46(1.28-1.63) |
| Mexico | 53.9(51.03-57.2) | 78.13(75.16-81.34) | 103.48(89.68-117.99) | 84.09(73.09-95.83) | 91.99 | 0.25(0.13-0.38) |
| Micronesia  (Federated States of) | 0.06(0.05-0.08) | 83.71(64.34-108.82) | 0.08(0.05-0.12) | 90.55(53.47-132.9) | 34.52 | 0.25(0.21-0.28) |
| Monaco | 0.15(0.12-0.18) | 276.45(222.22-334.25) | 0.3(0.24-0.37) | 409.87(323.75-507.39) | 106.5 | 1.65(1.26-2.04) |
| Mongolia | 1.42(1.18-1.67) | 93.77(77.42-110.92) | 2.25(1.73-2.94) | 75.83(59.32-98.39) | 57.81 | −1.4(−1.65-−1.15) |
| Montenegro | 0.49(0.41-0.56) | 76.74(65.05-88.37) | 0.61(0.51-0.73) | 72.87(61.14-86.32) | 25.78 | 0(−0.12-0.13) |
| Morocco | 18.85(14.45-23.35) | 100.61(76.43-121.06) | 38.86(29.42-49.99) | 114.99(87.99-144.35) | 106.11 | 0.3(0.21-0.39) |
| Mozambique | 5.28(4.19-6.45) | 68.77(56.86-81.04) | 16.63(12.17-21.86) | 112.18(84.75-145.46) | 214.8 | 2.22(2.01-2.43) |
| Myanmar | 97.66(68.86-137.75) | 316.34(229.55-429.55) | 164.6(131.84-204.66) | 329.94(268.77-404.45) | 68.54 | 0.16(0.1-0.21) |
| Namibia | 0.84(0.56-1.07) | 89.07(62.48-112.74) | 1.99(1.26-2.88) | 107.58(71.28-152.23) | 138.03 | 0.55(0.31-0.79) |
| Nauru | 0.01(0.01-0.02) | 131.04(81.37-200.13) | 0.01(0.01-0.01) | 122.93(84.23-160.66) | 0.31 | −0.36(−0.46-−0.25) |
| Nepal | 11.01(5.53-17.82) | 59.94(36.26-87.27) | 15.57(11.8-19.47) | 58.68(45.5-72.88) | 41.35 | 0.1(−0.18-0.38) |
| Netherlands | 28.92(27.21-30.76) | 156.26(146.98-165.67) | 33.36(28.76-37.84) | 109.92(95.56-124.15) | 15.34 | −1.82(−2.06-−1.57) |
| New Zealand | 5.54(5.17-5.96) | 145.79(135.87-156.87) | 7.62(6.64-8.74) | 109.77(96.35-125.08) | 37.38 | −1.66(−1.96-−1.35) |
| Nicaragua | 1.85(1.52-2.29) | 57.85(50.41-68.12) | 3.78(3.06-4.67) | 70.41(57.1-86.19) | 104.59 | 0.99(0.8-1.17) |
| Niger | 2.28(1.38-3.5) | 31.6(24.64-40.13) | 4.8(3.2-6.65) | 26.71(17.85-40.33) | 110.2 | −0.74(−0.91-−0.58) |
| Nigeria | 39.93(22.35-62.02) | 49.68(32.99-71.32) | 93.05(64.52-128.38) | 55.8(40.81-73.63) | 133.05 | 0.65(0.52-0.79) |
| Niue | 0(0-0) | 82.17(64.21-103.47) | 0(0-0) | 85.15(61.95-115.19) | −9.03 | 0.03(−0.01-0.08) |
| North Macedonia | 1.43(1.3-1.6) | 71.27(64.93-79.54) | 1.91(1.52-2.43) | 68.41(54.26-86.11) | 33.42 | −0.28(−0.41-−0.15) |
| Northern Mariana Islands | 0.02(0.02-0.03) | 70.9(54.27-93.95) | 0.05(0.04-0.06) | 100.04(80.76-124.48) | 101.94 | 1.77(1.5-2.04) |
| Norway | 8.63(8.31-8.94) | 148.36(143.35-153.13) | 7.63(7.08-8.24) | 88.27(82.57-95.19) | −11.6 | −2.16(−2.36-−1.95) |
| Oman | 1.37(0.95-1.86) | 145.68(96.05-195.47) | 3.46(2.58-4.52) | 144.52(114.02-174.94) | 151.47 | 0.31(0.01-0.61) |
| Pakistan | 41.52(25.57-58.53) | 47.02(31.63-62.24) | 122.03(77.46-178.06) | 67.09(44.73-96.9) | 193.91 | 1.16(0.95-1.36) |
| Palau | 0.01(0.01-0.01) | 56.75(41.39-74.41) | 0.01(0.01-0.02) | 57.85(44.65-72.56) | 69.55 | 0.06(0.02-0.09) |
| Palestine | 1.27(0.89-1.71) | 98.17(71.54-131.71) | 2.96(2.52-3.47) | 89.45(75.87-105.24) | 133.27 | −0.52(−0.72-−0.32) |
| Panama | 1.68(1.5-1.87) | 88.38(80-97.4) | 3.6(2.68-4.72) | 86.61(64.77-113.79) | 114.72 | 0.01(−0.08-0.1) |
| Papua New Guinea | 0.75(0.54-0.99) | 25.53(19.76-32.11) | 1.88(1.39-2.51) | 26.78(20.48-34.64) | 151.41 | 0.11(0.08-0.15) |
| Paraguay | 1.86(1.65-2.07) | 58.7(52.37-65.74) | 5.04(3.83-6.44) | 81.19(61.84-103.46) | 171.51 | 1.15(0.98-1.32) |
| Peru | 20.04(17.09-23.25) | 118.79(101.99-137.81) | 41.05(30.31-54.74) | 124.03(92.08-165.31) | 104.87 | 0.15(−0.04-0.35) |
| Philippines | 43.91(38.86-48.65) | 89.24(82.26-96.27) | 81.4(68.1-96.94) | 82.55(69.25-98.18) | 85.39 | −0.38(−0.58-−0.18) |
| Poland | 33.62(32.44-34.74) | 81.19(78.36-83.89) | 53.39(45.14-62.77) | 91.09(77.5-106.67) | 58.78 | 0.17(−0.15-0.48) |
| Portugal | 13.19(12.43-13.99) | 110.7(103.84-118.04) | 19.99(17.59-22.55) | 106.36(94.85-120.08) | 51.53 | −0.51(−0.77-−0.24) |
| Puerto Rico | 5.16(4.8-5.55) | 142.18(132.34-152.86) | 5.76(4.4-7.49) | 105.28(80.25-137.83) | 11.52 | −1.26(−1.38-−1.13) |
| Qatar | 0.29(0.22-0.37) | 141.22(111.97-176.29) | 1.63(1.19-2.19) | 122.6(92.34-164.07) | 471.66 | −0.34(−0.55-−0.12) |
| Republic of Korea | 29.01(27.31-30.56) | 72.35(68.61-76.25) | 55.23(49.92-61) | 69.62(63.09-76.73) | 90.41 | −0.88(−1.17-−0.59) |
| Republic of Moldova | 3.43(3.14-3.76) | 76.35(69.81-83.64) | 3.92(3.3-4.59) | 88.06(75.74-101.82) | 14.16 | 0.45(0.16-0.74) |
| Romania | 20.81(19.45-22.12) | 84.36(78.58-90.31) | 23.12(18.64-28.5) | 87.06(70.81-107.17) | 11.12 | 0.23(0.11-0.35) |
| Russian Federation | 126.91(122.15-132.71) | 78.13(75.17-81.72) | 134.17(117.05-152.51) | 68.38(59.77-77.81) | 5.72 | −0.62(−0.86-−0.39) |
| Rwanda | 4.71(2.86-6.89) | 95.34(65.42-128.56) | 7.54(5.46-10.47) | 87.27(65.9-118.78) | 60.23 | −0.75(−0.98-−0.51) |
| Saint Kitts and Nevis | 0.08(0.07-0.09) | 222.15(195.81-252.25) | 0.09(0.07-0.12) | 136.58(97.31-177.74) | 12.5 | −1.41(−1.73-−1.09) |
| Saint Lucia | 0.25(0.23-0.28) | 228.33(207.95-253.14) | 0.35(0.28-0.43) | 174.3(141.25-212.43) | 40.32 | −1.05(−1.32-−0.78) |
| Saint Vincent and the Grenadines | 0.23(0.2-0.26) | 250.62(216.28-287.36) | 0.29(0.24-0.34) | 225.72(188.66-270.29) | 25.44 | −0.42(−0.56-−0.29) |
| Samoa | 0.09(0.06-0.12) | 73.88(50.76-100.36) | 0.13(0.08-0.19) | 76.55(49.61-106.59) | 51.68 | 0.19(0.14-0.23) |
| San Marino | 0.07(0.06-0.09) | 242.04(205.33-285.28) | 0.13(0.09-0.19) | 232.15(154.03-346.3) | 72.42 | 0.08(0-0.16) |
| Sao Tome and Principe | 0.09(0.07-0.11) | 80.94(61.25-97.42) | 0.14(0.1-0.2) | 92.1(65.54-124.67) | 62.28 | 0.15(−0.07-0.36) |
| Saudi Arabia | 11.34(5.64-15.49) | 126.04(63.02-170.1) | 32.96(25.12-42.17) | 125.13(98.74-156) | 190.67 | −0.35(−0.53-−0.18) |
| Senegal | 5.72(3.86-8.01) | 92.69(70.83-116.19) | 10(6.9-13.76) | 89.16(63.77-120.07) | 74.9 | −0.29(−0.44-−0.13) |
| Serbia | 9.82(8.31-11.5) | 92.78(78.1-109.32) | 12.47(9.79-15.72) | 94.46(74.33-119.07) | 26.96 | 0.35(0.21-0.49) |
| Seychelles | 0.07(0.07-0.08) | 120.13(108-132.73) | 0.2(0.17-0.23) | 174.32(152.43-198.82) | 165.8 | 1(0.8-1.2) |
| Sierra Leone | 2.85(1.64-4.48) | 85.74(58.23-119.6) | 5.35(3.7-7.45) | 83.09(57.82-113.44) | 87.69 | −0.27(−0.41-−0.13) |
| Singapore | 2.7(2.4-3.06) | 103.02(91.03-117.64) | 5.27(4.39-6.32) | 72.44(60.71-85.79) | 95.11 | −1.44(−1.61-−1.27) |
| Slovakia | 5.16(4.57-5.85) | 89.76(79.47-101.93) | 8.57(6.71-10.83) | 104.57(81.72-132.38) | 66.05 | 0.87(0.72-1.03) |
| Slovenia | 1.95(1.48-2.49) | 84.32(64.44-107.57) | 3.93(2.98-5.08) | 104.27(79.76-134.61) | 101.56 | 0.81(0.6-1.01) |
| Solomon Islands | 0.61(0.45-0.81) | 241.42(185.96-310.93) | 1.33(1.06-1.64) | 256.68(204.95-310.66) | 119.14 | 0.1(−0.01-0.22) |
| Somalia | 2.74(1.81-4.15) | 61.27(43.21-86.86) | 7.65(4.1-11.62) | 61.12(32.18-94.07) | 179.51 | 0.19(0.11-0.27) |
| South Africa | 34.53(31.52-38.01) | 114.77(104.26-124.94) | 63.12(55.56-72.01) | 116.6(103.36-131.68) | 82.76 | 0.25(−0.07-0.57) |
| South Sudan | 4.34(2-8.45) | 105.83(55.18-190.88) | 6.14(3.58-10.34) | 98.02(57.45-158.95) | 41.51 | −0.13(−0.27-0) |
| Spain | 55.95(53.33-58.83) | 119.17(113.59-125.29) | 66.98(58.85-75.76) | 85.05(75.64-95.78) | 19.71 | −1.68(−1.94-−1.42) |
| Sri Lanka | 7.77(6.82-8.7) | 54.96(48.87-60.98) | 17.7(12.98-23.47) | 71.64(53.14-94.53) | 127.72 | 1.29(1-1.58) |
| Sudan | 9.04(5.4-14.02) | 58.38(40.08-84.3) | 19.1(12.2-29.37) | 64.55(40.95-97.63) | 111.21 | 0.56(0.38-0.73) |
| Suriname | 0.45(0.35-0.5) | 132.15(107.75-146.39) | 0.77(0.62-0.93) | 127.59(103.57-154.85) | 72.16 | −0.16(−0.43-0.12) |
| Sweden | 16.81(15.75-17.85) | 128.39(120.23-136.26) | 16.48(14.68-18.49) | 86.56(77.53-96.91) | −1.96 | −1.83(−2-−1.65) |
| Switzerland | 5.35(4.91-5.8) | 58.86(54.37-63.54) | 12.7(11.2-14.56) | 82.09(73.15-92.75) | 137.52 | 0.12(−0.73-0.98) |
| Syrian Arab Republic | 6(4.45-7.76) | 74.85(56.43-95.43) | 6.51(4.64-8.8) | 46.96(33.78-63.24) | 8.46 | −2.58(−3.17-−1.98) |
| Taiwan  (Province of China) | 21.76(20.12-23.48) | 116.59(107.92-125.83) | 35.5(26.57-46.76) | 103.43(77.59-135.82) | 63.11 | −0.7(−0.87-−0.52) |
| Tajikistan | 1.64(1.48-1.83) | 40.61(36.66-45.39) | 4.93(3.99-6.13) | 64.52(53.02-78.77) | 200.33 | 1.72(1.37-2.08) |
| Thailand | 25.83(22.58-29.27) | 53.74(46.89-60.69) | 53.19(39.28-69.89) | 59.07(44.61-76.61) | 105.95 | −0.29(−0.52-−0.05) |
| Timor-Leste | 0.36(0.2-0.55) | 59.06(40.24-80.79) | 0.72(0.49-0.94) | 69.06(48.12-88.96) | 103.09 | 0.73(0.41-1.04) |
| Togo | 2.29(1.67-3.03) | 85.73(70.73-104.4) | 4.95(3.47-6.7) | 83.52(58.86-112.63) | 116.49 | −0.54(−0.75-−0.33) |
| Tokelau | 0(0-0) | 63.24(47.7-83.14) | 0(0-0) | 70.49(49.27-98.23) | 3.2 | 0.39(0.35-0.44) |
| Tonga | 0.09(0.08-0.11) | 128.15(106.81-152.44) | 0.12(0.09-0.17) | 140.96(107.87-186.45) | 34.84 | 0.18(0.08-0.29) |
| Trinidad and Tobago | 1.71(1.54-1.9) | 160(144.55-176.81) | 1.91(1.34-2.59) | 118.54(83.5-160.1) | 11.29 | −1.28(−1.47-−1.08) |
| Tunisia | 2.39(1.97-2.81) | 36.55(30.34-42.27) | 5(3.66-6.78) | 39.12(28.9-52.66) | 108.79 | 0.19(0.14-0.23) |
| Turkey | 61.44(45.18-77.76) | 126.34(94.41-157.87) | 82.33(66.32-100.47) | 94.09(76.2-113.83) | 33.99 | −1.03(−1.28-−0.78) |
| Turkmenistan | 1.11(0.97-1.26) | 37.7(33.24-42.85) | 2.23(1.75-2.85) | 47.87(37.72-60.93) | 101.61 | 0.96(0.68-1.25) |
| Tuvalu | 0.01(0-0.01) | 75.16(57.97-94.47) | 0.01(0.01-0.01) | 75.91(51.34-104.63) | 35.36 | 0(−0.02-0.01) |
| Uganda | 4.81(3.6-6.28) | 43.61(34.8-52.46) | 20.14(15.03-26.96) | 72.26(55.93-91.32) | 319.08 | 2.05(1.90-2.20) |
| Ukraine | 33.28(30.93-35.84) | 58.2(54-62.61) | 55.02(45.32-66.12) | 100.16(82.41-119.04) | 65.33 | 2.09(1.71-2.48) |
| United Arab Emirates | 2.06(1.09-3.45) | 183.13(80.96-315.7) | 13.61(7.14-21.19) | 147.51(77.16-222.57) | 561.34 | −0.87(−1.08-−0.66) |
| United Kingdom | 110.57(107.47-113.22) | 140.37(136.81-143.51) | 124.02(117.43-129.97) | 111.5(106.53-116.52) | 12.16 | −1.31(−1.51-−1.12) |
| United Republic of Tanzania | 19.42(13.1-26.1) | 110.47(86-138.77) | 46.51(37.01-58.46) | 125.63(103.99-150.39) | 139.56 | 0.64(0.5-0.78) |
| United States of America | 531.56(515.43-544.62) | 177.7(172.85-181.86) | 607.66(576.39-635.34) | 118.14(112.84-123.28) | 14.32 | −2.09(−2.31-−1.86) |
| United States Virgin Islands | 0.16(0.13-0.19) | 158(131.83-187.82) | 0.23(0.19-0.28) | 149.66(119.87-188.93) | 44.66 | −0.31(−0.41-−0.2) |
| Uruguay | 5.59(5.23-5.97) | 159.91(149.41-170.86) | 6.99(6.31-7.75) | 156.73(141.38-174.51) | 24.9 | −0.28(−0.46-−0.11) |
| Uzbekistan | 7.24(6.55-7.97) | 41.65(38.04-45.68) | 24.11(19.39-29.1) | 79.36(64.23-95.44) | 233.08 | 2.99(2.59-3.39) |
| Vanuatu | 0.06(0.04-0.08) | 56.79(41.24-74.65) | 0.17(0.11-0.23) | 71.13(48.49-96.19) | 188.1 | 0.58(0.46-0.71) |
| Venezuela | 17.86(16.25-19.41) | 120.61(110.12-131.41) | 27.38(20.09-36.61) | 93.29(68.67-124.23) | 53.29 | −1.13(−1.33-−0.92) |
| Viet Nam | 30.43(23.74-39.56) | 59.46(46.99-75.79) | 59.9(46.16-75.18) | 60.45(47.67-74.92) | 96.83 | 0.08(−0.09-0.26) |
| Yemen | 4.58(2.63-6.89) | 54.98(36.83-77.62) | 11.34(7.75-15.43) | 54.73(37.92-74.69) | 147.39 | 0.3(0.18-0.41) |
| Zambia | 6.49(3.77-9.35) | 127.5(93.88-164.64) | 13.3(10.3-17.09) | 130.99(104.73-162.9) | 104.85 | 0.07(0.04-0.09) |
| Zimbabwe | 3.93(3.26-4.6) | 62.55(52.64-72.33) | 4.03(2.98-5.47) | 40.25(30.11-52.9) | 2.56 | −2.00(−2.39-−1.6) |

EAPC: estimated annual percentage change; ASR, age-standardized rate; CI, confidence interval; UI: uncertainty interval.
